# Strategy for hybrid connection of porous strut and triply periodic minimal surface lattice structures

**DOI:** 10.1038/s41467-026-77560-7

**Published:** 2026-10-05

**Authors:** Jianxing Yang, Vahid Moosabeiki, Long Bai, Mauricio Cruz Saldívar, Marius A. Leeflang, Xiaohong Chen, Amir A. Zadpoor, Mohammad J. Mirzaali

**Affiliations:** 1 College of Mechanical Engineering and Automation, Huaqiao University, Xiamen, China; 2 Faculty of Mechanical Engineering, Department of Biomechanical Engineering, Delft University of Technology (TU Delft), Delft, The Netherlands; 3 College of Mechanical and Vehicle Engineering, Chongqing University, Chongqing, China

**Keywords:** Mechanical properties, Composites

## Abstract

Hybrid design approaches are essential for developing porous lattice structures with integrated multifunctional performance. While previous studies have focused on hybridization among different topologies, combining distinct structure types—such as integrating the high mechanical strength of triply periodic minimal surface (TPMS) sheet structures with the meta-structural characteristics of strut-based architectures—remains underexplored due to challenges in creating effective connections between these disparate forms. Here, we introduce a design strategy for hybridizing TPMS sheet and strut lattice structures via twinned unit cells. These transition substructures have tunable properties enabling modulation of the transition cell modulus from 0.4 to 5 times its original value and direct generation of 3D-printable files. This approach enables a more effective balance between multiple mechanical properties. Prototypes demonstrate successful hybridization and multifunctional performance, achieving both geometric and mechanical compatibility. Notably, the hybrid structures exhibit a 37- to 84-fold variation in structural stiffness, addressing complex requirements, such as bone-to-tendon interfaces, that are challenging for previously reported strategies.

## Abstract (web summary)

A hybrid strategy connects two porous structure types via twinned cells, integrating their strengths. It enables on-demand stiffness tuning and balanced multi-property performance. Prototypes confirm seamless integration, bone-to-tendon compatibility, and 3D printability.

## Introduction

Engineered architected lattice materials, also referred to as designer materials or mechanical metamaterials^1,2^, have attracted considerable attention because their mechanical and functional properties can be programmed through geometry rather than composition. This design paradigm enables unprecedented combinations of properties that are difficult or impossible to achieve with conventional materials, leading to applications across fields ranging from biomedical implants to lightweight structural systems in the aerospace and automotive industries. A central challenge in the design of these materials is the rational optimization of micro-architectural geometry to achieve targeted mechanical and functional performance^3^.

Architected lattices are commonly classified into two main categories, namely strut-based and sheet-based architectures. Strut-based lattices consist of discrete beam elements arranged in periodic networks and typically exhibit bending or stretch-dominated deformation behavior^4,5^. Sheet-based architectures, in contrast, are constructed from continuous surfaces with finite thickness. Among them, triply periodic minimal surfaces (TPMS) represent a particularly important class of structures that form continuous, self-supporting networks with advantageous mechanical characteristics, including high specific stiffness and specific strength, as well as controllable anisotropy^6–9^. TPMS sheet architectures also offer large surface-to-volume ratios^10^, which is beneficial for applications involving surface-driven interactions, such as biomaterials designed to regulate cell adhesion and growth^11^. Strut-based lattices provide greater topological freedom, enabling the realization of unusual mechanical responses, including negative stiffness^12^, negative Poisson’s ratio^13–15^, high flexibility^16^, adaptability^17,18^, and tunable stiffness^19^. As a result, sheet-based and strut-based lattices offer complementary functional advantages.

To tailor the mechanical behavior of architected materials, researchers have widely explored functional gradient strategies, in which structural parameters vary gradually throughout the architecture. These gradients can be implemented through changes in strut diameter or surface thickness^20,21^, scaling of unit cells^22^, or controlled deformation of unit cells through compression or elongation^23–25^. Such approaches typically generate transitions between identical or closely related architectures^26,27^. Gradient strategies employing linear^28,29^, step-wise^30,31^, or exponential^32,33^ transitions have been shown to enhance the mechanical performance of architected structures in diverse applications^34^, including improved energy absorption in crash-protection systems^31,35^ and the modulation of mechanical cues in bone tissue scaffolds^36,37^ to mitigate stress shielding and promote bone regeneration^38^. TPMS architectures are particularly well suited to such gradient designs because they are defined by continuous mathematical surfaces^39–43^. In contrast, strut-based lattices generally lack a continuous analytical description, which makes the realization of smooth geometric transitions considerably more challenging.

However, many natural and engineered systems require abrupt, dramatic transitions in structural and mechanical properties, rather than gradual gradients. A prominent example is the bone-tendon interface, where a stiff, porous bone structure connects to a highly compliant fibrous tendon within a relatively short transition zone^44^. Similarly, engineered porous components that must simultaneously provide high load-bearing capacity and conformal compliance may require integrating stiff core architectures with highly deformable boundary regions^17^. In such cases, the optimal structural solution may involve combining fundamentally different lattice topologies, for example, a high-stiffness TPMS sheet architecture with a compliant strut-based lattice. Conventional gradient strategies are poorly suited to such problems. Achieving large property contrasts within a single topology typically requires drastic geometric modifications that produce extreme feature sizes^45^, introduce manufacturing constraints^46^, or generate severe stress concentrations^47^.

To address these challenges, geometric interpolation approaches have been proposed to blend distinct lattice architectures^48,49^. In these methods, distance-field-based weighting functions^50,51^ are typically used to generate intermediate geometries between two structures. Although such interpolation techniques expand the design space and enable spatially varying architectures^52–54^, they suffer from important limitations. The geometry of the transitional region is often difficult to control, making the mechanical behavior of the resulting structure difficult to predict. Moreover, when the underlying topologies differ significantly, interpolation can generate irregular or extreme geometric features that compromise manufacturability and structural performance. Recent studies^55^ have attempted to alleviate these issues by replacing direct interpolation with dual-architecture superposition strategies to construct transitional unit cells. Despite these advances, robust strategies for directly connecting fundamentally different lattice topologies remain challenging and largely unexplored.

These challenges partly explain why hybrid architectures combining TPMS sheet structures and strut-based lattices remained relatively underdeveloped, despite the extensive investigation of each topology individually^56,57^. The fundamental difficulty lies in creating a smooth, mechanically robust, and geometrically compatible interface between these two structurally distinct systems.

**Fig. 1:**
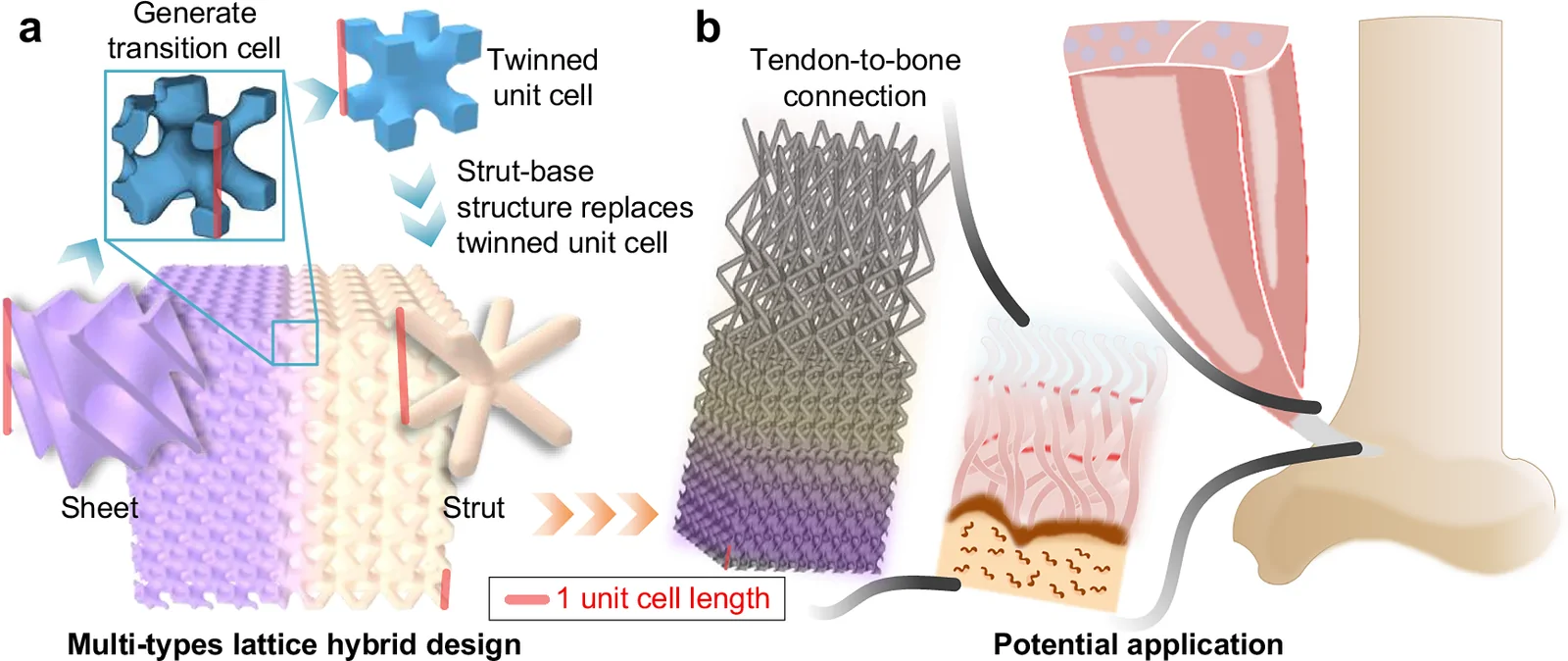
Proposed strategy for hybrid connection of porous lattice structures. **a** Schematic illustration of the hybrid design principle. The transition between a TPMS sheet structure (left) and a strut-based lattice (right) is achieved through a twinned unit cell approach. **b** Potential application of multi-types lattice hybrid design: a biomimetic tendon-to-bone connection structure. The hybrid design integrates a porous bone-like region (TPMS sheet), a transitional interface, and a fibrous tendon-like region (strut-based lattice) within a single continuous structure, illustrating the capability of the proposed strategy to address complex interfaces with graded mechanical properties. The scale hierarchy spans from the unit cell level to the application level (schematic representation, not to scale).

Here, we address this challenge by exploiting structural correspondences between TPMS skeletal representations and strut-based lattices. We identify three TPMS skeletal configurations^3^ whose boundary node characteristics closely resemble those of conventional strut-based lattices and use them as twinned unit cells to construct transition elements. Because the nodal connectivity of these skeletal forms is compatible with that of strut-based networks, they provide a natural geometric bridge between continuous TPMS sheets and discrete strut architectures. The resulting transition elements therefore enable smooth connections between TPMS sheet structures and their corresponding strut-based lattices (Fig. 1a). Building on this concept, we develop a computational framework that combines voxel-based modeling^58,59^, control-point-driven geometric manipulation^60^, and compensated optimization, allowing the generation of hybrid architectures without relying on conventional array-based lattice construction^61^. The resulting geometries can be directly translated into manufacturable 3D models.

We validate the proposed framework through a combination of experiments and numerical simulations. Mechanical testing was used to evaluate how different connection strategies influence stiffness, failure mechanisms, and fatigue behavior of the hybrid lattices, while numerical simulations were employed to explore the broader parameter space within experimentally validated limits. Finally, we demonstrated the approach’s potential through several application-driven examples, including the design of a biomimetic tendon-to-bone interface (Fig. 1b).

## Results and discussion

### Morphological characteristics of hybrid structures

The size of the hybrid structure was defined by its design domain, which consisted of two subdomains representing the strut-based and sheet-based architectures to be integrated. These two design domains were described using the spatial occupancy of two three-dimensional models in the STL format, sharing the same coordinate system. During structure generation, an extended rectangular prism was constructed based on the maximum dimensions of the design domain along the coordinate axes (assuming length, width, and height as *L*_*D*_, *B*_*D*_, and *H*_*D*_, respectively).

Subsequently, the entire design domain was discretized into a matrix representation with a given resolution, determined by the number of voxels ($n_{u}$) contained in the edge length (${L}_{u}$) of each unit cell. The total number of elements in the matrix was given by ${N=L}_{D}{B}_{D}{H}_{D}/{({L}_{u}/{n}_{u})}^{3}$. Different structural types within the design domain were assigned distinct values. A unit matrix consisting of ${n}_{u}^{3}$ elements was iteratively moved from the origin of the coordinate system to search the global design domain, identifying the connection boundaries between the two structural types. The elements within each ${n}_{u}^{3}$ unit matrix containing a boundary were extracted to define the transition unit cell’s design domain and assigned a new value.

The corresponding structure was then generated based on the values of elements within different regions of the design domain. The diameter of the strut or the wall thickness of sheet-based structures in the transition unit cell was set equal to the corresponding values of the adjacent strut-based or sheet-based structures at the same location, ensuring smooth connectivity. This also helped maintain the relative density within a similar range as the neighboring structures. Finally, a Boolean intersection operation was performed between the generated rectangular prism structure and the initial design domain geometry to obtain the final hybrid structure that conformed to the prescribed geometric constraints.

**Fig. 2:**
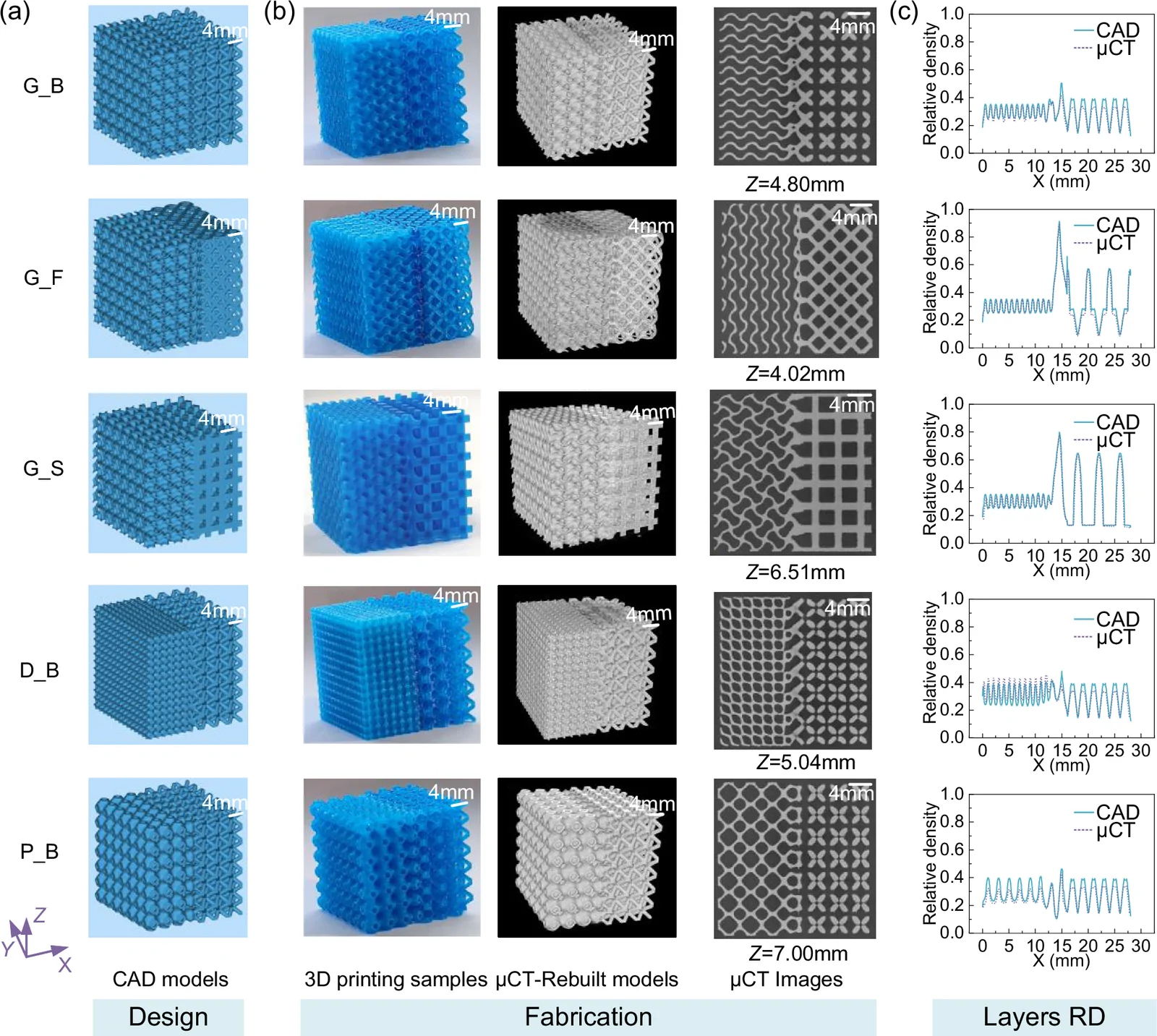
Fabricated hybrid lattice specimens and their quality analysis. **a** CAD designed specimens showing the geometric transition between TPMS sheet and strut-based lattice architectures across the connection interface. **b** Polyjet 3D printed specimens fabricated using the proposed design framework (left), 3D reconstructed micro-computed tomography (µCT) scans of the printed framework (middle), and µCT cross-sectional views at the connection interfaces between two different lattice architectures (TPMS sheet and strut-based), showing connectivity at the interfaces of two different lattice architectures (right). **c** Overall relative density variation along the X direction. Source data are provided as a Source Data file.

The hybrid structure specimens were successfully 3D printed, with the print quality of each constituent structure closely resembling that of its corresponding individual counterpart. As a result, only the hybrid structures were evaluated using micro-computed tomography (μCT) scans. The 3D printed specimens, 3D models reconstructed from μCT images, and 2D scan images exhibit a high degree of fidelity to the original design model (Fig. 2a, b), particularly in terms of connection characteristics at the interfaces of two distinct lattice architectures. While some rounded features were observed at the topmost layer of the printed specimens due to liquid tension and gravity (Fig. 2b), these effects were minimal and did not significantly impact subsequent experiments. Furthermore, the 2D CT scan images confirmed the excellent print quality of the structure, showing no visible voids. The smooth and continuous transition between connected unit cells further demonstrated the effectiveness of the proposed hybridization strategy in integrating TPMS sheet and strut-based structures.

Additionally, the hybrid structures exhibited consistent layer-by-layer relative density trends along the hybrid direction (*X*-direction in Fig. 2), as measured from both the CAD design and μCT reconstruction models. The strong agreement between the actual relative densities and the design values near ${X}=14\,{\rm{mm}}$ suggests precise manufacturing of the connection regions. In the G-series portion (${X} < 12\,{\rm{mm}}$), with the μCT-scan results are only slightly lower than the design values, suggesting a high level of printing accuracy achieved through Polyjet technology.

In contrast, the D- and P-series portions of the B_D and B_P structures exhibited larger deviations, indicating lower printing accuracy (last two rows in Fig. 2c). The actual relative density of the D-series structures significantly exceeded the design value, likely due to the structure’s complexity and thin-walled features, which increased material accumulation (14 %). Conversely, the actual relative densities of the P-series structures were significantly lower than the design values (15 %), possibly because their geometry closely resembled strut-based designs, as evidenced by the reduced actual relative densities in the strut-based portions (${X} > 16\,{\rm{mm}}$) across all structures (last row in Fig. 2c).

Despite these variations, the BCC structural components within all three B-series hybrid structures exhibited consistent deviations of similar magnitude (6.5–9.5% decrease, first and last two rows in Fig. 2c). This consistency, along with the stable trends observed in the G-series structures, confirms that the printing process demonstrated a high degree of reproducibility, ensuring uniformity across multiple specimens. This reproducibility was further validated by the quality analyses of individual structural specimens, as detailed in Supplementary Note 1, Supplementary Fig. 1 and Supplementary Table 1.

### Mechanical response of hybrid interfaces

**Fig. 3:**
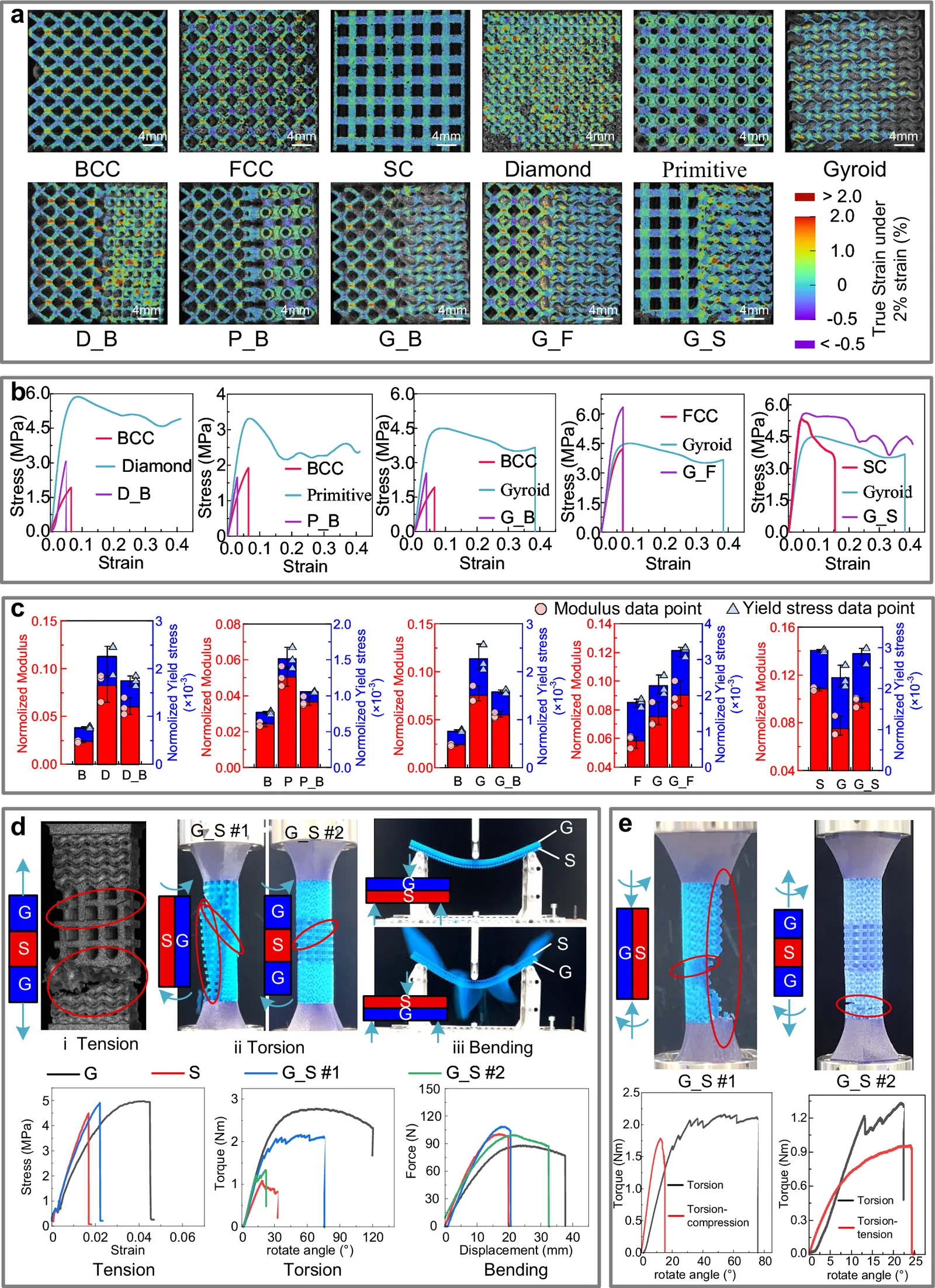
Comparative experimental characterization of hybrid lattice structures under multiple loading conditions. **a** Full-field strain distribution maps obtained from digital image correlation (DIC) during uniaxial compression testing at 2% global strain. The color contours represent the local strain magnitude. **b** Corresponding stress-strain curves, which illustrate the full deformation response. For curves where post-failure data were not recorded by the testing machine, the final data point at fracture was manually set to zero. This addition is made for visual clarity and does not represent measured data beyond the point of structural collapse. **c** Comparison of normalized mechanical properties (modulus, yield stress) across different hybrid configurations. All values are derived from three independently tested specimens per configuration. Error bars represent the full range (minimum to maximum) of the data (*n* = 3). **d** The failure snapshots of the G_S hybrid structure under tensile, torsional, and bending loading modes, captured at the point of fracture. The corresponding stress/torque/force versus strain/angle/displacement curves are shown alongside each image. **e** Failure snapshot and the corresponding torque versus angle curve under biaxial loading. The red circles in (**d**, **e**) indicate the locations of structural fracture/damage observed during mechanical testing. Source data are provided as a Source Data file.

The mechanical responses of the constituent strut-based lattices (i.e., BCC, FCC, and SC) in Fig. 3a exhibited notable differences under compressive loading (see Supplementary Fig. 2 for the repeatability study, and Supplementary Movies 1 and Movies 2 for the complete compression tests). The stress-strain curves showed that the SC structure has the highest compressive modulus of elasticity and yield strength, while the BCC structure exhibited the lowest values (Fig. 3b). This suggests that as the angle between the strut axis and the load direction approaches 0° (i.e., greater parallel alignment), the load-carrying capacity of the strut-based structure increases. Notably, this angle is smallest in the SC structure, followed by the FCC and BCC. The strain distribution maps further confirm this trend: the “×”-shaped intersections in the BCC and FCC structures and the “+”-shaped intersections in the SC structure (Fig. 3a) showed higher strain concentrations found at the junctions of these strut-based structures.

In contrast, the three TPMS sheet structures exhibit distinct mechanical properties (Fig. 3c). Among them, the D structure demonstrated the highest compressive modulus and yield strength, followed by the G structure, while the P structure exhibited the lowest properties. However, despite these differences, TPMS sheet structures generally exhibited superior damage resistance as compared to their strut-based counterparts (Fig. 3c). Unlike the BCC, FCC and SC structures, which experiment complete failure at strains below 15%, the D, G, and P structures endured strains of up to 40%, demonstrating improved resilience (Fig. 3c). Their stress-strain curves exhibited a plateau effect after reaching peak stresses, particularly in the D and P structures, which sustained compressive loading up to 40% strain without catastrophic failure (Fig. 3c). Strain distribution analysis further showed that high-strain regions were concentrated on the thinner surfaces of the TPMS structures (Fig. 3a). Additionally, strain distributions in the D and G structures exhibited a clear directional pattern, forming 45°-inclined shapes (red zones in Fig. 3a). As compression progressed, these strain distribution patterns remained consistent, with only a gradual increase in magnitude (Supplementary Fig. 3 and Supplementary Note 2).

The integration of TPMS sheets and strut-based structures into hybrid designs does not significantly alter their inherent strain distribution characteristics under compressive loading. The strain patterns of hybrid structures remained largely consistent with those of individual substructures under similar loading conditions (Fig. 3a). However, a key focus of this study was the strain behavior at the hybrid structure connection regions. The transition zones of the D_B, P_B, and G_B hybrid structures exhibited low strains (Fig. 3a), suggesting that the designed connections effectively mitigate stress concentrations at these interfaces. In contrast, the G_F and G_S hybrid structures displayed pronounced strain concentrations in their transition regions, resembling the high-strain behavior observed in their individual substructures (Fig. 3a). This indicates that while some connection designs effectively minimize strain concentrations at the junctions of the hybrid structure, others inherit the mechanical characteristics of their constituent structures. Nonetheless, the design approach proposed in this study prevent excessive strain accumulation at the joints, reducing the likelihood of premature failure.

The deformation and failure progression of the hybrid structure (Supplementary Fig. 4) further support this observation. As compression increases, the damage patterns in hybrid structures closely resemble those of their individual substructures, rather than being concentrated in the transition zones (see Supplementary Note 3 for details). This highlights the importance of maintaining functional integrity in hybrid substructures, ensuring that the transition regions do not become the primary failure sites, which is an essential consideration in the design of structurally efficient hybrid lattices.

The mechanical properties of most hybrid structures fall within the range defined by their constituent substructures (Fig. 3b). An exception is the G_F hybrid structure, which demonstrates superior mechanical properties as compared to both of its constituent components. This behavior is further confirmed in Fig. 3c, where its compressive modulus and yield strength exceed those of the individual structures. This enhancement can be attributed to the Neovius-skeletal transition connections^62^, which share nodal characteristics with the FCC structures and are integrated with the G-sheet structures. To ensure proper alignment with the FCC strut diameter, the Neovius-based transition structure requires a higher relative density of 0.5, while the overall structure has a relative density of 0.3, resulting in an overall increase in mechanical properties. Notably, the transition region (i.e., comprising 1/7 of the total structure) plays a significant role in enhancing the mechanical performance of the hybrid structure. While reducing the density of the transition section can mitigate its impact, an alternative approach is to identify transition structures with similar mechanical characteristics to optimize the design. In this study, we also proposed a method for further optimizing these properties a modulating the stiffness of the transition unit cells (Section 2.3).

Moreover, hybridization approaches often result in structural responses that are of a low-energy-absorption nature when combining inherently low-energy-absorption (i.e., early-failure) constituents with high-energy-absorption constituents. This occurs because low-energy-absorption lattice structures generally exhibit lower stiffness (e.g., BCC or FCC architectures typically possess lower elastic modulus than the TPMS structures with which they are combined) and therefore reach their failure threshold earlier, initiating early fracture. However, the experimental results for the G_S hybrid structure in Fig. 3b provide evidence that both high energy absorption and high stiffness can be achieved simultaneously. In this configuration, the TPMS component exhibits lower stiffness than the strut-based component, suggesting a potential design guideline for applications requiring high energy absorption: the stiffness of the low-energy-absorption constituent should be controlled to exceed that of the high-energy-absorption constituent, thereby enabling synergistic enhancement of both stiffness and energy-absorbing capacity. It should be noted, however, that such a phenomenon occurs only when low-energy-absorption materials are used. In ductile materials, this issue does not arise.

To further evaluate the robustness of the proposed design under different loading conditions, additional mechanical tests were performed under tension, torsion, bending, and combined loading (Fig. 3d, e, Supplementary Fig. 5, and Supplementary Note 4, and Supplementary Movies 5–7, repeat test curve are shown in Supplementary Fig. 6). In all cases, failure consistently occurred within the constituent substructures rather than at the connecting interfaces, indicating that the transitional structures effectively resist deformation-induced failure (Supplementary Fig. 5a, c, e, f).

For example, under tensile loading, the G-S hybrid structure exhibited complete fracture in the G substructure region (lower red circle in Fig. 3d, i) and visible cracks in the S substructure region (upper red circle in Fig. 3d, i), while no significant damage was observed in the transition region. When subjected to torsion, the S structure was prone to bending-shear coupling failure, with stress concentrating at the nodes, leading to brittle fracture. In contrast, the curved connectivity of the G structure enabled uniform stress distribution, achieving large deformation and energy absorption through cell wall bending and buckling (Supplementary Fig. 5a). In the hybrid G-S structure, the transition region alleviated stress concentration by dispersing localized stress into adjacent cells, resulting in substantial fracture first occurring within the S substructure region (left red circle in Fig. 3d, ii). Following this fracture, the transition region delayed crack initiation, and a further increase in the torsion angle was required to induce cracks in the G substructure region, ultimately leading to complete fracture (right red circle in Fig. 3d, ii). Compared to the series hybrid configuration (G-S#2), this compromise effect was more pronounced in the parallel hybrid configuration (G-S#1).

Under bending loading, although both single and hybrid structures exhibited sudden fracture (making it difficult to capture the fracture moment in Supplementary Movie 7; the images shown are screenshots with motion blur before and after fracture, Supplementary Fig. 5h), it was evident that the hybrid structure with the G component placed near the indenter (G-S#1) exhibited a bending deflection closer to that of the S structure, whereas the hybrid structure with the opposite arrangement (G-S#2) showed a bending deflection more similar to that of the G structure. This demonstrates the significance of hybridizing G and S structures, as their different configurations yield varying levels of bending resistance. In other words, by strategically arranging G and S structures, it is possible to program structural performance to meet engineering requirements.

Furthermore, the deformation curves further reveal that the G structure exhibits greater fracture strain or displacement under tensile and bending loads, demonstrating superior fracture toughness, whereas the S structure possesses a higher elastic modulus, indicating a greater load-bearing capacity (Fig. 3d). By hybridizing these two structures through the proposed method, a balanced mechanical response is achieved, integrating the advantages of the constituent substructures to obtain moderate fracture toughness and load-bearing capacity. This approach overcomes the inherent trade-off between stiffness and energy absorption that is commonly encountered in traditional lattice designs. Under torsional loading, because the S structure is not well-suited to resisting torsion, its presence in the parallel hybrid configuration (G-S#1) accounts for half of the structure, significantly reducing the torsional resistance of the G structure. In the series hybrid configuration (G-S#2), failure is governed by the weakest part, where the failure of the S structure directly leads to structural disconnection. Although neither the series nor the parallel hybrid structure surpasses the performance limit of the G structure, they still exhibit a compromise in performance, characteristic of hybrid configurations.

Under biaxial loading conditions, it is observed that, in the parallel configuration (G_S #1) under compressive-torsional loading, failure predominantly occurs in the S-shaped region. All S-shaped structures undergo instantaneous fracture at the failure strain, with damage rapidly propagating into the transitional structure and the G-shaped region. In contrast, for the series configuration (G_S #2) under tensile-torsional loading, the fracture morphology resembles that observed under pure tensile loading, with failure mainly localizing in the G-shaped region rather than exhibiting the shear-dominated failure characteristics of the S-shaped region seen under pure torsion. These findings indicate that under biaxial loading, tension and compression dominate structural failure, and failure behavior is governed by the load-bearing capacity of the constituent structures. The transitional structure does not fail first and effectively serves as a functional connector. Further statistical analysis of the torque–rotation angle curves reveals that under compressive-torsional loading, the G_S #1 configuration can sustain higher torque at smaller rotation angles (i.e., higher torsional stiffness), requiring greater resistance to failure. However, due to the dominance of compressive loading, complete failure occurs at an earlier stage.

### Finite element simulation results of hybrid structures and their stiffness range modulation

**Fig. 4:**
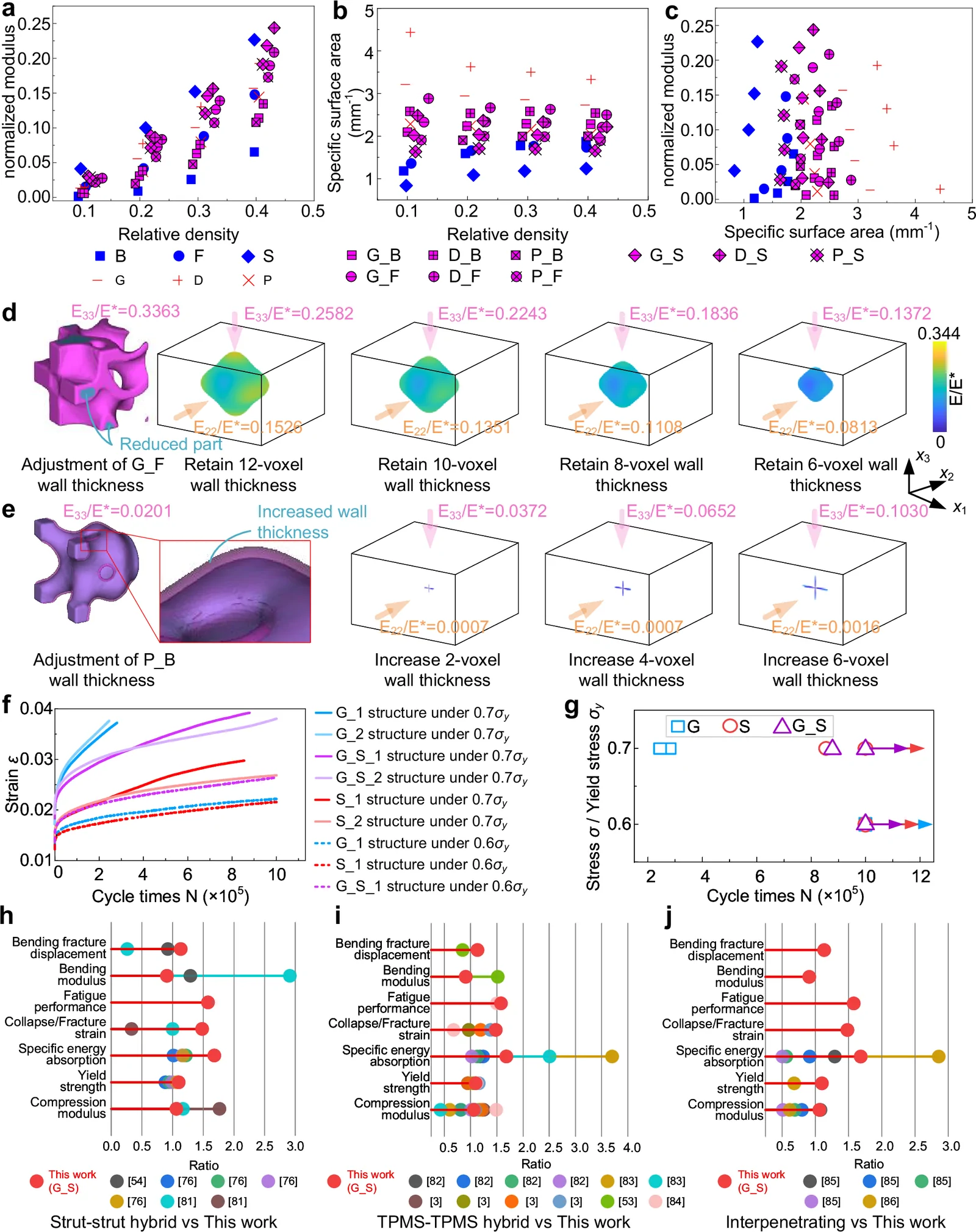
Numerical simulation results and fatigue response of hybrid structures. **a**–**c** Relationship between normalized modulus, specific surface area, and relative density. **a** Normalized modulus versus relative density. **b** Specific surface area versus relative density. **c** Normalized modulus versus specific surface area. **d** Modulus changes of transition cells by voxel erosion. The erosion process removes surface layers from the TPMS skeletal elements, resulting in a progressive decrease in the effective modulus of the transition unit cell. **e** Modulus changes of transition cells by voxel dilation. The dilation process adds surface layers, increasing the effective modulus. **f** Fatigue test results showing strain as a function of the number of cyclic loads for the hybrid structures under cyclic compression. **g** Distribution of the cycle times when the structure reaches 50% stiffness loss. **h**–**j** Lollipop chart comparison of the proposed hybrid structures (this study, red) with existing hybrid architectures from the literature. **h** Comparison with strut–strut hybrid structures (refs. ^45,54,76^). **i** Comparison with TPMS–TPMS hybrid structures (refs. ^3,53,81–83^). **j** Comparison with multi-topology interpenetrating hybrid structures (refs. ^84,85^). The value for each property represents the ratio obtained by dividing the performance value of the hybrid structure by the average performance value of its corresponding constituent single structures (*E*_C_: Compression modulus; *σ*_y_: Yield strength; SEA: Specific energy absorption; *ɛ*_*f*_: Collapse/Fracture strain; *N*_*f*_: Fatigue performance; *E*_B_: Bending modulus; *D*_BF_: Bending fracture displacement). This study evaluates multiple characteristics, while only the reported properties of the compared structures are extracted. Some data are taken from the figures presented. Source data are provided as a Source Data file.

To further investigate stiffness range modulation, we conducted quasi-static compressive finite element analyses FEA simulations (proof of validity is shown in Supplementary Note 5 and Supplementary Fig. 7) on the three strut-based, three TPMS sheet, and nine hybrid structures (i.e., a total of 15 structures, including four additional hybrid structures that were not experimentally tested). These simulations were performed at four different relative densities (i.e., 10%, 20%, 30%, and 40%). Most hybrid structures (shown in magenta and formed by combining the geometric patterns of the constituent topologies) exhibited mechanical properties that fall between those of their constituting substructures. The black blocks represent the strut structures, while the red wireframes denote the TPMS structure, as shown in Fig. 4a–c. For instance, at an equivalent relative density of 0.3, the G_B hybrid structure demonstrated a normalized modulus (0.0629) and specific surface area (2.2900) that lie between those of the B and G structures; the former ranges from 0.0256 to 0.1003, while the latter ranges from 1.7728 to 2.8517 (Fig. 4a, b). However, hybrid structures incorporating FCC lattices tend to exhibit higher-than-expected normalized moduli (0.1262), surpassing those of their individual components at comparable relative densities. This aligns with prior observations that the transition regions in FCC-based hybrid structures feature higher relative densities, which contribute to their enhanced mechanical properties (Fig. 2c).

At equivalent relative densities, strut-based structures (blue blocks in Fig. 4) generally exhibit lower specific surface areas than TPMS sheet architectures (red wireframes in Fig. 4b). Hybrid structures (shown in purple in Fig. 4b), however, demonstrated an intermediate specific surface area, effectively bridging gaps that would be challenging to address using a single structure type. Similarly, the normalized modulus range of hybrid structures helps fill the gap between strut-based and TPMS sheet structures when compared at equivalent specific surface area (Fig. 4c), expanding the envelope of achievable mechanical properties.

Hybrid structures in which the stiffness of the transition unit cells is intermediate between those of the constituent substructures are often preferred, as they facilitate efficient force transfer while minimizing stress concentrations. In this regard, transition unit cells with a higher elastic modulus prevent premature damage at the connection points. They might also hinder force transmission, potentially leading to localized stress buildup elsewhere. The transition unit cells with a lower elastic modulus can increase the risk of failure at the transition interface if they are too weak. However, the failure behavior can also be influenced by additional mechanical factors, including load distribution, geometric variations, and material properties.

These results emphasize the importance of careful stiffness modulation in hybrid lattice structures, ensuring that transition regions effectively balance mechanical compatibility, load transfer efficiency, and structural integrity. To further refine the mechanical performance of hybrid structures, we explored stiffness-modulation techniques by analyzing normalized modulus surfaces of transition cells and constituent structures (Supplementary Note 6 and Supplementary Fig. 8). A key strategy proposed here was to regulate the wall thickness of the transition structures through voxel erosion and dilation, allowing fine-tuned control over the structural relative density (see Supplementary Note 7 and Supplementary Fig. 9 for details), and thereby adjusting the stiffness (Fig. 4d, e).

In cases where the transition cell modulus exceeds that of the neighboring constituent structures, we introduce voxel erosion, a technique that selectively removes material from the thicker regions of the transition cell. Voxel erosion removed materials from the blue region of the original structure, resulting in the formation of the pinkish-purple structure (Fig. 4d). To maintain structural integrity, materials were preserved along the two boundary planes in the ${x}_{2}$ direction. By adjusting the remaining wall thickness, the relative density of the transition cell can be modulated, thereby influencing its stiffness. Computational results indicated that reducing wall thickness (with relative density decreasing from 52.32 to 35.49%) leads to a significant decline in the normalized modulus in the ${x}_{3}$ direction, from an initial 0.3363 (uncorroded state) to 0.1372 (i.e., approximately 40% of its original value) (Fig. 4d). This approach allows the transition cell’s mechanical properties to be tuned to align with those of adjacent structures.

Conversely, in cases where the transition cell was less stiff than its constituent structures, we proposed voxel dilation, a technique that increases wall thickness to enhance mechanical properties (Fig. 4e). The transparent region in Fig. 4e, represents the dilated wall. Similar to voxel erosion, material thickening was restricted along the ${x}_{2}$ boundary planes to maintain connectivity. Following dilation, the compressive modulus of the transition cell increased proportionally with the wall thickness. For instance, adding 6 voxels (increasing relative density from 27.10% to 57.34%) resulted in a 5-fold increase in the normalized modulus in the ${x}_{3}$ direction (Fig. 4e). By fitting these computational results, it was possible to accurately predict the stiffness of the transition structure and, through rule-of-mixture calculations, estimate the overall mechanical properties of the hybrid structure (detailed analysis in Supplementary Note 8 and Supplementary Fig. 10).

### Fatigue behavior of hybrid lattices

Previous studies^63^ have revealed that fatigue mechanics depend on inner topological configurations. Specifically, Gyroid and Diamond structures exhibit similar fatigue properties, whereas the IWP topology demonstrates superior fatigue performance^64^. In this study, we conducted a fundamental fatigue test analysis on the G_S hybrid structure and its individual components (i.e., G and S lattice structures) to evaluate their fatigue performance. Cyclic strain accumulation led to a gradual reduction in height of the specimen throughout the fatigue test, following two distinct phases (Fig. 4f). The first phase, spanning 0–10^5^ cycles, was characterized by rapid strain accumulation, whereas in the second phase the strain rate stabilized, indicating a slower deformation accumulation. The stress-strain curves from two repeated experiments at 0.7 ${\sigma }_{y}$, which exhibits close alignment, demonstrating high experimental reproducibility.

At a lower stress level of 0.6${\sigma }_{y}$, the strain-accumulation trends among the three structures remained similar (Fig. 4f). However, as the applied stress increased from 0. 6${\sigma }_{y}$ to 0. 7${\sigma }_{y}$, the strain rate in the second stage rose significantly, and distinct differences emerged in the fatigue behavior of the structures. This indicated that fatigue response variations become apparent at 0.7${\sigma }_{y}$ stress, making further increases in the applied stress unnecessary for this study.

Under 0.6${\sigma }_{y}$, none of the three structures experienced a 50% stiffness loss even after 10^6^ loading cycles, indicating good fatigue resistance at moderate stress levels (Fig. 4g). While these findings share a similar trend with earlier studies^65,66^, differences exist due to variations in the materials used. However, at 0.7${\sigma }_{y}$, the G structure reached 50% stiffness reduction between 2 × 10^5^ and 3 × 10^5^ cycles, demonstrating a significant decline in cyclic loading resistance. In contrast, the S structure exhibited much lower sensitivity to stress increments. In one repeated test, it reached 50% stiffness decay at 8.79 × 10^5^ cycles, while in another, it did not reach this threshold even after 10^6^ cycles, suggesting that its failure threshold may be close to 10^6^ loading cycles. Interestingly, the G_S hybrid structure demonstrated a fatigue performance comparable to that of the superior-performing S structure (Fig. 4g). Its stiffness degradation behavior closely followed that of the S component rather than that of the less fatigue-resistant G structure. This suggests that the hybrid structure can retrain and integrate the enhanced fatigue resistance of its constituent materials. The connection strategy proposed in this study plays a crucial role in enabling this integration, ensuring that the hybrid structure efficiently inherits the advantageous fatigue characteristics of its components.

### Performance comparison

As shown in Figs. 4h–j, the performance ratio, defined as the performance value of the hybrid structure divided by the average performance of its corresponding constituent single structures, indicates that existing strut-strut hybrid structures generally fail to achieve a strongly synergistic enhancement in both load-bearing capacity and damage resistance (or energy absorption). This indicates that although improvements can be achieved in one performance metric, they are typically accompanied by trade-offs that limit other properties.

TPMS-TPMS hybrid structures exhibit two distinct behaviors. One group further amplifies the aforementioned imbalance. For example, the hybrid structures represented by the golden yellow and cyan blue symbols achieve high energy absorption but relatively low compressive stiffness (Fig. 4i). The second group (from ref. ^3^) exhibits a more balanced mechanical response, although the improvement in some properties remains modest (Fig. 4i). For interpenetrating structures, most of their performance ratios fall below 1 (Fig. 4j).

In contrast, the compressive performance ratios of the strut-TPMS hybrid structures generated using the proposed method (red curve) are consistently greater than 1, indicating that the hybrid structures outperform their constituent architectures on average. This demonstrates that the proposed hybridization strategy enables a more effective balance between multiple mechanical properties, resulting in simultaneous improvements in structural stiffness, strength, fracture strain, and energy absorption capacity. Although the flexural stiffness is slightly below the reference value 1, the specimens used in the bending experiments were unoptimized structures representing only a simple combination of the constituent architectures (Supplementary Fig. 5h).

### Application demonstration

**Fig. 5:**
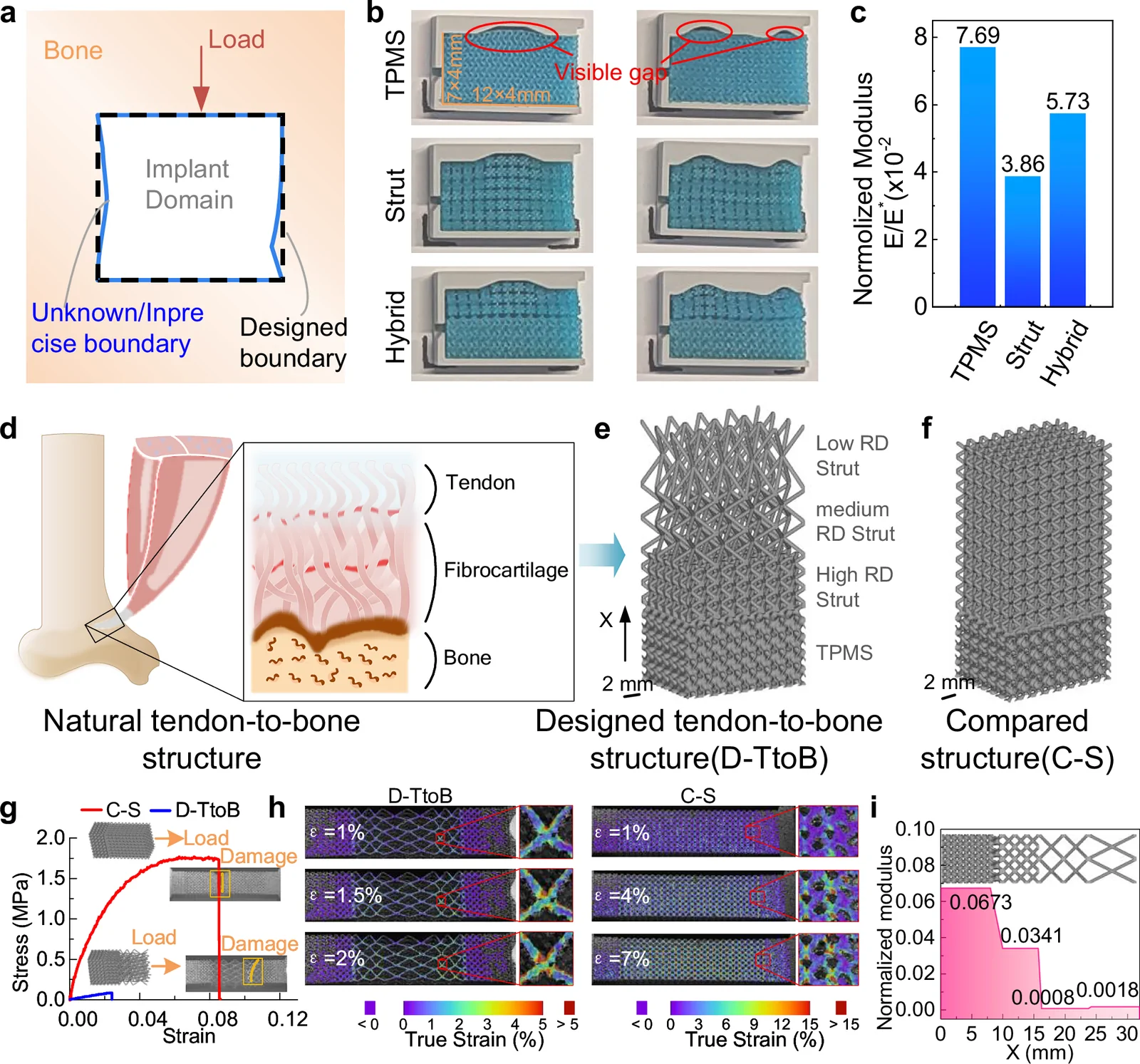
Design and experimental validation of conformal and biomimetic tendon-to-bone connection structures. **a** Design requirements for conformal structure. When the precise geometry of the mounting site is difficult to define (blue line), the structure must accommodate shape uncertainty. **b** Experimental comparison of conformability hybrid and single-structure designs. The TPMS structure alone (top) fails to conform to the target geometry, leaving a visible gap (highlighted by a red circle). **c** Comparison of normalized modulus of hybrid and single structures (simulation results). **d** Schematic drawing of the natural tendon-to-bone structure^86,87^. **e** CAD model of the designed biomimetic tendon-to-bone structure (D-TtoB) with gradient strut-based structure. **f** CAD model of G-BCC structure used for comparison (C-S). **g** Overall tensile stress-strain curves and corresponding damage screenshots at failure for the D-TtoB and C-S structures. **h** Strain distribution maps obtained from digital image correlation at various strain levels prior to failure. For the D-TtoB structure the full-field distribution is shown with a magnified view of the gradient strut-based junction on the right. For the C-S structure, the full-field distribution is shown with a magnified view of the TPMS-to-BCC transition interface on the right. **i** Partitioned normalized modulus of the biomimetic tendon-to-bone structure. Source data are provided as a Source Data file.

When TPMS lattice structures are selected for implants or other applications due to their exceptional mechanical properties, a key challenge arises: precisely defining the mounting site geometry can be difficult. This makes it challenging to design structures that accurately conform to the intended (Fig. 5a). To address this, we propose incorporating easily deformable strut-based structures along the edges of the TPMS sheet lattice. This design allows for shape adaptation even when only an approximate geometry of the mounting location was available.

To validate the shape-fitting capability of this hybrid design, we conducted a straightforward experiment. The structure was initially designed with a rectangular geometry to approximate the target region, given the difficulty in obtaining its precise shape (Fig. 5b). In the experiment, only half of the structure was fabricated, with the unconnected side of the G structure serving as the center of the actual structure. When assembled, the G structure alone failed to conform to the target geometry, leaving a visible gap (highlighted in red circle in Fig. 5b). In contrast, the hybrid structure, featuring edge struts, successfully conformed to the desired shape, achieving a fit comparable to that of a purely strut-based structure while significantly reducing the gap. Mechanical analysis further supported this approach. Finite element simulations showed that the hybrid design maintained the mechanical advantages of the G structure while enhancing its conformability (Fig. 5c). Furthermore, as demonstrated in Supplementary Movie 3, assembling the G structure alone into the target zone required significant force due to its low edge deformability, making installation challenging. The hybrid design mitigated these difficulties, providing a more adaptable solution.

The bone-tendon-muscle tissue unit is essential for the musculoskeletal system and human mobility, where musculoskeletal disorders rank among the leading causes of disability worldwide^44^. Lattice structures used as implants for tendon-to-bone repair must satisfy critical biomechanical requirements, particularly in matching the geometric and mechanical properties of natural tissues. One major challenge raised from the significant differences in geometry between bone and tendon is the requirement for implants to accommodate both structures simultaneously. Bone consists of a dense outer cortical shell surrounding a porous trabecular core (Fig. 5d)^67^, while tendons are layered and exhibit a fully anisotropic microarchitecture due to the alignment of fibrous tissue^44^.

A second challenge is the large modulus gradient at the bone-tendon interface. The tensile strength and modulus of cortical bone are 147 MPa and 15.477 GPa, respectively^68^, whereas the tendon has a tensile strength ranging between 4.1 and 22.1 MPa and a modulus between 217.7 and 592.4 MPa^69,70^. The stark contrast in these mechanical properties necessitates a gradual transition in structural stiffness to avoid stress concentrations and premature failure. Furthermore, it has been demonstrated in ref. ^71^ that elongated BCC lattice structures hold promise in meeting the anisotropic mechanical stiffness requirements of tendons, as their normal modulus significantly exceeds their shear modulus^72^.

To address both challenges, we developed a biomimetic hybrid structure by integrating G and B structures (and their variants) (Fig. 5e). This structure comprised four distinct regions, designed to mimic the natural bone-fibrocartilage-tendon formation, to address the geometric matching challenge. To further optimize mechanical performance, the structure was symmetrized, mitigating excessive mechanical gradient variation. Moreover, to ensure the high mechanical strength in high-stress regions, we designed a G_B hybrid structure with enhanced mechanical properties (Fig. 5f).

Mechanical testing of this structure (repeat curves see Supplementary Fig. 11) yielded critical insights into its damage behavior. The gradient-strut hybrid structure exhibited early failure, particularly at the inclined damage zone within the strut region (Fig. 5g). The high-relative-density regions of the G and B structures remained largely undeformed, indicating that damage was concentrated in the low-relative-density strut-based section, where structural strength is lower. In contrast, a structure composed of a homogeneous mixture of these two architectures demonstrated significantly greater load-bearing capacity, with damage localizing centrally in the B structure (Fig. 5h). This suggests that the hybridized design effectively distributed mechanical load across the entire structure, improving structural integrity. Strain distribution analysis (Fig. 5h and Supplementary Movie 4) further confirms these findings. Pre-damage strain distribution revealed that high strain concentration occurred at “×”-shape intersections rather than at the heterogeneous junctions between the G and B structures. A zoomed-in view of the G_B interface (highlighted in Fig. 5h) shows predominantly blue-colored regions, indicating a well-connected interface with minimal localized strain concentrations. Moreover, throughout the tensile testing, strain distribution remained relatively uniform, with no evidence of large, localized strain bands, further confirming the structural stability of the interface.

Finally, Fig. 5i illustrates the gradual decrease in the normalized modulus from the G structure to the BCC structure, effectively replicating the modulus gradient found in natural bone-tendon transitions. To accommodate tendon-like properties, the low-density BCC structure was elongated, enhancing its tensile stiffness compared to a standard BCC structure of equivalent relative density (Fig. 5i). Numerically, the maximum normalized modulus of the structure was 0.0673, which is 37–84 times greater than that of the terminal tendon-like region, including the lowest modulus at its connection positions (0.0008–0.0018). This modulus gradient closely aligns with the natural variation observed in bone-tendon transitions, ranging from 26 to 71 times, demonstrating the potential of this hybrid design for biomedical applications.

In summary, we propose a design strategy to achieve hybrid connections between strut-based and TPMS sheet structures via twinned unit cells. This unique approach establishes seamless transition structures by identifying TPMS skeletal geometries that share structural similarities with strut-based architectures. As a result, our method enables the hybridization of strut-based lattices, which lack continuous mathematical descriptions, with TPMS sheet structures across various configurations.

We also introduced voxel-based erosion and dilation techniques, enabling precise stiffness modulation and accurate performance prediction. The hybrid load-bearing conformal structures designed in this study integrated strut-based elements, providing enhanced adaptability to uncertain or imprecise geometrical boundaries while preserving favorable mechanical properties.

Furthermore, we present a biomimetic bone-to-tendon connection structure, featuring a geometrically variable design that gradually transitions from a porous, bone-like architecture to a fibrous, tendon-like structure within a single continuous volume of interest. This design achieved a 37- to 84-fold variation in structural stiffness, accommodating both geometric and mechanical property gradients necessary for artificial bone-to-tendon implants.

Our findings validate the potential of hybrid porous lattice structures, particularly in biomedical implants^73,74^ and structural engineering^75,76^, where precise mechanical tuning and geometric adaptability are crucial. The proposed approach offers a scalable and efficient solution for designing multifunctional architected materials.

## Methods

This study aims to develop and validate a generation method for hybrid lattice structures that can seamlessly integrate sheet-based TPMS and strut-based lattice architectures. To systematically validate the method, we designed a controlled comparative study encompassing three sheet-based TPMS structures (Gyroid (G), Diamond (D), Primitive (P)) and three strut-based lattice structures (Body-Centered Cubic (B), Face-Centered Cubic (F), Simple Cubic (S)). Based on these, five hybrid structures (G_B, G_F, G_S, D_B, P_B) were constructed. For example, the G_B hybrid structure represents the connection between a gyroid and a body-centered cubic lattice.

### Hybrid structure design and generation methodology

**Fig. 6:**
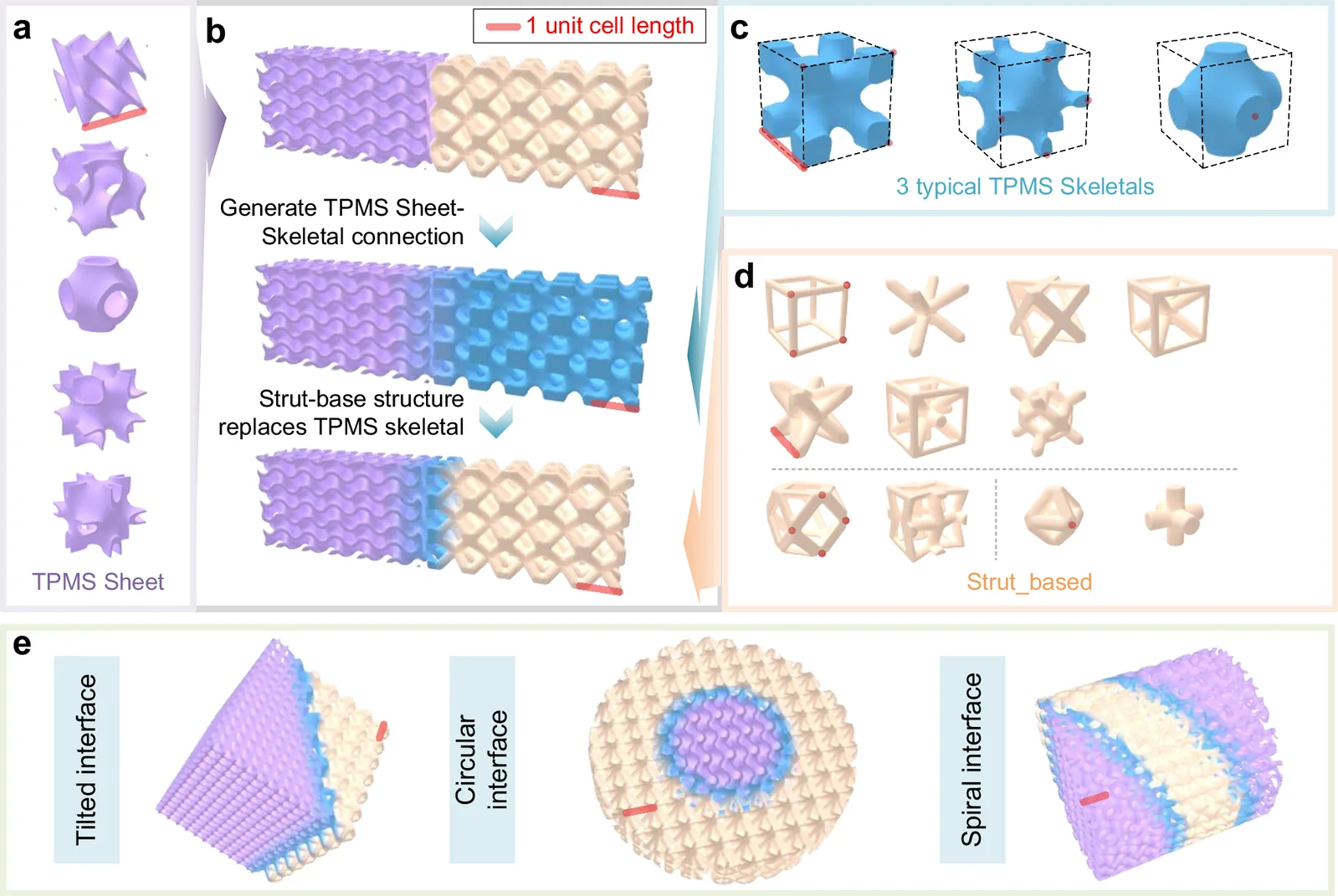
Schematic overview of connection strategies for hybridizing strut-based and TPMS-based lattice structures. **a** Representative TPMS-based structures (sheet configurations), including Gyroid (G), Diamond (D), Primitive (P) topologies, and so on, which are commonly used as continuous sheet architectures. **b** Evolution of hybrid lattice structure connection strategies, purple indicates TPMS sheet regions, yellow indicates strut-based regions, blue indicates TPMS skeletal regions, and the gradient-colored region indicates the transition structure. **c** Skeletal TPMS structures (I-Wrapped Package, Neovius, and Primitive) for approximating strut-based lattices. **d** Representative strut-based structures, including BCC, FCC, and so on. **e** Schematic drawing of the hybrid structure of several different connection interfaces using the proposed strategy. The red dots in (**c**, **d**) indicate the nodal positions of the structure, highlighting the distribution of boundary nodes. The red line segment denotes the length of a single unit cell, and this annotation applies to all subfigures.

Integrating TPMS sheet-based (Fig. 6a) and strut-based lattices presents a significant challenge due to their inherently different structural configurations. Achieving a continuous, smooth, and mechanically robust transition between these architectures is crucial to prevent geometric discontinuities (Fig. 6b, first row).

To address this, we developed a systematic connection strategy, which consisted of the following steps: (i) twinned unit cell approach, in which we identified skeletal structures with nodal configurations resembling the target strut-based lattices as twin unit cells (Fig. 6b, second row). (ii) transition structure formation, in which we used a closed-loop mathematical expression to define both the TPMS sheet structures and their corresponding TPMS skeletal structures (Fig. 6c) and generated the transition structure by weight value introduced in the two TPMS structures’ mathematical function. (iii) automated generation of the final hybrid structure, in which we retained the transition structure and replaced the skeletal structures with the target strut-based structure (Fig. 6b, third row) in non-transitional areas.

#### Mathematical formulation of twined unit cells

Analysis of strut-based unit cells revealed three primary nodal configurations: nodes at the eight cube vertices, the midpoints of the prongs, and the face centroids (Fig. 6d). These configurations corresponded to three TPMS skeletal structures, namely, I-Wrapped Package, Neovius, and Primitive, which we selected as twinned unit cells (Fig. 6c). Their mathematical representations are as follows:

1 $${{\varnothing}_{I-WP}}=\left(\cos \left(2ax\right)+\cos \left(2ay\right)+\cos \left(2az\right)\right)-2\\ \left(\cos \left({ax}\right)\cos \left(ay\right)+\cos \left(ay\right)\cos \left({az}\right)+\cos \left(az\right)\cos \left({ax}\right)\right)$$

2 $${{\varnothing}_{Neovius}}=4\cos \left({ax}\right)\cos \left(ay\right)\cos \left({az}\right)+3\left(\cos \left(ax\right)+\cos \left(ay\right)+\cos \left(az\right)\right)$$

3 $${\varnothing}_{{Primitive}}=\cos \left({ax}\right)+\cos \left(ay\right)+\cos \left({az}\right)$$

where x, y, z represent the Cartesian coordinates, and a is a parameter that controls the structural periodicity.

#### Transition strategy

To ensure a smooth transition between the TPMS sheet and skeletal structures, we explored two main approaches. (i) level-set-based transition, in which the TPMS skeletal structure function was superimposed with a weight parameter, defining a continuous level-set function for the transition region. (ii) weighted function overall with Boolean subtraction (or selected approach), in which two distinct TPMS skeletal structure functions were directly overlaid using weight parameters. Subsequently, Boolean subtraction was applied to remove a lower-density skeletal structure from localized regions, thereby transforming it into a sheet-like structure. This method was chosen for its ease of maintaining connectivity. Using this approach, the function governing the hybrid transition was expressed as:

4 $${\varnothing }={\alpha }_{1}{\varnothing}_{1}+{\alpha }_{2}{\varnothing }_{2}+...+{\alpha }_{n}{\varnothing }_{n}$$

where ${\phi }_{i}$ represents the functions of different structures and ${\alpha }_{i}$ are spatially-dependent weight parameters that define a continuous transition between structures. The weight parameters, ${\alpha }_{i}$, were determined using a sigmoid transition function^38,42^:

5 $${\alpha }_{i}=\frac{{e}^{-k{W}_{i}\left(x,y,z\right)}}{{\sum }_{j}^{n}{e}^{-k{W}_{j}\left(x,y,z\right)}}$$

where i=1~n, k is a constant to control the transition interval, and ${W}_{j}\left(x,y,z\right)$ represents the boundary function, defined by a point cloud ${P}_{j}\left({x}_{j},{y}_{j},{z}_{j}\right)$:

6 $${W}_{j}\left(x,y,z\right)={\left(x-{x}_{j}\right)}^{2}{+{\left(y-{y}_{j}\right)}^{2}+\left(z-{z}_{j}\right)}^{2}$$

At this stage, the transition phase was established (Fig. 6b, second row). It should be noted that at the macroscopic scale, an excessively large transition region is undesirable, as it would reduce the proportion of each constituent architecture and diminish the value of the hybrid design, particularly given the fact the available number of transitory unit cells is often limited by the overall size of the construct and the resolution of the 3D printing technique. This consideration is particularly relevant in applications such as bone‑to‑tendon interfaces and integrated load‑bearing‑conformal systems. Therefore, in this study, we constrain the transition length to one unit cell size to create twinned unit cells (Supplementary Fig. 13b–e). For applications requiring extended transition zones, the analytical framework provided (Supplementary Note 10 and Supplementary Fig. 13) can be referenced for tailored adjustment.

#### Finalizing the hybrid structure

Once the transition structure was formed, the TPMS skeletal structure was selectively replaced to complete the hybrid lattice design. This was achieved by first verifying the spatial point cloud of a unit cell to determine if it lies within the boundary of the strut-based structure, and, second, by constructing the designated strut-based structure using the voxel approach, utilizing a predefined structural framework file^58^.

The finalized hybrid structure is presented in Fig. 6b, third row, demonstrating a fully connected, mechanically optimized architecture. This methodology is highly adaptable and can be extended to a broad range of interface configurations, as in Fig. 6e.

Automated hybrid structure generation for 3D printing and FEM simulation. Traditional single-lattice structures are typically modeled using repetitive unit cell arrays, created in common commercial modeling software, such as SolidWorks, before being exported in the.STL format file for 3D printing. However, the hybrid structure developed in this study require a non-uniform arrangement of unit cells, necessitating an individualized modeling approach. This poses a complex and time-intensive challenge when using conventional modeling software.

To address this, we introduce a design-domain description approach that enables the automated generation of hybrid structures. A MATLAB script (version 2020a) was developed for this purpose, and the full code is provided as Supplementary Code 1. Detailed instructions for using the code are included in the accompanying README file. As illustrated in Supplementary Fig. 14, the workflow automatically generates voxel matrices representing the hybrid structures, which can be converted into.INP^77^ and.STL^78^ files. This framework enables the efficient fabrication of hybrid structures while ensuring compatibility with both 3D printing technologies and finite element method (FEM) simulations.

The primary algorithmic inputs and controlled variables are as follows. (i) Design domain: The spatial regions corresponding to each substructure are defined using STL files (see Supplementary Note 9 and Supplementary Fig. 12 for details). (ii) Key parameters: The user specifies the number of design domains (*n*_d_, representing the number of substructures to be combined), the number of voxels per unit‑cell edge (*n*_v,_ defining the resolution), the voxel size (*L*_v_, the voxel edge length determined by the unit cell size and *n*_v_), and the file number *n*_*in*_ (corresponding to an STL‑format file that defines the structural boundary, i.e., the design domain). These STL files must follow a prescribed naming convention and be stored in the program’s working directory. After the number of design domains is entered, a second input window appears with corresponding fields for each substructure. In this window, the user specifies the structure name (*Name*), the relative density (*RD*_*i*_), and the structure type (*Type*: sheet or strut). The parameter *n*_v_ is an integer, typically chosen as a multiple of 10. Larger values provide higher geometric resolution but increase computational cost (see Supplementary Fig. 13a for a sensitivity analysis). The unit cell edge length is defined by the product *n*_v_ × *L*_v_. Relative density is assigned independently for each constituent structure, requiring *n*_d_ separate relative density values.

The design-domain geometries (in.STL format) are discretized into voxels. Each voxel is then evaluated according to structural inclusion criteria. Voxels satisfying these criteria, based on the TPMS implicit function and nodal information describing strut structures, were retained and represented as a logical matrix. This matrix was subsequently used to construct the final printable output model in.STL format. A schematic overview of the entire process is shown in Supplementary Fig. 14. For structures intended for finite element simulation, the logical matrices were converted into.INP files, with mesh information formatted according to previously reported methods^77^.

The relative density control and compensation mechanism operates as follows. First, relative density is defined only for the constituent structures. Once the relative density of each constituent structure is specified, transition cells are automatically generated based on neighboring structures to ensure smooth connectivity. This process uses the target relative density of each constituent unit to determine the corresponding wall thickness (for TPMS structures) or strut diameter (for strut-based structures). These derived dimensions are then used as the target wall thickness or strut diameter for the transition cells. As a result, deviations in the relative density of transition cells may occur (e.g., Fig. 2c). However, the trend of variation remains close to the maximum values in neighboring regions and can be adjusted using the proposed voxel-erosion method.

Compensation was implemented in two steps. First, systematic errors inherent to the MATLAB algorithm used to generate printable files were corrected by analyzing topology-specific deviations. For example, if a target relative density of 0.30 produced an actual density of 0.29, a compensation factor *P*_com_ = 0.30/0.29 was applied, and the corrected target value became 0.30 × *P*_com_ (see Supplementary Fig. 13f for details). For the G, D, P, B, S, and F topologies fabricated in this study, the compensation factors were 0.923, 0.860, 0.917, 0.950, 0.967, and 0.940, respectively.

Second, a methodological compensation step was implemented based on the analysis presented in Fig. 4d, e, which relates the number of eroded or dilated voxels in transition unit cells to the relative density of the interval. Supplementary Fig. 9 illustrates the adjusted relative density distribution across the transition cells. In practice, the target relative density can be approached iteratively via controlled voxel erosion or dilation. According to the analysis shown in Supplementary Fig. 13f, the residual relative density error after the first compensation step is already within an acceptable range. Therefore, when strict accuracy in the relative density of transition unit cells is not required, the second compensation step may be omitted.

In addition, variations in the transition interval length were analyzed (Supplementary Fig. 13). Based on these results, the *k*-value used in this study ranged from 0.01 to 0.2, depending on the structural dimensions, ensuring that the transition region remained approximately at the scale of a single unit cell. The fabricated structures, therefore, closely matched the intended design specifications.

Running Supplementary Code 1 on a workstation equipped with an Intel Xeon Platinum 8175 M processor (2.5 GHz) and 192 GB of RAM generates a hybrid structure consisting of 7 × 7 × 7 unit cells in approximately 33 min. The relatively long runtime is due to the voxel-based modeling approach, which produces STL models with redundant triangular facets. Although this represents a current limitation and an opportunity for future optimization, the method nevertheless eliminates the impracticality of manually constructing such complex hybrid lattice models.

### Fabrication of structures

The compression test specimens consisted of three TPMS sheet structures, three strut-based structures, and five hybrid structures, all designed as 28 × 28 × 28 mm³ cubic lattices, each comprising 7 × 7 × 7 unit cells (with individual unit cells of 4 × 4 × 4 mm³ in size) and a relative density of 30%. The G, S, and G_S structures used in fatigue testing followed the same dimensional parameters.

For the fabrication of specimens intended for tension, bending, torsion, and combined tension-torsion and compression-torsion tests, the G_S structure and its constituent substructures G and S were selected. All the specimens were fabricated with a unit cell size of 4 × 4 × 4 mm³ and a relative density of 30%. Corresponding clamping end structures were designed for each specimen in accordance with the experimental requirements.

For the tendon-to-bone connection structure, the G and high-relative-density B structures had 2 × 2 × 2 mm³ unit cells with a relative density of 25%. The medium-relative-density B structure had 4 × 4 × 4 mm³ unit cells, while the low-relative-density B structure used 4 × 8 × 4 mm³ unit cells, with relative densities automatically adjusted to maintain uniform strut diameters.

All the specimens were fabricated using Polyjet technology with a J5 MediJet™ 3D Printer (Stratasys^®^ Ltd., USA). The primary material was VeroCyan™ (Stratasys^®^ Ltd., USA), while WSS™ 150 (Stratasys^®^ Ltd., USA), a water-soluble support material, was used to facilitate post-processing.

For the load-bearing-conformable structures in the application case study, models were designed with dimensions of 7 × 12 × 3 unit cells (each 4 × 4 × 4 mm³) and a relative density of 25%. These structures were printed using soft materials on J5 MediJet™ 3D Printer. The materials used were a combination of Elastico™ and VeroCyan™, set to Digital Materials, with an A-shore hardness of 85, and the support material remained WSS™150. After printing, all the aforementioned specimens were submerged in a beaker filled with tap water for 20–30 h. The water was changed multiple times during this period until the support material dissolved. To mitigate the influence of printing direction, all specimens were printed along the intended loading direction. To assess reproducibility, three duplicates of all specimens utilized in the mechanical experiments were fabricated.

### Morphological characterization

To evaluate the print quality, we measured the overall dimensions of the specimens using a vernier caliper (accuracy = 0.01 mm), weighed specimens using a precision scale (accuracy = 0.0001 g), and calculated the actual relative density by dividing the measured weight of the specimens by their apparent volume, then further dividing it by the density of the base material. µCT scans were conducted on five hybrid structures to assess internal print quality and generate 3D reconstruction models. The scanning was performed using the TESCAN CoreTOM system (Germany) at 100 kV voltage and 300 µA current. Voxel sizes of 30 × 30 × 30 μm^3^ were utilized. Following the reconstruction of 2D images with built-in software, we analyzed the scans using Fiji^79^ with the ImageJ plugin (ver.2.16.0) to compute layer-by-layer relative densities. Finally, the data were processed using the 3D Viewer plug-in to generate 3D reconstruction models of the structures.

### Mechanical testing

Quasi-static uniaxial compression tests were conducted using an ElectroPuls™ E10000 (INSTRON, USA) machine equipped with a 10 kN load cell. The tests were performed using a compression speed of 2 mm per min until either failure or 60% strain was reached. To monitor local deformations and to measure full-field strain fields, digital image correlation (DIC) analysis was performed using a Q-400 2 × 12 MPixel 3D DIC system (LIMESS GmbH, Germany) at a recording frequency of 1 Hz. The DIC analysis software (Instron 4D v4.6, Dantec Dynamics A/S, Denmark) was used to extract true principal strain data. To facilitate DIC tracking, specimens were first coated with a white background, followed by the application of a black speckle pattern.

Pure torsion and biaxial loading (i.e., Tension-torsion and compression-torsion loading) tests were performed using an INSTRON ElectroPuls™ machine (Instron Systems, Norwood, United States) with a 10 kN load cell. Torsion tests were conducted at 11.5 rad per min to 120 degrees or until it broke. For biaxial loading, the same torsion speed was used with a tension/compression rate of 2 mm per min.

For tensile specimens and tendon-to-bone connection specimens, uniaxial tensile tests were conducted using a Lloyd Instruments LR5K machine (USA) with either a 500 N or 5 kN load cell, depending on the structural design. DIC strain measurements were taken separately for each of the four structural regions. The G and high-density BCC segments exhibited negligible strain prior to failure, making modulus calculations impractical. Instead, strain data were extracted from the corresponding section of the comparison structure.

Numerous studies^29,65,80^ have investigated the fatigue response of TPMS porous structures, including topologies such as G, D, and P, revealing distinct fatigue behaviors across different architectures. Based on the fatigue responses reported for these TPMS structures, we conclude that fatigue tests are typically conducted with failure criteria defined as 10⁶ cycles, a specified level of stiffness loss, or structural fracture. The applied cyclic loads in these tests generally range from 20 to 80% of the yield strength, with a majority concentrated between 50 and 80%. Therefore, fatigue testing was performed on the G_S hybrid structure and its corresponding substructures, given its superior mechanical performance. The same INSTRON ElectroPuls™ E10000 machine (10 kN load cell) was used, employing sinusoidal waveforms at a loading frequency of 5 Hz and a stress ratio (R) of 0.1. The initial fatigue stress was set to ${\sigma }_{\max }{=0.6\sigma }_{y}$ (where ${\sigma }_{y}$ represents the yield stress from quasi-static uniaxial compression tests). The fatigue test terminated when either 50% stiffness reduction (i.e., when strain reached twice the initial value) or 10^6^ loading cycles were completed. If no significant differences were observed, the stress level was increased incrementally by 0.1${\sigma }_{y}$ until differentiation was evident, at which point an additional repeat experiment was conducted.

The stress-strain curves were generated from the force-displacement data, and the modulus of elasticity was determined as the slope of the linear region (30–70% strain). Yield strength was defined as the stress at the intersection of the stress–strain curve with a 0.2% offset line.

### FEM simulation

FEM simulations were conducted to analyze linear elastic deformation, compute stress-strain distributions, and determine elastic moduli across various relative densities. Abaqus/Explicit 2021 (Dassault Systèmes Simulia Corp, France) was used for all the simulations. Supplementary Code 1 was running in MATLAB 2020a (MathWorks Inc., USA) to generate the input (.INP) files^77^. Each INP file contained meshing details and used C3D8R elements. Relevant analysis has been performed (Supplementary Note 11); the hourglassing effect was found to be minimal and can be neglected (see Supplementary Fig. 15b for more details). for all the models. Manual modifications were made to: 1. Incorporate two rigid flat plates as test indenter simulations, 2. Define contact conditions between the lattice structure and the flat plates (friction contact = 0.1, normal hard contact), 3. Set loading speed to 1000 mm per second (Relevant analysis confirms that quasi-static conditions were satisfied. Please refer to Supplementary Fig. 15a for details.) 4. Assign material properties (modulus: 1569.41 MPa, Poisson’s ratio: 0.4) and 5. Employ an explicit dynamic analysis approach.

### Statistical analysis

Statistical analysis was performed using Origin 2021 (OriginLab Corporation, USA). Unless otherwise stated, data were analyzed using one-way ANOVA, followed by Tukey’s multiple comparisons test. Statistical significance was set at p<0.05.

## Supplementary information

Supplementary Information

Description of Additional Supplementary Files

Supplementary Movie 1

Supplementary Movie 2

Supplementary Movie 3

Supplementary Movie 4

Supplementary Movie 5

Supplementary Movie 6

Supplementary Movie 7

Supplementary Code 1

Transparent Peer Review file

## Source data

Source Data

## Data Availability

All raw data necessary to reproduce the results are available Supplementary Information and Source Data file. Source data are provided as a Source Data file, and is available at the following Figshare repository: https://doi.org/10.6084/m9.figshare.33000608. Source data are provided with this paper.

## Code availability

Codes are provided as Supplementary Code 1.
