# Supplementary material for: Strategy for hybrid connection of porous strut and triply periodic minimal surface lattice structures
Source: Nat Commun. 2026 Oct 5;17:9699. doi: 10.1038/s41467-026-77560-7 (PMC13639051; doi:10.1038/s41467-026-77560-7)
Supplement: Supplementary file 1 [file 41467_2026_77560_MOESM11_ESM.pdf]

nature portfolio

Peer Review File

Strategy for hybrid connection of porous strut and triply periodic minimal surface lattice structures

Corresponding Author: Dr Jianxing Yang

Parts of this Peer Review File have been redacted as indicated to remove third-party material.

This file contains all reviewer reports in order by version, followed by all author rebuttals in order by version.

Version 0:

Reviewer comments:

Reviewer #1

(Remarks to the Author)
The contribution proposes a strategy for the development of strut-TPMS hybrid metamaterials. Different combinations of strut and TPMS designs are investigated, including gyroid, diamond, BCC and FCC structures. Significant mechanical performance tunabilities are reported, while insights into the fatigue performance are provided. Moreover, example applications in tendon-bone interface applications are given, reporting designs of biomechanical relevance. The work is overall of interest and well structured. There are, however, important analysis and methodology issues that need to be addressed before further consideration:
1) The novelty of the formulation with respect to existing linear hybridization patterns needs to be better described. How do twinned unit cells differ from mere linear grading?
2) Relevant to the previous question: What is the value of the transition parameter k selected? How has its value been determined?
3) In the finite element modeling simulations, it is unclear why dynamic explicit and not an implicit static analysis has been performed. The deformation rate applied does not match the static experiments performed. Relevant clarifications need to be provided.
4) In the results of Figure 3, nearly hybridization combinations lead to brittle hybrid designs out of combinations of brittle and ductile constituents. What is the mechanism driving the overall mechanical performance? While gains in the yield strength for certain designs are reported, the overall energy absorption appears to be negatively impacted. What could be potential mitigation strategies?
5) The development of biomimetic, lattice-based, tendon and ligament metamaterial designs has been explored in the literature. Relevant literature mechanical insights are however missing.
6) The same applies to the fatigue performance of TPMS-based architected cellular materials, where, inner topology-dependent fatigue mechanics have been extensively characterized. The study is missing a proper acknowledgement of existing contributions.
7) A description of the limitations of the current approach along with the need for further research perspectives is missing from the analysis.
8) The presentation of Figure 4 c-e needs to be enhanced. In its current state, the relevant analysis hardly interpretable.
9) The language of the manuscript needs to be thoroughly proof-read and different typos and grammatical inconsistencies appearing at various positions have to be corrected.

Reviewer #2

(Remarks to the Author)
Summary

The manuscript presents a computational design workflow that combines closed-form triply periodic minimal surface (TPMS) sheet representations with strut-based lattices via a “twinned unit cell” transition strategy to realize hybrid, conformal load-bearing structures. While related graded and multi-architecture approaches exist, the claimed novelty is an automated, geometry-aware transition that preserves connectivity between sheet- and strut-based lattices and, together with voxel

“corrosion/expansion,” aims to enable precise stiffness modulation and performance prediction. The 3D printing of high-performance, functionally distinct architected materials remains an important topic, and this manuscript demonstrates hybrid conformal structures and a biomimetic bone-to-tendon junction within a single continuous volume. The pipeline’s compatibility with finite element modeling (FEM) and additive manufacturing, and the demonstration of programmable transitions across uncertain geometries, suggest potential applications in biomedical implants and structural engineering.

My main concern centers on novelty in light of prior strategies for graded lattices, multi-architecture blends (including sheet/TPMS blends), and topology-optimized interfaces, which already address many of the stated challenges (e.g., interfacial stress management, density control, and manufacturability). The present work appears to differ primarily in the specific “twinned unit cell” matching of nodal configurations and a voxel-based implementation, rather than introducing a fundamentally new interface paradigm. In addition, the scope appears quite narrow: the manuscript focuses on connecting two or more distinct lattice phases. For simple load cases, a single architecture with graded density or thickness often suffices; for complex, spatially varying loads, continuously changing architectures (within a single topology family or via smooth morphology transitions) may be more appropriate than discrete multi-phase hybrids. The manuscript does not clearly justify when and why discrete phase-to-phase connections are preferable, which limits perceived generality and raises questions about fit for a broad, general-interest journal.

Aside from concerns on novelty and scope, I offer the following major and minor critiques that would improve the quality of this manuscript. Note that some comments apply throughout and only call out a few example instances, with too many to cite them all.

Major comments

- The direct connection of strut-based and sheet (TPMS) lattices is a relatively niche use case with limited demonstrated usefulness and has been addressed previously. The manuscript asserts that this is an important scenario but does not substantiate why discrete phase-to-phase connections are needed. Overall, the contribution reads more like an engineering solution than a fundamental scientific advance.
- Many established approaches already address this problem, including those the authors cite (e.g., graded lattices and topology-optimized interfaces). The authors argue that existing solutions concentrate stresses in the boundary layer, yet the proposed method similarly focuses interface stresses within a single-cell-thick layer. This may be acceptable at structural scales, but for metamaterials—where the unit cell is small relative to the overall dimensions—such sharp transitions can be problematic and are often better handled via continuous gradients. As a result, the claim of a substantial gap in the literature is not convincing.
- The manuscript acknowledges that gradient-based designs are common and useful but states that “their application within single-architecture structures often results in porosity variations.” Please clarify: Why is this a problem? What are the specific exceptions to this claim (“often”), and what shortcomings do those exceptions have, if any?
- The statement that “excessive porosity adversely affects manufacturability” is not substantiated. If a process can fabricate both strut-based and sheet lattices, it should plausibly be able to fabricate interfaces with modestly different porosity distributions. Please provide evidence that directly tie porosity levels at the interface to manufacturability limits.
- The introduction states that a flaw of existing solutions is that the local relative density of the transition cell deviates from that of surrounding cells. However, this also appears to occur in your approach (e.g., Fig. 2c). Please reconcile this inconsistency.
- Computational analyses are not typically used to validate an approach, as stated at the end of the introduction. Please clarify and/or revise the methodology to follow standard practice: for example, use experiments to validate the modeling framework (including the transition strategy), and then use simulations to explore the parameter space within validated bounds.
- The load cases explored are too narrow. Hybrid lattices will experience a variety of loading modes and orientations. At a minimum, include tests and simulations in tension, bending, and torsion, and probe multiple load orientations relative to the interface. This broader evidence base is also necessary to justify the choice of two discrete architecture types versus a continuously varying architecture tailored to local stress states.
- To support the claim that the proposed method is significantly better than alternatives, provide direct, quantitative benchmarking against representative gradient-based and topology-optimized interface strategies (e.g., comparing stiffness, strength, fatigue resistance, interfacial stress distribution, and manufacturability metrics at matched relative density).
- The methods lack sufficient detail for generalization and reproducibility. Important steps appear to rely on manual “compensatory adjustments,” and code/parameters are not provided. Please specify the full pipeline, inputs, and decision rules, and share code or pseudocode to ensure reproducibility.
- Methodological rigor needs strengthening (in addition to the validation concern above). Please ensure clear problem statements, controlled comparisons, and complete reporting of assumptions, boundary conditions, and uncertainty.
- A core contribution appears to be the automation of the transition structures. The algorithmic details should be described in the main text, not only stated as “automated.” Please include the matching criteria, search/optimization strategy (if any), voxel operations (corrosion/expansion), parameter ranges, and computational cost. Also provide the limits of the generalizability.

Minor comments

- Video S1: Instead of “Speed is accelerated,” report the actual playback speed (e.g., “4× real time”) or the real-time duration and the acceleration factor.
- The manuscript uses vague phrasing such as “unique mechanical characteristics” and “distinct advantages and limitations.” Please specify what characteristics, advantages, and limitations you mean, and quantify where possible.
- The introduction is overly long and repetitive (roughly three pages). Please streamline to reach the central contribution

more quickly, reduce redundancy, and state precise research questions and advances.
- Fig. 3: The arrangement of panels a–c is confusing. Clarify the logical sequence, improve alignment, and explain the colored lines and backgrounds. The inset organization appears cluttered; consider reformatting or simplifying to improve readability.
- Ensure complete figure legends throughout (e.g., Fig. 5g appears to lack a legend). All symbols, colors, and abbreviations should be defined within each figure or caption.
- The current configuration of the simulation is not demonstrably quasi-static and lacks essential controls. At 1000 mm/s, the response is likely rate-influenced unless quasi-static conditions are verified. Explicit dynamics with reduced integration (C3D8R) can suffer from hourglassing and artificial compliance without appropriate controls. Linear elasticity and NLGEOM off omit buckling and large-deformation effects typical of lattices, so reported stiffness/curves may only be valid at very small strains. Friction significantly influences compression results and requires calibration or sensitivity analysis. Please verify quasi-static conditions (e.g., kinetic-to-internal energy ratio, mass scaling disclosure), use hourglass control and report hourglass energy fractions; consider element types less prone to hourglassing (e.g., C3D8I) or perform a mesh convergence study, and enable geometric nonlinearity where relevant and justify constitutive choices.
- Constituent structures are assigned relative densities, whereas transition structures are “generated automatically” without explicit target density, followed by unspecified “compensatory adjustments,” with varying transition lengths. The manuscript also claims that fabricated structures match designs without presenting evidence. Please formally define how transition voxels are assigned and how relative density is controlled across the interface; report as-designed and as-built densities; and clarify how consistency is enforced so that mechanical property comparisons and scaling (e.g., modulus vs. density) are meaningful.

Version 1:

Reviewer comments:

Reviewer #1

(Remarks to the Author)
The authors have addressed all issues raised, thoroughly revising the manuscript and enhancing its quality. The work advances the state of the art in hybrid connections of porous lattice structures.

(Remarks on code availability)

Reviewer #2

(Remarks to the Author)
The reviewer appreciates the extensive effort that went into revising this manuscript, which has improved the work. However, because some of the original comments were fundamental in nature and inherently difficult to resolve within the current framework, several of the responses feel superficial or hypothetical, leaving key issues unaddressed.

For example, while the authors acknowledge that their initial claims regarding novelty and breadth were overly broad, they now repeatedly point to "biological attachment interfaces (e.g., tendon-bone connections)" as a primary use case "for scenarios that require abrupt functional transitions within limited spatial regions." While this is a valid and useful application, narrowing the scope to this specific domain significantly limits the breadth and general readership of the work.

Ultimately, this limitation stems from the underlying nature of the framework; not from a lack of effort by the authors. The reviewer believes the authors have addressed the comments as much as practically possible given these constraints.

(Remarks on code availability)

Open Access This Peer Review File is licensed under a Creative Commons Attribution 4.0 International License, which permits use, sharing, adaptation, distribution and reproduction in any medium or format, as long as you give appropriate credit to the original author(s) and the source, provide a link to the Creative Commons license, and indicate if changes were made.
In cases where reviewers are anonymous, credit should be given to 'Anonymous Referee' and the source.
The images or other third party material in this Peer Review File are included in the article's Creative Commons license, unless indicated otherwise in a credit line to the material. If material is not included in the article's Creative Commons license and your intended use is not permitted by statutory regulation or exceeds the permitted use, you will need to obtain permission directly from the copyright holder.
To view a copy of this license, visit https://creativecommons.org/licenses/by/4.0/

We sincerely thank you and the reviewers for the careful evaluation of our manuscript entitled "Strategy for hybrid connection of porous lattice structures: the connection between strut- and triply periodic minimal surface-based structures" (ID: NCOMMS-25-52635). We appreciate the detailed and constructive comments, which have been extremely valuable in improving the clarity, rigor, and overall presentation of our work. In response to the reviewers' and editor's concerns, we have carefully revised the manuscript and performed additional analyses and clarifications where necessary. In particular, we have strengthened the discussion of the novelty and significance of the proposed design strategy, clarified the methodological framework, expanded the discussion of related literature, and improved the presentation of several figures and sections of the manuscript. The specific additions made for this revision are as follows:

- We have restated the core methodology with additional details, elaborating on the relative density control strategy within the hybrid approach, and provided corresponding figures in the Supplementary Material.
- We have consolidated the code used to generate the structures, written a read-me file, and made the complete code available as Supplementary Material.
- We have conducted additional uniaxial tests (tension, bending, torsion) and biaxial tests (tension-torsion, compression-torsion), and added corresponding experimental results, analysis text, and figures, along with related videos as Supplementary Materials.
- We have incorporated findings from existing studies on hybrid strategies and added comparative analysis figures and related discussion in the revised manuscript.
- We have restated the novelty and significance of the proposed design strategy, as well as the current state of the art, in the Introduction section.
- We have clarified the methodological framework and supplemented relevant literature discussions in Sections 2.2, 2.3, 2.4, 2.6, 4.1, and 4.4, and expanded the Conclusion with additional remarks on limitations and future work.
- We have added detailed descriptions of the finite element analysis in Section 4.5 and the Supplementary Material.
- We have included a performance analysis of hybrid structures based on the proposed method, which was previously lacking (presented in Section 2.2).
- We have corrected errors/typos in Figures 3, 4, and 5 and improved their layout and quality for better clarity.
- We have polished the language throughout the manuscript.

- We have made several minor adjustments not explicitly mentioned above to ensure overall coherence, including refinements to the text, revisions to figure and video annotations, and updates to figure numbering.

All reviewer comments have been addressed in detail in the point-by-point responses below. Corresponding revisions have been incorporated into the manuscript, and all changes are clearly indicated using colored text to facilitate evaluation.

We hope that the revisions satisfactorily address the reviewers' concerns and demonstrate the significance and robustness of the proposed approach. We are grateful for the opportunity to revise our work and would be pleased to provide any further clarification if needed.

Reviewer #1

Remark 1.1. The contribution proposes a strategy for the development of strut-TPMS hybrid metamaterials. Different combinations of strut and TPMS designs are investigated, including gyroid, diamond, BCC and FCC structures. Significant mechanical performance tunabilities are reported, while insights into the fatigue performance are provided. Moreover, example applications in tendon-bone interface applications are given, reporting designs of biomechanical relevance. The work is overall of interest and well structured. There are, however, important analysis and methodology issues that need to be addressed before further consideration.

We thank the reviewer for the positive assessment of our work and for recognizing the potential interest of the proposed strut-TPMS hybrid design strategy. We also appreciate the reviewer's constructive feedback regarding the analysis and methodology. These comments have been carefully considered and have helped guide several revisions and clarifications in the manuscript, which are addressed in detail in the responses below.

Remark 1.2. The novelty of the formulation with respect to existing linear hybridization patterns needs to be better described. How do twinned unit cells differ from mere linear grading?

We thank the reviewer for raising this important point. Conventional linear hybridization strategies typically rely on parameterized geometric variations (*e.g.*, changes in strut diameter, wall thickness, or unit cell scaling) to create gradual transitions within the same or closely related lattice topologies. When applied to fundamentally different topologies, such as strut-based and TPMS architectures, existing approaches often rely on geometric interpolation or distance-field blending, which may produce poorly controlled intermediate geometries and unpredictable mechanical properties.

In contrast, the twinned unit cell strategy proposed in this study establishes a transition by identifying structural correspondences between the nodal connectivity of strut lattices and the skeletal representations of TPMS. By selecting TPMS skeletal configurations whose nodal characteristics match those of the target strut architecture, the transition region can be constructed using structures that retain explicit mathematical representations. This enables more controllable, geometrically compatible transitions between otherwise incompatible topologies, avoiding the limitations of purely geometric interpolation approaches.

To clarify this distinction and better highlight the novelty of the proposed formulation, we have expanded the relevant discussion (in red text) in the Introduction of the revised manuscript as follows:

"To tailor the mechanical behavior of architected materials, researchers have widely explored functional gradient strategies, in which structural parameters vary gradually throughout the architecture. These gradients can be implemented through changes in strut diameter or surface thickness [20, 21], scaling of unit cells [22], or controlled deformation of unit cells through compression or elongation [23-25]. Such approaches typically generate transitions between identical or closely related architectures [26, 27]. Gradient strategies employing linear [28, 29], step-wise [30, 31], or exponential [32, 33] transitions have been shown to enhance the mechanical performance of architected structures in diverse applications [34], including improved energy absorption in crash-protection systems [31, 35] and the modulation of mechanical cues in bone tissue scaffolds [36, 37] to mitigate stress shielding and promote bone regeneration [38]. TPMS architectures are particularly well suited to such gradient designs because they are defined by continuous mathematical surfaces [39-43]. In contrast, strut-based lattices generally lack a continuous analytical description, which makes the realization of smooth geometric transitions considerably more challenging."

"To address these challenges, geometric interpolation approaches have been proposed to blend distinct lattice architectures [48, 49]. In these methods, distance-field-based weighting functions [50, 51] are typically used to generate intermediate geometries between two structures. Although such interpolation techniques expand the design space and enable spatially varying architectures [52-54], they suffer from important limitations. The geometry of the transitional region is often difficult to control, making the mechanical behavior of the resulting structure difficult to predict. Moreover, when the underlying topologies differ significantly, interpolation can generate irregular or extreme geometric features that compromise manufacturability and structural performance. Recent studies [55] have attempted to alleviate these issues by replacing direct interpolation with dual-architecture superposition strategies to construct transitional unit cells. Despite these advances, robust strategies for directly connecting fundamentally different lattice topologies remain challenging and largely unexplored."

Remark 1.3 Relevant to the previous question: What is the value of the transition parameter k selected? How has its value been determined?

We thank the reviewer for this important question. The transition parameter k controls the length of the transition region between the two connected lattice architectures. Its influence on geometric connectivity and transition interval size has been systematically analyzed in Figure S10 of the supplementary document and the associated discussion.

In the proposed twinned unit-cell framework, the transition region is intentionally designed to remain approximately one unit cell in length, ensuring geometric continuity and minimal distortion of the constituent architectures. The value of k is therefore selected according to the overall structural dimensions and the number of unit cells. When the number of unit cells and structural dimensions are fixed, decreasing k reduces the transition interval, whereas increasing the number of unit cells with a fixed k also results in a shorter normalized transition region. In practice, k is adjusted so that the transition interval remains close to the size of a single unit cell.

To clarify this design principle, we have expanded the parameter selection explanation in Section 4.1(in red text) of the revised manuscript.

> *"In addition, variations in the transition-interval length were analyzed (Figure S10 of the supplementary document). Based on these results, the k-value used in this study varied between 0.01 and 0.2 depending on the structural dimensions, ensuring that the transition region remained approximately at the scale of a single unit cell."*

Remark 1.4. In the finite element modeling simulations, it is unclear why dynamic explicit and not an implicit static analysis has been performed. The deformation rate applied does not match the static experiments performed. Relevant clarifications need to be provided.

We thank the reviewer for raising this important point regarding the finite element simulation methodology. The dynamic explicit solver was selected because it provides improved numerical stability and convergence when simulating the large deformations and complex contact conditions encountered during the compression of lattice structures. In our simulations, compression was applied through a displacement-controlled rigid plate, which closely replicates the boundary conditions used in the experiments.

To ensure that the simulations represent quasi-static conditions, we conducted an additional loading-rate sensitivity analysis. The results confirm that the selected deformation rate produces negligible inertial effects and satisfies quasi-static requirements. The corresponding analysis has now been included in Figure S12a of the supplementary document.

To clarify the modeling approach and the justification for using the dynamic explicit solver, we have expanded the description (in red text) in Section 4.5 of the revised manuscript as follows.

> *"Relevant analysis confirms that quasi-static conditions were satisfied. Please refer to Figure S12a of the supplementary document for details."*

And the corresponding analysis in Figure S12a of the supplementary document is as follows.

> *"The ratio of kinetic energy to internal energy is 0.68%, which is below the 5% threshold, confirming that the loading rate in the simulation is appropriately set and meets the requirements for quasi-static loading."*

Remark 1.5. In the results of Figure 3, nearly hybridization combinations lead to brittle hybrid designs out of combinations of brittle and ductile constituents. What is the mechanism driving the overall mechanical performance? While gains in the yield strength for certain designs are reported, the overall energy absorption appears to be negatively impacted. What could be potential mitigation strategies?

We thank the reviewer for this insightful comment. The predominantly brittle responses observed across several hybrid configurations arise from stiffness mismatches between the constituent architectures. In the studied cases, the strut-based lattices (*e.g.*, BCC and FCC) generally exhibit lower stiffness than the TPMS sheet structures. As a result, these strut-based components fail earlier under compressive loading, leading to brittle fracture and limiting the overall energy absorption of the hybrid system.

However, the experimental results for the G_S hybrid structure (Figure 3) demonstrate that this limitation can be mitigated by appropriately matching the stiffnesses of the constituent architectures. In this configuration, the TPMS component exhibits lower stiffness than the strut-based component, thereby delaying the premature failure of the low-energy-absorption phase and enabling the hybrid structure to maintain both high stiffness and enhanced energy absorption capacity.

These observations suggest a general design guideline for hybrid lattice systems. For

applications requiring improved energy absorption, the stiffness of the low-energy-absorption should be controlled to exceed that of the high-energy-absorption constituent, thereby delaying fracture initiation and promoting more distributed deformation. This mechanism allows the hybrid architecture to achieve a more favorable balance between stiffness and energy absorption.

To clarify this mechanism and highlight the corresponding design strategy, we have expanded the discussion (in red text) in the sixth paragraph of Section 2.2 of the revised manuscript as follows:

> *"Moreover, hybridization approaches often result in structural responses that are of a low-energy-absorption nature when combining inherently low-energy-absorption (i.e., early-failure) constituents with high-energy-absorption constituents. This occurs because low-energy-absorption lattice structures generally exhibit lower stiffness (e.g., BCC or FCC architectures typically possess lower elastic modulus than the TPMS structures with which they are combined) and therefore reach their failure threshold earlier, initiating early fracture. However, the experimental results for the G_S hybrid structure in Figure 3d provide evidence that both high energy absorption and high stiffness can be achieved simultaneously. In this configuration, the TPMS component exhibits lower stiffness than the strut-based component, suggesting a potential design guideline for applications requiring high energy absorption: the stiffness of the low-energy-absorption constituent should be controlled to exceed that of the high-energy-absorption constituent, thereby enabling synergistic enhancement of both stiffness and energy-absorbing capacity. It should be noted, however, that such a phenomenon occurs only when low-energy-absorption materials are used. In ductile materials, this issue does not arise."*

Remark 1.6. The development of biomimetic, lattice-based, tendon and ligament metamaterial designs has been explored in the literature. Relevant literature mechanical insights are however missing.

We thank the reviewer for this valuable suggestion. We agree that previous studies on biomimetic lattice-based designs for tendon-bone and ligament interfaces provide important mechanical insights that should be acknowledged. In response, we have expanded the discussion of the mechanical characteristics of the tendon-to-bone transition, particularly the stiffness gradient and anisotropic structural features that

govern load transfer in these biological interfaces.

Specifically, we have incorporated additional literature references and explanatory text discussing the biomechanical principles underlying tendon-bone attachment and their relevance to lattice-based implant design. These additions are included in the second paragraph of Section 2.6 ("Tendon-to-bone connection") of the revised manuscript and are highlighted in the revised text as follows:

> *"Furthermore, it has been demonstrated in [71] that elongated BCC lattice structures hold promise in meeting the anisotropic mechanical stiffness requirements of tendons, as their normal modulus significantly exceeds their shear modulus [72]."*

Remark 1.7. The same applies to the fatigue performance of TPMS-based architected cellular materials, where, inner topology-dependent fatigue mechanics have been extensively characterized. The study is missing a proper acknowledgement of existing contributions.

We thank the reviewer for this valuable comment. We agree that the fatigue performance of TPMS-based architected materials and the topology-dependent fatigue mechanisms reported in previous studies represent important contributions that should be acknowledged. In response, we have expanded the discussion of fatigue behavior in relation to the internal topology of lattice structures and incorporated additional references to relevant literature.

Specifically, we have added citations and explanatory text discussing topology-dependent fatigue mechanisms and their relevance to TPMS-based architectures in Section 2.4 (Fatigue behavior of hybrid lattices) and Section 4.4 of the revised manuscript (in red text). These additions help place the present results within the broader context of existing studies on fatigue performance in architected cellular materials. The corresponding revisions are as follows:

> *"While these findings share a similar trend with earlier studies [65, 66], differences exist due to variations in the materials used."*
>
> *"Numerous studies [29, 65, 80] have investigated the fatigue response of TPMS porous structures, including topologies such as G, D, and P, revealing distinct fatigue behaviors across different architectures. Based on the fatigue responses reported for these TPMS structures, we conclude that fatigue tests are typically conducted with failure criteria defined as 10^6 cycles, a specified level of stiffness*

loss, or structural fracture. The applied cyclic loads in these tests generally range from 20% to 80% of the yield strength, with a majority concentrated between 50% and 80%."

Remark 1.8. A description of the limitations of the current approach along with the need for further research perspectives is missing from the analysis.

We thank the reviewer for this comment. As suggested by the reviewer, we have expanded the Conclusion section to explicitly describe the current limitations of the proposed hybrid design strategy and to highlight potential directions for future work. In particular, we clarify that the present framework is primarily applicable to strut-based architectures with specific nodal characteristics that can be matched with TPMS skeletal representations. We also discuss future research opportunities to extend the method to more general lattice topologies by developing unit-cell-level sheet-topology blending functions, which could enable the design of customized transition structures for arbitrary nodal configurations. The revised text in Conclusions (last paragraph, in red text) is as follows:

"Although the strut-sheet hybrid design strategy proposed in this study is currently primarily applicable to strut-based structures with specific nodal characteristics, it already covers most common beam-based structural topologies. By further developing unit-cell-level sheet-topology blending function construction methods, it should be possible to generate customized transition structures tailored to arbitrary nodal configurations. Such developments will significantly expand the design space of porous materials, enabling next-generation structural materials to integrate multiple performance advantages, such as lightweight characteristics, high stiffness, and enhanced energy absorption, within a single architecture. Consequently, these advances could meet the increasing demand for advanced porous materials in complex future applications, particularly in aerospace and biomedical engineering."

Remark 1.9. The presentation of Figure 4 c-e needs to be enhanced. In its current state, the relevant analysis hardly interpretable.

We thank the reviewer for pointing out this issue. To improve the clarity and readability of Figures 4c-e, we revised the figure presentation by adopting a consistent color scheme to distinguish the structural categories and enlarging the graphical elements to

reduce visual complexity. In addition, we have added clearer legend descriptions and explanatory text in Section 2.3 of the manuscript to facilitate interpretation of the results. These revisions (in red text) in the updated manuscript are as follows:

"(shown in magenta and formed by combining the geometric patterns of the constituent topologies) exhibited mechanical properties that fall between those of their constituting substructures. The black blocks represent the strut structures, while the red wireframes denote the TPMS structure, as shown in Figures 4c-e."

"At equivalent relative densities, strut-based structures (blue blocks in Figure 4) generally exhibit lower specific surface areas than TPMS sheet architectures (red wireframes in Figure 4d). Hybrid structures (shown in purple in Figure 4d), however, demonstrated an intermediate specific surface area, effectively bridging gaps that would be challenging to address using a single structure type."

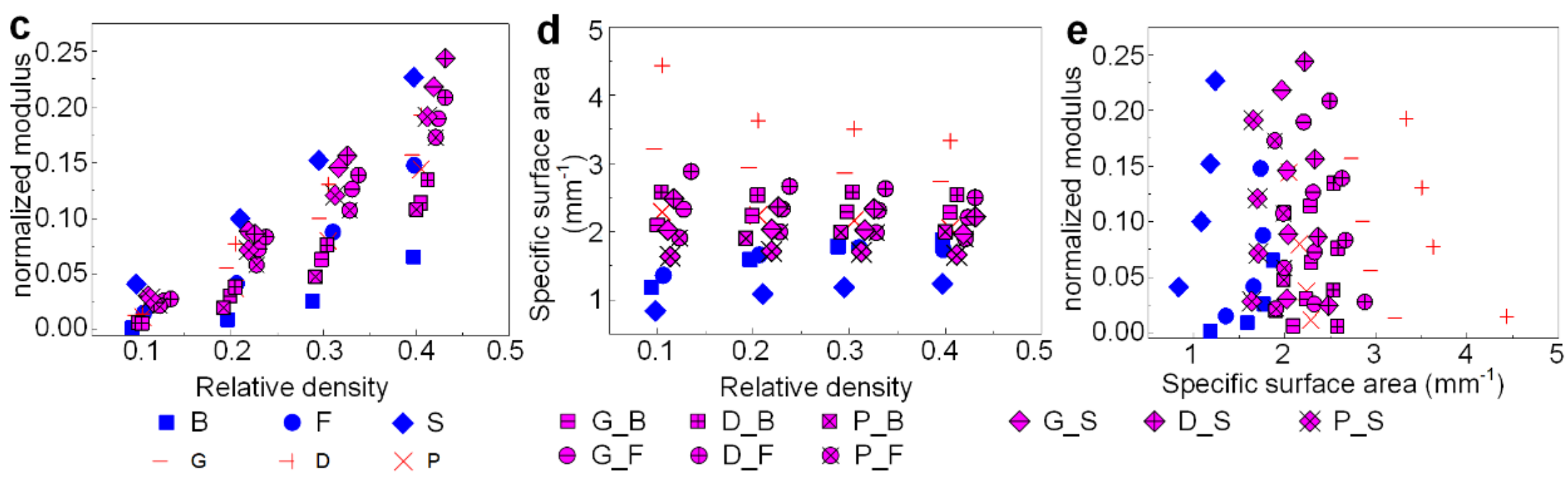

Remark 1.10. The language of the manuscript needs to be thoroughly proof-read and different typos and grammatical inconsistencies appearing at various positions have to be corrected.

We thank the reviewer for this helpful comment. The entire manuscript has now been carefully proofread to correct typographical errors, grammatical inconsistencies, and unclear expressions.

Reviewer #2

Remark 2.1. The manuscript presents a computational design workflow that combines closed-form triply periodic minimal surface (TPMS) sheet representations with strut-based lattices via a "twinned unit cell" transition strategy to realize hybrid, conformal load-bearing structures. While related graded and multi-architecture approaches exist, the claimed novelty is an automated, geometry-aware transition that preserves connectivity between sheet- and strut-based lattices and, together with voxel "corrosion/expansion," aims to enable precise stiffness modulation and performance

prediction. The 3D printing of high-performance, functionally distinct architected materials remains an important topic, and this manuscript demonstrates hybrid conformal structures and a biomimetic bone-to-tendon junction within a single continuous volume. The pipeline's compatibility with finite element modeling (FEM) and additive manufacturing, and the demonstration of programmable transitions across uncertain geometries, suggest potential applications in biomedical implants and structural engineering.

My main concern centers on novelty in light of prior strategies for graded lattices, multi-architecture blends (including sheet/TPMS blends), and topology-optimized interfaces, which already address many of the stated challenges (e.g., interfacial stress management, density control, and manufacturability). The present work appears to differ primarily in the specific "twinned unit cell" matching of nodal configurations and a voxel-based implementation, rather than introducing a fundamentally new interface paradigm. In addition, the scope appears quite narrow: the manuscript focuses on connecting two or more distinct lattice phases. For simple load cases, a single architecture with graded density or thickness often suffices; for complex, spatially varying loads, continuously changing architectures (within a single topology family or via smooth morphology transitions) may be more appropriate than discrete multi-phase hybrids. The manuscript does not clearly justify when and why discrete phase-to-phase connections are preferable, which limits perceived generality and raises questions about fit for a broad, general-interest journal.

Aside from concerns on novelty and scope, I offer the following major and minor critiques that would improve the quality of this manuscript. Note that some comments apply throughout and only call out a few example instances, with too many to cite them all.

We thank the reviewer for the careful reading of our manuscript and for providing this detailed summary and thoughtful critique. We appreciate the reviewer's recognition of the potential relevance of the proposed hybrid lattice design strategy and its possible applications in biomedical implants and structural engineering.

We acknowledge the reviewer's concerns regarding the novelty and generality of the proposed approach relative to existing strategies for graded lattices, multi-architecture blends, and topology-optimized interfaces. In response to these comments, we have revised the manuscript to more clearly articulate the conceptual contribution of the twinned unit-cell strategy and to clarify the scenarios in which discrete topology-to-

topology connections are advantageous over continuous gradient approaches.
In particular, we have expanded the discussion in the Introduction to better position our work relative to existing hybridization strategies and to explain the specific challenges associated with connecting fundamentally different lattice types (*e.g.*, TPMS sheet structures and strut-based lattices). We have also clarified the design motivations and application scenarios where discrete hybrid interfaces are beneficial, such as systems requiring abrupt mechanical or functional transitions within limited spatial domains.

Remark 2.2. The direct connection of strut-based and sheet (TPMS) lattices is a relatively niche use case with limited demonstrated usefulness and has been addressed previously. The manuscript asserts that this is an important scenario but does not substantiate why discrete phase-to-phase connections are needed. Overall, the contribution reads more like an engineering solution than a fundamental scientific advance.

We thank the reviewer for this constructive comment. We acknowledge the reviewer's concern regarding the novelty and generality of the proposed approach and have revised the manuscript to clarify both the conceptual contribution and the application scenarios where discrete topology-to-topology connections are advantageous.

Existing hybrid lattice design strategies typically rely on geometric interpolation, density grading, or smooth morphological transitions within a single topology family. While these approaches are effective for gradual property variation, they can encounter limitations when connecting fundamentally different lattice classes, such as strut-based and TPMS sheet architectures. In such cases, interpolation-based transitions may produce poorly controlled intermediate geometries, unpredictable mechanical behavior, or extreme geometric features that compromise manufacturability.

The key contribution of this work is the introduction of a twinned-unit-cell transition strategy that establishes geometric compatibility between different topology families by exploiting the nodal correspondence between strut lattices and TPMS skeletal representations. By selecting TPMS skeletal configurations with matching nodal connectivity, the transition region can be constructed using structures that retain explicit mathematical representations. This enables controllable and mechanically robust interfaces between otherwise incompatible lattice architectures.

Regarding the reviewer's question of when discrete phase-to-phase connections are preferable, such transitions become important when abrupt changes in mechanical or

functional properties are required within a limited spatial domain. Examples include biological attachment interfaces, such as the tendon-to-bone junction, and engineered structures that require integrating load-bearing cores with compliant boundary regions. In these cases, continuous gradients within a single topology may require long transition lengths or fail to provide the required combination of structural functions.

We agree that this motivation was not sufficiently articulated in the original manuscript. Therefore, we have revised and expanded the Introduction to clarify the limitations of existing approaches and to better justify the use of discrete topology connections in the targeted application scenarios.

Finally, we respectfully emphasize that the contribution extends beyond a purely engineering implementation. The study introduces a generalizable strategy for connecting heterogeneous lattice topologies *via* controllable transition architectures and demonstrates that pixel-level operations can systematically modulate the mechanical performance of transition regions. We have revised both the Introduction and Conclusion sections (in red/blue text) to more clearly highlight these conceptual contributions as follows:

> *"To tailor the mechanical behavior of architected materials, researchers have widely explored functional gradient strategies, in which structural parameters vary gradually throughout the architecture. These gradients can be implemented through changes in strut diameter or surface thickness [20, 21], scaling of unit cells [22], or controlled deformation of unit cells through compression or elongation [23-25]. Such approaches typically generate transitions between identical or closely related topologies [26, 27]. Gradient strategies employing linear [28, 29], step-wise [30, 31], or exponential [32, 33] transitions have been shown to enhance the mechanical performance of architected structures in diverse applications [34], including improved energy absorption in crash-protection systems [31, 35] and the modulation of mechanical cues in bone tissue scaffolds [36, 37] to mitigate stress shielding and promote bone regeneration [38]. TPMS architectures are particularly well suited to such gradient designs because they are defined by continuous mathematical surfaces [39-43]. In contrast, strut-based lattices generally lack a continuous analytical description, which makes the realization of smooth geometric transitions considerably more challenging."* (Introduction)

> *"However, many natural and engineered systems require abrupt, dramatic*

transitions in structural and mechanical properties, rather than gradual gradients. A prominent example is the bone-tendon interface, where a stiff, porous bone structure connects to a highly compliant fibrous tendon within a relatively short transition zone [44]. Similarly, engineered porous components that must simultaneously provide high load-bearing capacity and conformal compliance may require integrating stiff core architectures with highly deformable boundary regions [17]. In such cases, the optimal structural solution may involve combining fundamentally different lattice topologies, for example, a high-stiffness TPMS sheet architecture with a compliant strut-based lattice. Conventional gradient strategies are poorly suited to such problems." (Introduction)

"By further developing unit cell-level sheet topology blending function construction methods, it should become possible to generate customized transition structures tailored to arbitrary nodal configurations. Such developments will significantly expand the design space of porous materials, enabling next-generation structural materials to integrate multiple performance advantages, such as lightweight characteristics, high stiffness, and enhanced energy absorption, within a single architecture. Consequently, these advances could meet the increasing demand for advanced porous materials in complex future applications, particularly in aerospace and biomedical engineering." (Conclusion)

Remark 2.3. Many established approaches already address this problem, including those the authors cite (e.g., graded lattices and topology-optimized interfaces). The authors argue that existing solutions concentrate stresses in the boundary layer, yet the proposed method similarly focuses interface stresses within a single-cell-thick layer. This may be acceptable at structural scales, but for metamaterials—where the unit cell is small relative to the overall dimensions—such sharp transitions can be problematic and are often better handled via continuous gradients. As a result, the claim of a substantial gap in the literature is not convincing.

We thank the reviewer for this thoughtful and technically important comment. We agree that extensive research has been conducted on lattice connections through approaches such as graded architectures and topology-optimized interfaces, and we do not intend to suggest that this broader problem has not been previously studied.

Our motivation instead focuses on the specific challenge of connecting fundamentally different topology classes, particularly strut-based and TPMS sheet architectures. In

many previous approaches addressing such heterogeneous connections, geometric interpolation or distance-field blending is employed to generate intermediate geometries. However, when applied to highly dissimilar topologies, these interpolation-based methods can produce poorly controlled intermediate structures, including irregular geometric features and localized stress concentrations.

We acknowledge that the earlier manuscript description may have been overly general in its treatment of stress concentrations in transitional structures. Our intention was specifically to highlight the stress concentrations that may arise from geometric interpolation between highly dissimilar topologies, rather than implying that all graded or topology-optimized transitions inherently exhibit this issue. The manuscript has been revised to clarify this point in the revised Introduction, as follows:

> *"Achieving large property contrasts within a single topology typically requires drastic geometric modifications that produce extreme feature sizes [45], introduce manufacturing constraints [46], or generate severe stress concentrations [47]."*

Regarding the reviewer's concern about the single-unit-cell transition region, this design choice was intentional. The objective of the proposed framework is to maintain geometric continuity while minimizing the spatial extent of the transition region, thereby preserving the functional characteristics of the constituent lattice architectures. This is particularly relevant in applications where abrupt property changes are required within limited spatial domains, such as biological attachment interfaces (*e.g.*, tendon-bone connections) or engineered structures integrating load-bearing and compliant regions.

Importantly, the proposed method is not restricted to single-cell transition intervals. As shown in Figures S10b-e of the supplementary document, the transition interval can be adjusted by modifying the parameter k, allowing the framework to accommodate both short and extended transition regions. Additional clarification regarding this flexibility has been added following Equation 6 in Section 4.1 of the revised manuscript:

> *"It should be noted that at the macroscopic scale, an excessively large transition region is undesirable, as it would reduce the proportion of each constituent architecture and diminish the value of the hybrid design, particularly given the fact the available number of transitory unit cells is often limited by the overall size of the construct and the resolution of the 3D printing technique. This consideration is particularly relevant in applications such as bone-to-tendon interfaces and*

integrated load-bearing-conformal systems. Therefore, in this study, we constrain the transition length to one unit cell size to create twinned unit cells (Figure S10b-e of the supplementary document). For applications requiring extended transition zones, the analytical framework provided (Figure S10 of the supplementary document) can be referenced for tailored adjustment."

Remark 2.4. The manuscript acknowledges that gradient-based designs are common and useful but states that "their application within single-architecture structures often results in porosity variations." Please clarify: Why is this a problem? What are the specific exceptions to this claim ("often"), and what shortcomings do those exceptions have, if any?

We thank the reviewer for this important clarification request. The concern regarding porosity variation arises when large differences in mechanical performance, such as stiffness or load-bearing capacity, are required between adjacent regions of a structure. Within a single-topology gradient design, achieving such large property differences typically requires substantial geometric changes (*e.g.*, large variations in strut diameter or wall thickness), which can lead to significant differences in porosity between neighboring regions. These extreme geometric variations may introduce manufacturing challenges, structural irregularities, or localized stress concentrations.

We agree that the wording in the original manuscript may have been overly general. Gradient-based approaches are highly effective in many cases where moderate and continuous property variations are sufficient. However, difficulties arise in scenarios that require abrupt functional transitions within limited spatial regions, such as biological attachment interfaces (*e.g.*, tendon-bone connections) or structures that combine stiff load-bearing regions with highly compliant sections.

To address the reviewer's concern, we have revised the wording in the Introduction to clarify that such limitations arise primarily when large property contrasts must be achieved within the same topology. In these cases, drastic geometric modifications may be required, potentially leading to extreme feature sizes, manufacturability challenges, or stress concentrations. The revised text has been highlighted in the manuscript (fourth paragraph of the Introduction, marked in blue).

Remark 2.5. The statement that "excessive porosity adversely affects manufacturability" is not substantiated. If a process can fabricate both strut-based and

sheet lattices, it should plausibly be able to fabricate interfaces with modestly different porosity distributions. Please provide evidence that directly tie porosity levels at the interface to manufacturability limits.

We thank the reviewer for raising this point. We agree that additive manufacturing processes capable of fabricating both strut-based and sheet-based lattices can generally accommodate moderate variations in porosity. Our intention was not to suggest that porosity variation alone inherently prevents manufacturability.

Rather, the concern arises when large mechanical-property differences must be achieved within a single topology using gradient-based approaches. In such cases, substantial geometric modifications, such as large variations in strut diameter or sheet thickness, may be required. These modifications can lead to extremely thin structural features, which may approach or fall below the minimum feature size supported by a given manufacturing process, thereby reducing fabrication reliability.

In contrast, the hybridization strategy proposed in this work enables significantly different mechanical properties to be achieved without requiring extreme geometric scaling, as distinct lattice topologies can exhibit distinct stiffness and deformation behavior at comparable relative densities. As a result, the interface can maintain structural dimensions within a more manufacturable range.

To clarify this point, we have revised the Introduction to better distinguish between porosity variation and geometric-feature-size limitations that may arise in single-topology gradient designs. The revised text has been highlighted in the manuscript (fourth paragraph of the Introduction, marked in blue).

Remark 2.6. The introduction states that a flaw of existing solutions is that the local relative density of the transition cell deviates from that of surrounding cells. However, this also appears to occur in your approach (e.g., Fig. 2c). Please reconcile this inconsistency.

We thank the reviewer for raising this important point. We agree that deviations in local relative density can also occur in our approach, as shown in Figure 2c, and we appreciate the opportunity to clarify this distinction.

In the original manuscript, the concern about abrupt relative density changes specifically referred to situations in which large property contrasts must be achieved within a single topology using gradient-based designs. In such cases, achieving substantial stiffness differences often requires extreme geometric scaling (*e.g.*, large

variations in strut diameter or wall thickness), which can lead to irregular relative-density distributions and potentially impractical structural features.

In our approach, the transition region is constructed by matching the wall thickness or strut diameter of the adjacent structures to ensure geometric continuity at the interface. Consequently, some deviation in relative density may occur within the transition cells when connecting structures with different geometrical parameters. However, unlike uncontrolled deviations arising from geometric interpolation, the relative density in our transition region can be systematically adjusted using the proposed voxel erosion/dilation operations.

To clarify this point and demonstrate controllability, we have added additional discussion and data in the last two paragraphs of Section 2.3 (in blue text). In addition, Figure S7 of the supplementary document has been included to illustrate how the relative density distribution in the transition region can be actively tuned. We have also described the relative density compensation procedure used to correct modeling deviations in Section 4.1 (in blue text) of the revised manuscript. All these changes are presented below.

"By adjusting the remaining wall thickness, the relative density of the transition cell can be modulated, thereby influencing its stiffness. Computational results indicated that reducing wall thickness (with relative density decreasing from 52.32% to 35.49%) leads to a significant decline in the normalized modulus in the x_3 direction, from an initial 0.3363 (uncorroded state) to 0.1372 (i.e., approximately 40% of its original value) (Figure 4f). This approach allows the transition cell's mechanical properties to be tuned to align with those of adjacent structures.

Conversely, in cases where the transition cell was less stiff than its constituent structures, we proposed "voxel dilation", a technique that increases wall thickness to enhance mechanical properties (Figure 4b). The transparent region in Figure 4b represents the dilated wall. Similar to voxel erosion, material thickening was restricted along the x_2 boundary planes to maintain connectivity. Following dilation, the compressive modulus of the transition cell increased proportionally with the wall thickness. For instance, adding 6 voxels (increasing relative density from 27.10% to 57.34%) resulted in a 5-fold increase in the normalized modulus in the x_3 direction (Figure 4g)."

"

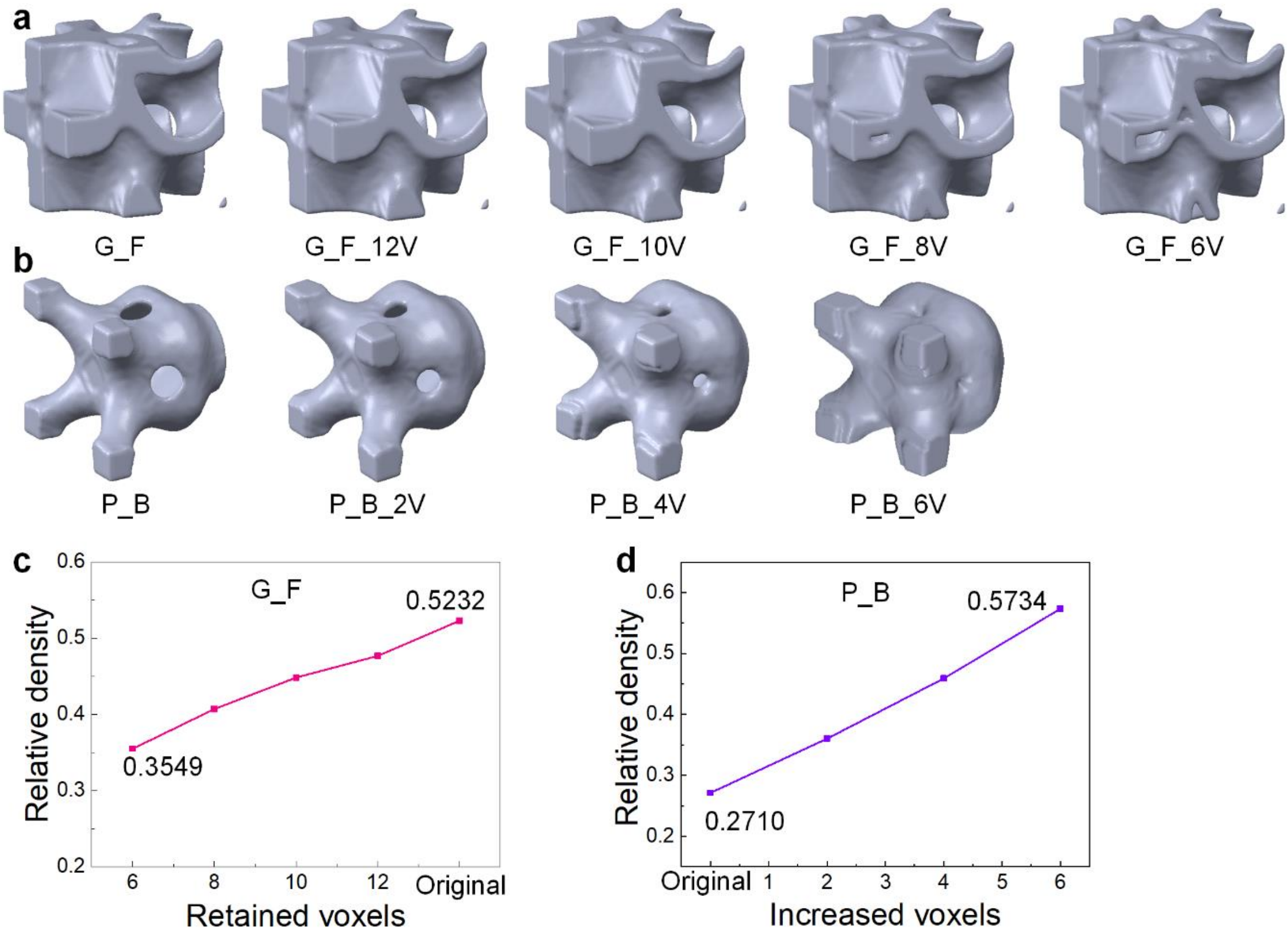

Figure S7. *Relative density regulation of transitional unit cells. (a) G_F transitional cell, (b) P_B transitional cell, (c) Relationship between the relative density of the G_F transitional cell and the adjusted voxel size, (d) Relationship between the relative density of the P_B transitional cell and the adjusted voxel size.*

"Figure S7 demonstrates the regulation of relative density in transitional unit cells. When reducing relative density, the erosion method is employed, as illustrated in (a) for the G_F transitional cell. Here, the notation "XV" (where X is a numerical value) indicates erosion to a wall thickness of X voxels. Conversely, when increasing relative density, the dilation method is used, as shown in (b) for the P_B transitional cell, where "XV" signifies dilation by X voxels in wall thickness. All operations are performed while preserving the connectivity of the boundary interfaces. Figures (c) and (d) present the relative density curves obtained under different wall-thickness adjustments, demonstrating effective and precise control over the relative density."

"The relative density control and compensation mechanism operates as follows. First, relative density is defined only for the constituent structures. Once the

relative density of each constituent structure is specified, transition cells are automatically generated based on neighboring structures to ensure smooth connectivity. This process uses the target relative density of each constituent unit to determine the corresponding wall thickness (for TPMS structures) or strut diameter (for strut-based structures). These derived dimensions are then used as the target wall thickness or strut diameter for the transition cells. As a result, deviations in the relative density of transition cells may occur (e.g., Figure 2c). However, the trend of variation remains close to the maximum values in neighboring regions and can be adjusted using the proposed voxel-erosion method. Compensation was implemented in two steps. First, systematic errors inherent to the MATLAB algorithm used to generate printable files were corrected by analyzing topology-specific deviations. For example, if a target relative density of 0.30 produced an actual density of 0.29, a compensation factor Pcom = 0.30 / 0.29 was applied, and the corrected target value became 0.30 ×Pcom (see Figure S10f of the supplementary document for details). For the G, D, P, B, S, and F topologies fabricated in this study, the compensation factors were 0.923, 0.860, 0.917, 0.950, 0.967, and 0.940, respectively.

Second, a methodological compensation step was implemented based on the analysis presented in Figure 4f, which relates the number of eroded or dilated voxels in transition unit cells to the relative density of the interval. Figure S7 of the supplementary document illustrates the adjusted relative density distribution across the transition cells. In practice, the target relative density can be approached iteratively via controlled voxel erosion or dilation. According to the analysis shown in Figure S10f of the supplementary document, the residual relative density error after the first compensation step is already within an acceptable range. Therefore, when strict accuracy in the relative density of transition unit cells is not required, the second compensation step may be omitted."

Remark 2.7. Computational analyses are not typically used to validate an approach, as stated at the end of the introduction. Please clarify and/or revise the methodology to follow standard practice: for example, use experiments to validate the modeling framework (including the transition strategy), and then use simulations to explore the parameter space within validated bounds.

We thank the reviewer for this suggestion. We agree that computational analyses should

not be described as validating the approach. Our intended workflow follows the reviewer's suggested standard practice.

Specifically, we first used experiments to validate the modeling framework and the proposed transition strategy by fabricating representative hybrid structures and evaluating their mechanical and fatigue behavior. The simulation framework was then calibrated and verified against these experimental results. After this validation step, the computational model was used to systematically explore the broader design parameter space, including variations in relative density and structural configurations.

To avoid confusion and better reflect this workflow, we have revised the penultimate paragraph (in blue text) of the Introduction to clarify that experiments are used to validate the modeling framework, while simulations are employed to analyze the parameter space within experimentally validated bounds.

> *"We validate the proposed framework through a combination of experiments and numerical simulations. Mechanical testing was used to evaluate how different connection strategies influence stiffness, failure mechanisms, and fatigue behavior of the hybrid lattices, while numerical simulations were employed to explore the broader parameter space within experimentally validated limits. Finally, we demonstrated the potential of the approach through several application-driven examples, including the design of a biomimetic tendon-to-bone interface (Figure 1b)."*

Remark 1.8. The load cases explored are too narrow. Hybrid lattices will experience a variety of loading modes and orientations. At a minimum, include tests and simulations in tension, bending, and torsion, and probe multiple load orientations relative to the interface. This broader evidence base is also necessary to justify the choice of two discrete architecture types versus a continuously varying architecture tailored to local stress states.

We thank the reviewer for this suggestion. We agree that hybrid lattice structures may experience multiple loading modes and orientations in practical applications, and that evaluating their response under diverse loading conditions is important.

Following the reviewer's recommendation, we have expanded the experimental investigation to include tension, bending, torsion, and combined loading scenarios (*i.e.*, torsion-compression and torsion-tension). These additional experiments were conducted to further evaluate the robustness and performance of the proposed hybrid

connection strategy under different mechanical conditions.

The corresponding results have been incorporated into Section 2.2 of the revised manuscript, and the associated experimental procedures have been described in Section 4.4. The new results are presented in Figure 3d, e, and Figure S5 of the supplementary document, and supplementary videos S5-S7.

These results demonstrate that the hybrid structures maintain stable structural behavior across different loading modes and exhibit a balanced mechanical response that integrates the advantages of the constituent architectures, such as achieving a favorable combination of load-bearing stiffness and fracture strain.

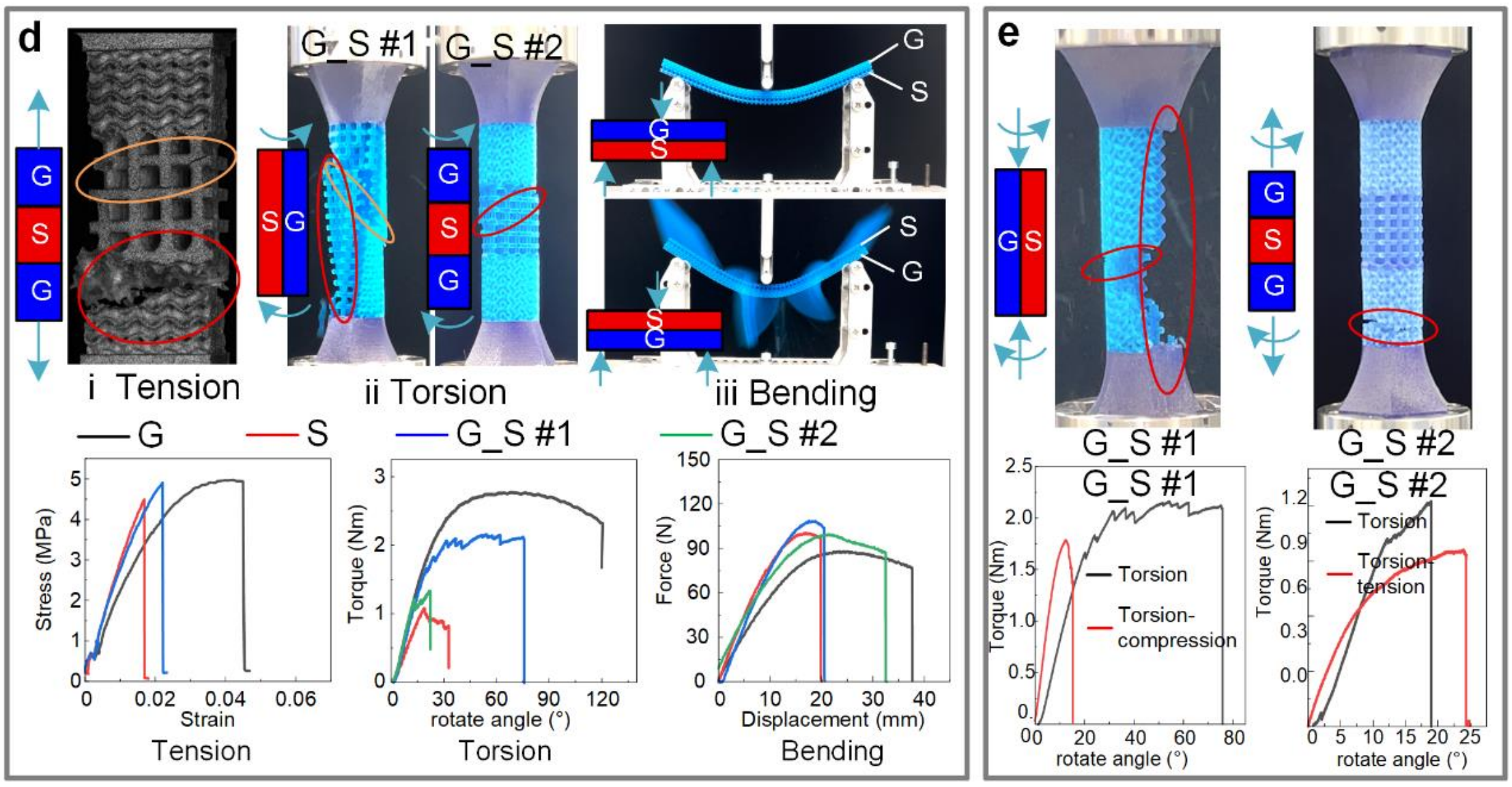

Figure 3. *A comparative study of experimental tests. The failure snapshots of the G_S hybrid structure under (d) tensile, torsional, bending, and (e) biaxial loading, as well as the corresponding stress/force/torque versus strain/angle/displacement curves.*

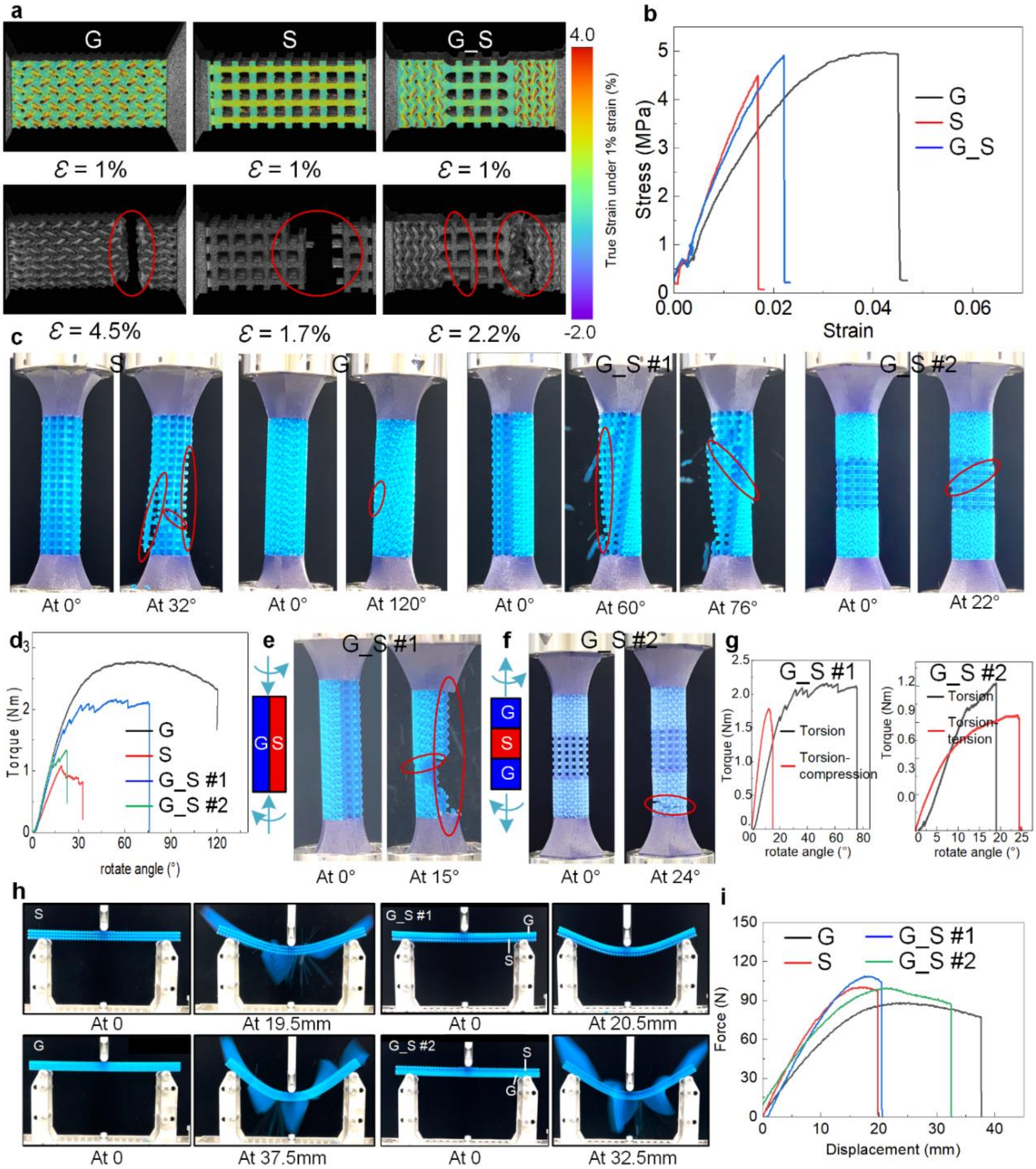

Figure S5. *Deformation response snapshots of the G, S, and their constituent hybrid structures under various loading conditions: (a) tensile condition, (c) torsional condition, (e) biaxial tension–torsion condition, and (f) biaxial compression–torsion condition. Their corresponding torque/stress/force vs. angle/strain/displacement curves: (b) tensile condition, (d) torsional condition, (g) torsional condition and biaxial conditions, and (i) bending condition.*

"To further evaluate the robustness of the proposed design under different loading conditions, additional mechanical tests were performed under tension, torsion, bending, and combined loading (Figures 3d and 3e, and Figure S5 in the supplementary document, and supplementary movies S5-S7). In all cases, failure consistently occurred within the constituent substructures rather than at the

connecting interfaces, indicating that the transitional structures effectively resist deformation-induced failure (Figure S5a, c, e, and f).

For example, under tensile loading, the G-S hybrid structure exhibited complete fracture in the G substructure region (red circle in Fig. 3d i) and visible cracks in the S substructure region (orange circle in Fig. 3d i), while no significant damage was observed in the transition region. When subjected to torsion, the S structure was prone to bending shear coupling failure, with stress concentrating at the nodes, leading to brittle fracture. In contrast, the curved connectivity of the G structure enabled uniform stress distribution, achieving large deformation and energy absorption through cell wall bending and buckling (Figure S5a). In the hybrid G-S structure, the transition region alleviated stress concentration by dispersing localized stress into adjacent cells, resulting in substantial fracture first occurring within the S substructure region (red circle in Fig. 3d ii). Following this fracture, the transition region delayed crack initiation and a further increase in the torsion angle was required to induce cracks in the G substructure region, ultimately leading to complete fracture (orange circle in Fig. 3d ii). Compared to the series hybrid configuration (G-S#2), this compromise effect was more pronounced in the parallel hybrid configuration (G-S#1).

Under bending loading, although both single and hybrid structures exhibited sudden fracture (making it difficult to capture the fracture moment in videos; the images shown are screenshots with motion blur before and after fracture, Figure S5f), it was evident that the hybrid structure with the G component placed near the indenter (G-S#1) exhibited a bending deflection closer to that of the S structure, whereas the hybrid structure with the opposite arrangement (G-S#2) showed a bending deflection more similar to that of the G structure. This demonstrates the significance of hybridizing G and S structures, as their different configurations yield varying levels of bending resistance. In other words, by strategically arranging G and S structures, it is possible to program structural performance to meet engineering requirements.

Furthermore, the deformation curves further reveal that the G structure exhibits greater fracture strain or displacement under tensile and bending loads, demonstrating superior fracture toughness, whereas the S structure possesses a higher elastic modulus, indicating a greater load-bearing capacity (Fig. 3d). By hybridizing these two structures through the proposed method, a balanced

mechanical response is achieved, integrating the advantages of the constituent substructures to obtain moderate fracture toughness and load-bearing capacity. This approach overcomes the inherent trade-off between stiffness and energy absorption that is commonly encountered in traditional lattice designs. Under torsional loading, because the S structure is not well-suited to resisting torsion, its presence in the parallel hybrid configuration (G-S#1) accounts for half of the structure, significantly reducing the torsional resistance of the G structure. In the series hybrid configuration (G-S#2), failure is governed by the weakest part, where the failure of the S structure directly leads to structural disconnection. Although neither the series nor the parallel hybrid structure surpasses the performance limit of the G structure, they still exhibit a compromise in performance, characteristic of hybrid configurations.

Under biaxial loading conditions, it is observed that, in the parallel configuration (G_S #1) under compressive-torsional loading, failure predominantly occurs in the S-shaped region. All S-shaped structures undergo instantaneous fracture at the failure strain, with damage rapidly propagating into the transitional structure and the G-shaped region. In contrast, for the series configuration (G_S #2) under tensile-torsional loading, the fracture morphology resembles that observed under pure tensile loading, with failure mainly localizing in the G-shaped region rather than exhibiting the shear-dominated failure characteristics of the S-shaped region seen under pure torsion. These findings indicate that under biaxial loading, tension and compression dominate structural failure, and failure behavior is governed by the load-bearing capacity of the constituent structures. The transitional structure does not fail first and effectively serves as a functional connector. Further statistical analysis of the torque – rotation angle curves reveals that under compressive-torsional loading, the G_S #1 configuration can sustain higher torque at smaller rotation angles (i.e., higher torsional stiffness), requiring greater resistance to failure. However, due to the dominance of compressive loading, complete failure occurs at an earlier stage."

Remark 2.9. To support the claim that the proposed method is significantly better than alternatives, provide direct, quantitative benchmarking against representative gradient-based and topology-optimized interface strategies (e.g., comparing stiffness, strength, fatigue resistance, interfacial stress distribution, and manufacturability metrics at matched relative density).

We thank the reviewer for this important suggestion. We agree that benchmarking against existing hybrid or graded design strategies is valuable for evaluating the proposed approach's performance.

However, performing a direct comparison with gradient-based or topology-optimized interface strategies is challenging in this case because the primary focus of the present work is the connection between fundamentally different lattice classes, namely strut-based and sheet (TPMS) architectures. To the best of our knowledge, existing gradient-based or topology-optimization approaches have rarely been applied specifically to hybrid interfaces between these two topology families, making a strictly equivalent comparison difficult.

To nevertheless provide a meaningful performance evaluation, we have introduced a comparative benchmarking analysis across several representative hybridization strategies, including strut-strut, TPMS-TPMS, and multiphase interpenetrating architectures. To facilitate a consistent comparison, we introduced a normalized performance ratio metric, defined as the ratio between the mechanical performance of the hybrid structure and the average performance of its constituent single architectures at the same relative density.

Using this metric, values greater than 1 indicate synergistic enhancement, whereas values below 1 indicate that hybridization does not improve the combined performance. The evaluated properties include load-bearing stiffness, strength, fracture strain, and energy absorption capacity, which are typically difficult to optimize simultaneously in conventional lattice structures.

The results of this benchmarking analysis have been added to Figure 4j and discussed in the newly introduced Section 2.5 (Performance comparison). The comparison shows that the hybrid structures generated using the proposed strategy achieve more balanced and synergistic improvements across multiple mechanical properties, whereas other hybrid strategies typically improve one property at the expense of others.

We hope this additional analysis provides a clearer quantitative context for evaluating the advantages of the proposed method. All the added contents are as follows:

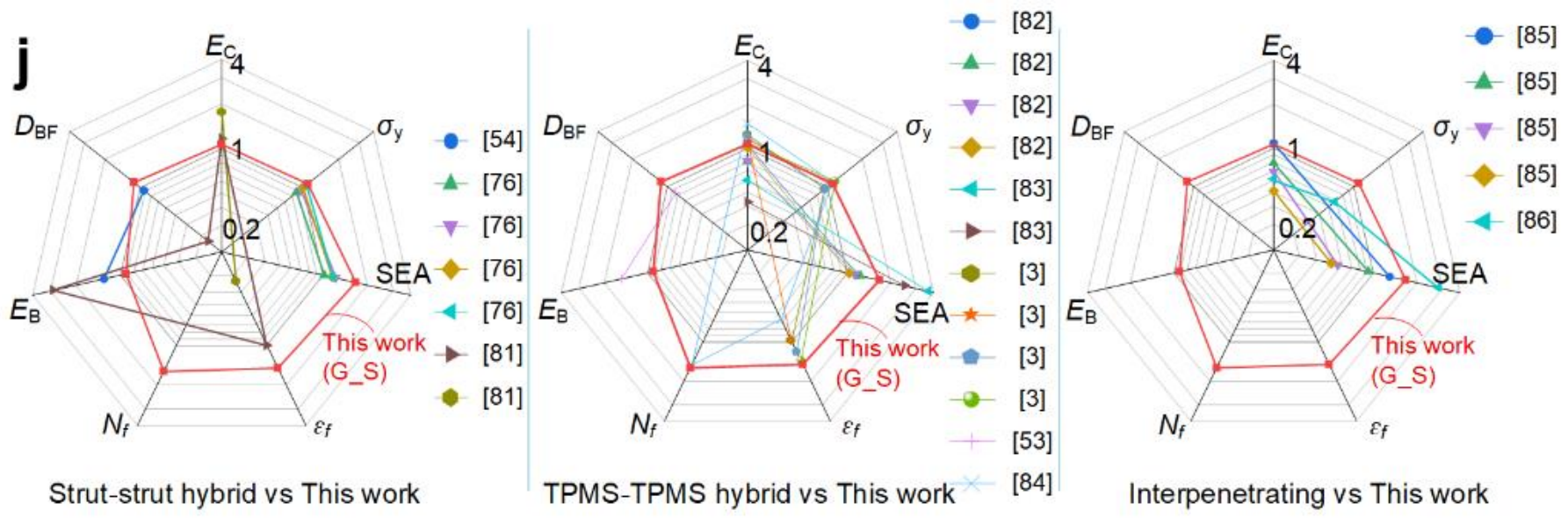

"As shown in Figure 4j, the performance ratio, defined as the performance value of the hybrid structure divided by the average performance of its corresponding constituent single structures, indicates that existing strut-strut hybrid structures generally fail to achieve a strongly synergistic enhancement in both load-bearing capacity and damage resistance (or energy absorption). For these structures, the connecting lines representing different performance ratios all intersect the average reference line (i.e., black dashed line). This indicates that although improvements can be achieved in one performance metric, they are typically accompanied by trade-offs that limit other properties.

TPMS-TPMS hybrid structures exhibit two distinct behaviors. One group further amplifies the aforementioned imbalance. For example, the hybrid structures represented by the golden circular and triangular symbols achieve high energy absorption but relatively low compressive stiffness (Figure 4j). The second group (cyan curve) exhibits a more balanced mechanical response, although the improvement in some properties remains modest (Figure 4j).

In contrast, the compressive performance ratios of the strut-TPMS hybrid structures generated using the proposed method (red curve) are consistently greater than 1, indicating that the hybrid structures outperform their constituent architectures on average (Figure 4j). This demonstrates that the proposed hybridization strategy enables a more effective balance between multiple mechanical properties, resulting in simultaneous improvements in structural stiffness, strength, fracture strain, and energy absorption capacity.

Although the flexural stiffness is slightly below the average reference value, the specimens used in the bending experiments were unoptimized structures representing only a simple combination of the constituent architectures (Figure S5h). Further improvements in overall mechanical performance are expected through optimization of the spatial distribution of the different substructures."

Remark 2.10. The methods lack sufficient detail for generalization and reproducibility. Important steps appear to rely on manual "compensatory adjustments," and code/parameters are not provided. Please specify the full pipeline, inputs, and decision rules, and share code or pseudocode to ensure reproducibility.

We thank the reviewer for this important comment regarding reproducibility. We agree that a clear description of the full computational pipeline, including key parameters and decision rules, is necessary to ensure that the proposed method can be reproduced and generalized.

In response, we have expanded the Methods section to provide a more detailed description of the workflow, including the design-domain definition, voxel-based discretization procedure, transition-cell identification, and the relative density compensation strategy. In particular, we have clarified the origin of the relative density deviations and the corresponding compensation procedure, which were previously only briefly mentioned in the supplementary document.

Specifically, the third and fourth-to-last paragraphs of the revised Section 4.1 have been expanded to describe the compensation methodology and the parameter values used in this study. In addition, the compensation factors applied to different lattice topologies are now explicitly reported.

To further improve reproducibility, we have also provided the MATLAB script used to generate the hybrid structures as Supplementary Code S8, along with instructions for its use. These additions clarify the full pipeline, required inputs, and implementation details necessary to reproduce the proposed design strategy.

Remark 2.11. Methodological rigor needs strengthening (in addition to the validation concern above). Please ensure clear problem statements, controlled comparisons, and complete reporting of assumptions, boundary conditions, and uncertainty.

We thank the reviewer for this important comment regarding methodological rigor. In response, we have revised and expanded the Methods section to improve the clarity of the problem formulation, the definition of modeling assumptions, and the reporting of boundary conditions and computational parameters.

Specifically, the methodological description has been restructured and consolidated in Section 4.1, now titled "Hybrid Structure Design and Generation Methodology." Sections 4.1 and 4.2 have been merged to provide a clearer, more coherent description of the computational workflow. Within this section, we have explicitly clarified the

problem setup, algorithmic inputs, controlled variables, and decision rules used in the hybrid structure generation process.

In addition, we have expanded the descriptions of the relative density control mechanism, compensation procedure, and modeling assumptions, and clarified the boundary conditions and parameters used in the finite element simulations. These revisions provide a more transparent description of the computational framework and ensure that the modeling approach and comparisons presented in the manuscript are clearly defined and reproducible.

> *"Integrating TPMS sheet-based (Figure 6a) and strut-based lattices presents a significant challenge due to their inherently different structural configurations. Achieving a continuous, smooth, and mechanically robust transition between these architectures is crucial to prevent geometric discontinuities (Figure 6b, first row). To address this, we developed a systematic connection strategy, which consisted of the following steps: i) twinned unit cell approach, in which we identified skeletal structures with nodal configurations resembling the target strut-based lattices as twin unit cells (Figure 6b, second row). ii) transition structure formation, in which we used a closed-loop mathematical expression to define both the TPMS sheet structures and their corresponding TPMS skeletal structures (Figure 6c) and generated the transition structure by weight value introduced in the two TPMS structures' mathematical function. iii) automated generation of the final hybrid structure, in which we retained the transition structure and replaced the skeletal structures with the target strut-based structure (Figure 6b, third row) in non-transitional areas …."*

Remark 2.12. A core contribution appears to be the automation of the transition structures. The algorithmic details should be described in the main text, not only stated as "automated." Please include the matching criteria, search/optimization strategy (if any), voxel operations (corrosion/expansion), parameter ranges, and computational cost. Also provide the limits of the generalizability.

We thank the reviewer for this comment regarding the transparency and reproducibility of the automated transition-structure generation algorithm. We agree that the algorithmic details should be described clearly in the manuscript rather than only referring to the process as "automated."

In response, we have revised and expanded the Methods section to provide a more

complete description of the computational workflow. Specifically, the previous Sections 4.1 and 4.2 have been merged into a new Section 4.1, titled "Hybrid Structure Design and Generation Methodology," to present the full pipeline in a clearer and more structured manner.

Within this section, we have now explicitly described:

- the matching criteria used to identify compatible transition nodes between the strut-based and TPMS skeletal representations,
- the voxel-based operations used to construct and regulate the transition structures (including voxel erosion and dilation),
- the algorithmic inputs and parameter ranges used in the structure-generation process,
- the computational cost associated with generating the hybrid structures used in this study,
- and the limitations and generalizability of the proposed approach.

To further ensure reproducibility, the complete MATLAB implementation used to generate the hybrid structures is provided in Supplementary Code S8, along with a read-me file describing the required inputs and usage instructions. Additional schematic details of the algorithm are also provided in Figure S9 of the supplementary document.

> *"To address this, we introduce a design-domain description approach that enables the automated generation of hybrid structures. A MATLAB script (version 2020a) was developed for this purpose, and the full code is provided as Supplementary Code S8. Detailed instructions for using the code are included in the accompanying README file. As illustrated in Figure 7, the workflow automatically generates voxel matrices representing the hybrid structures, which can be converted into ".INP" [77] and ".STL" [78] files. This framework enables the efficient fabrication of hybrid structures while ensuring compatibility with both 3D printing technologies and finite element method (FEM) simulations.*
>
> *The primary algorithmic inputs and controlled variables are as follows. 1) Design domain: The spatial regions corresponding to each substructure are defined using STL files (see Figure S9 of the supplementary materials for details). 2) Key parameters: The user specifies the number of design domains (nd, representing the number of substructures to be combined), the number of voxels per unit-cell edge (nv, defining the resolution), the voxel size (Lv, the voxel edge length determined by the unit cell size and nv), and the file number nin (corresponding to*

an STL-format file that defines the structural boundary, i.e., the design domain). These STL files must follow a prescribed naming convention and be stored in the program's working directory. After the number of design domains is entered, a second input window appears with corresponding fields for each substructure. In this window, the user specifies the structure name (Name), the relative density (RDi), and the structure type (Type: sheet or strut). The parameter nv is an integer, typically chosen as a multiple of 10. Larger values provide higher geometric resolution but increase computational cost (see Figure S10a in the supplementary document for a sensitivity analysis). The unit cell edge length is defined by the product nv × Lv. Relative density is assigned independently for each constituent structure, requiring nd separate relative density values.

The design-domain geometries (in .STL format) are discretized into voxels. Each voxel is then evaluated according to structural inclusion criteria. Voxels satisfying these criteria, based on the TPMS implicit function and nodal information describing strut structures, were retained and represented as a logical matrix. This matrix was subsequently used to construct the final printable output model in .STL format. A schematic overview of the entire process is shown in Figure S11 of the supplementary document. For structures intended for finite element simulation, the logical matrices were converted into .INP files, with mesh information formatted according to previously reported methods [77].

The relative density control and compensation mechanism operates as follows. First, relative density is defined only for the constituent structures. Once the relative density of each constituent structure is specified, transition cells are automatically generated based on neighboring structures to ensure smooth connectivity. This process uses the target relative density of each constituent unit to determine the corresponding wall thickness (for TPMS structures) or strut diameter (for strut-based structures). These derived dimensions are then used as the target wall thickness or strut diameter for the transition cells. As a result, deviations in the relative density of transition cells may occur (e.g., Figure 2c). However, the trend of variation remains close to the maximum values in neighboring regions and can be adjusted using the proposed voxel-erosion method. Compensation was implemented in two steps. First, systematic errors inherent to the MATLAB algorithm used to generate printable files were corrected by analyzing topology-specific deviations. For example, if a target relative density of

0.30 produced an actual density of 0.29, a compensation factor Pcom = 0.30 / 0.29 was applied, and the corrected target value became 0.30 × Pcom (see Figure S10f of the supplementary document for details). For the G, D, P, B, S, and F topologies fabricated in this study, the compensation factors were 0.923, 0.860, 0.917, 0.950, 0.967, and 0.940, respectively.

Second, a methodological compensation step was implemented based on the analysis presented in Figure 4f, which relates the number of eroded or dilated voxels in transition unit cells to the relative density of the interval. Figure S7 of the supplementary document illustrates the adjusted relative density distribution across the transition cells. In practice, the target relative density can be approached iteratively via controlled voxel erosion or dilation. According to the analysis shown in Figure S10f of the supplementary document, the residual relative density error after the first compensation step is already within an acceptable range. Therefore, when strict accuracy in the relative density of transition unit cells is not required, the second compensation step may be omitted.

In addition, variations in the transition interval length were analyzed (Figure S10 in the supplementary document). Based on these results, the k-value used in this study ranged from 0.01 to 0.2, depending on the structural dimensions, ensuring that the transition region remained approximately at the scale of a single unit cell. The fabricated structures, therefore, closely matched the intended design specifications.

Using the methodology described above on a workstation equipped with an Intel Xeon Platinum 8175M processor (2.5 GHz) and 192 GB of RAM, the code generates a hybrid structure consisting of 7 × 7 × 7 unit cells in approximately 33 minutes. The relatively long runtime is due to the voxel-based modeling approach, which produces STL models with redundant triangular facets. Although this represents a current limitation and an opportunity for future optimization, the method nevertheless eliminates the impracticality of manually constructing such complex hybrid lattice models."

Remark 2.13. Video S1: Instead of "Speed is accelerated," report the actual playback speed (e.g., "4× real time") or the real-time duration and the acceleration factor.

We thank the reviewer for this helpful suggestion. We have revised the annotations in supplementary video S1 to clearly indicate the playback speed relative to real time.

Remark 2.14. The manuscript uses vague phrasing such as "unique mechanical characteristics" and "distinct advantages and limitations." Please specify what characteristics, advantages, and limitations you mean, and quantify where possible.

We agree with the reviewer to clarify those terminologies in the revised version of the manuscript as follows:

> *"Among them, triply periodic minimal surfaces (TPMS) represent a particularly important class of structures that form continuous, self-supporting networks with advantageous mechanical characteristics, including high specific stiffness and specific strength, as well as controllable anisotropy [6, 7] [8, 9]. TPMS sheet architectures also offer large surface-to-volume ratios [10], which is beneficial for applications involving surface-driven interactions, such as biomaterials designed to regulate cell adhesion and growth [11]. By contrast, strut-based lattices provide greater topological freedom, enabling the realization of unusual mechanical responses, including negative stiffness [12], negative Poisson's ratio [13-15], high flexibility [16], adaptability [17, 18], and tunable stiffness [19]. As a result, sheet-based and strut-based lattices offer complementary functional advantages."*

Remark 2.15. The introduction is overly long and repetitive (roughly three pages). Please streamline to reach the central contribution more quickly, reduce redundancy, and state precise research questions and advances.

We thank the reviewer for this helpful suggestion. We have streamlined and revised the Introduction to improve clarity and reduce redundancy. Specifically, repetitive background descriptions have been condensed, and the discussion has been reorganized to reach the central research motivation and contribution more directly.

Remark 2.16. Fig. 3: The arrangement of panels a–c is confusing. Clarify the logical sequence, improve alignment, and explain the colored lines and backgrounds. The inset organization appears cluttered; consider reformatting or simplifying to improve readability.

We thank the reviewer for their comments. We have revised the layout and presentation of Figure 3 to improve clarity and readability. Specifically, the arrangement of panels a-c has been reorganized to better reflect the logical sequence of the analysis, and the alignment of the panels has been adjusted for clearer visual comparison.

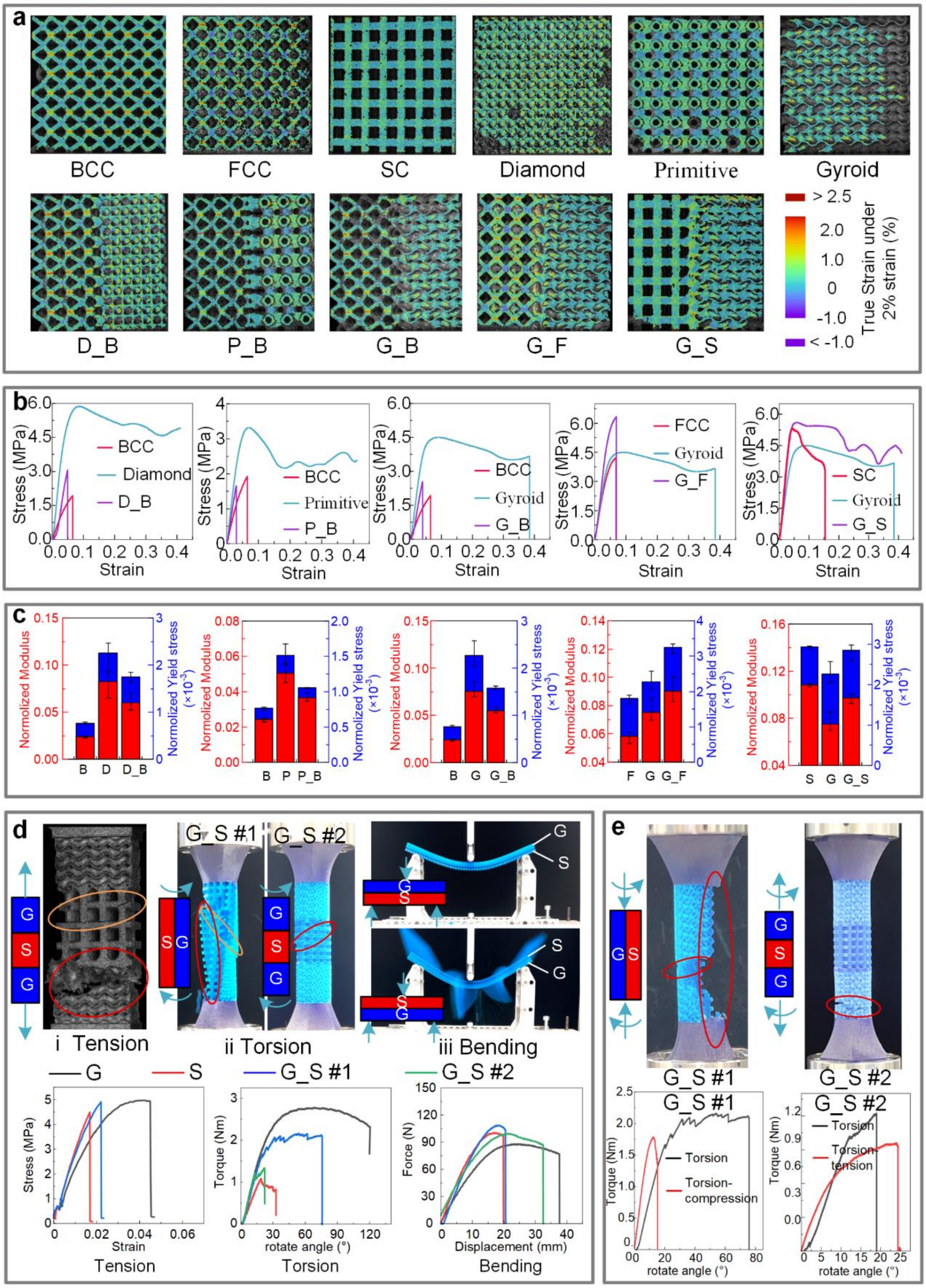

Remark 2.17. Ensure complete figure legends throughout (e.g., Fig. 5g appears to lack a legend). All symbols, colors, and abbreviations should be defined within each figure or caption.

We thank the reviewer for this careful observation. We have revised the figure captions

to ensure that all figures include complete, self-contained legends. Specifically, the missing legend information in Figure 5g has been added, and all symbols, color codes, and abbreviations used in the figures are now clearly defined within the corresponding figure captions.

[Figure Redacted]

Remark 2.18. The current configuration of the simulation is not demonstrably quasi-static and lacks essential controls. At 1000 mm/s, the response is likely rate-influenced unless quasi-static conditions are verified. Explicit dynamics with reduced integration (C3D8R) can suffer from hourglassing and artificial compliance without appropriate controls. Linear elasticity and NLGEOM off omit buckling and large-deformation effects typical of lattices, so reported stiffness/curves may only be valid at very small strains. Friction significantly influences compression results and requires calibration or sensitivity analysis. Please verify quasi-static conditions (e.g., kinetic-to-internal energy ratio, mass scaling disclosure), use hourglass control and report hourglass energy fractions; consider element types less prone to hourglassing (e.g., C3D8I) or

perform a mesh convergence study, and enable geometric nonlinearity where relevant and justify constitutive choices.

We thank the reviewer for this detailed and technically insightful comment regarding the numerical modeling setup. In the revised manuscript, we have carefully re-examined the simulation configuration and added additional analyses and clarifications. First, to verify that the simulations satisfy quasi-static conditions, we performed a loading-rate sensitivity analysis and evaluated the kinetic-to-internal energy ratio during the simulations. The results confirm that inertial effects remain negligible under the chosen loading conditions. This analysis has been added as Figure S12 of the supplementary document, with the corresponding explanation included in Section 4.5 of the revised manuscript.

Second, regarding potential hourglass effects associated with reduced-integration elements (C3D8R), we evaluated the hourglass energy contribution during the simulations and confirmed that it remains negligible relative to the internal energy. This verification is also reported in Figure S12 of the supplementary document and discussed in Section 4.5.

Third, regarding geometric nonlinearity and constitutive assumptions, the simulations in this study were intended to evaluate the elastic stiffness of the structures within the small-deformation regime. Therefore, the analysis focuses on the initial linear response, and the reported simulation results correspond to the compressive modulus extracted from the small-strain region rather than the full nonlinear deformation curve. This modeling choice has now been clarified in the manuscript.

Finally, regarding friction effects, the friction coefficient used in the contact definition was adopted from previously reported studies on similar lattice compression simulations (Yang et al., Materials & Design, 2023). Because the analysis focuses on the initial small-deformation regime, the influence of friction on the extracted stiffness values is expected to be limited. This assumption and the corresponding reference have now been explicitly stated in Section 4.5. All the revisions as follows:

> *"Each INP file contained meshing details and used C3D8R elements. Relevant analysis has been performed; the hourglassing effect was found to be minimal and can be neglected (see Figure S12b of the supplementary document for more details)"*
>
> *"set loading speed to 1000 mm/s (Relevant analysis confirms that quasi-static conditions were satisfied. Please refer to Supplementary Fig. S12a for details.)"*

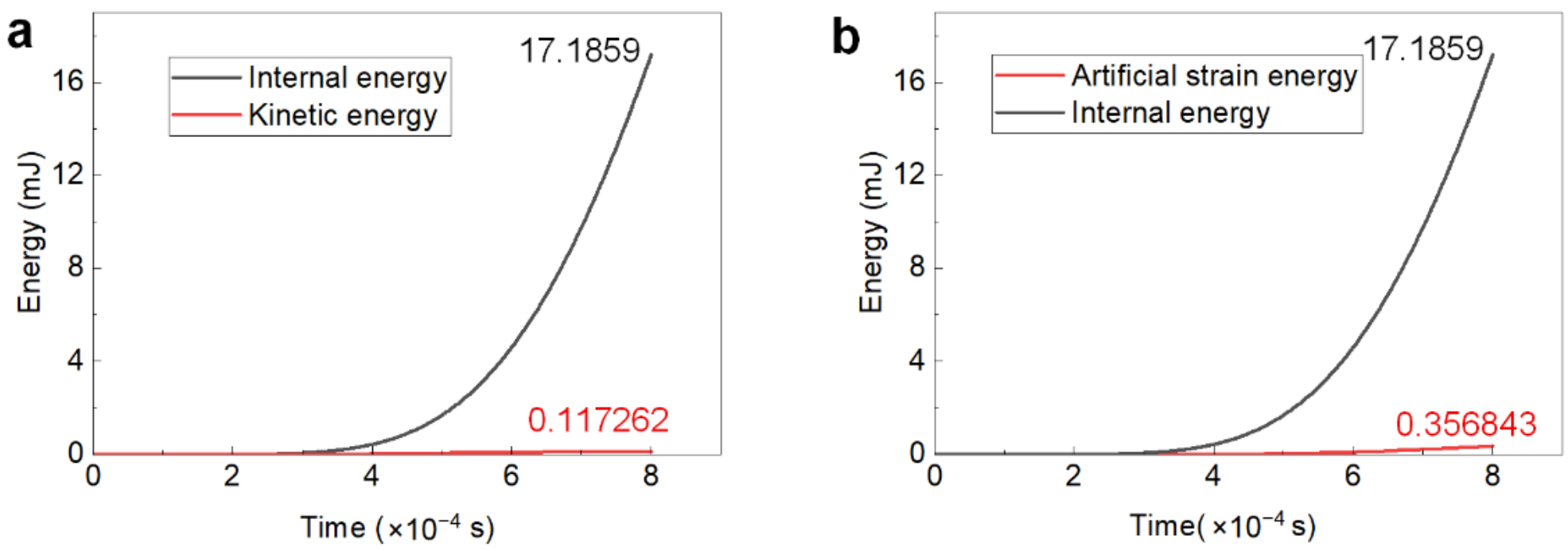

Figure S12. *The analysis of FEM setting: (a) Internal energy and kinetic energy for quasi-static analysis. (b) Internal energy and Artificial strain energy for hourglassing analysis.*

> *"Fig. S12 presents the analysis results of the History Output from the simulation of a BCC structure with a relative density of 20%. The ratio of the kinetic energy to internal energy is 0.68%, which is below the 5% threshold, confirming that the loading rate in the simulation is appropriately set and meets the requirements for quasi-static loading. Additionally, the ratio of artificial strain energy to internal energy is 2.08%, also below 5%, indicating that hourglassing effects have a negligible impact on the computational results and can be disregarded. In summary, this analysis demonstrates the feasibility of the simulation setup, its alignment with real-world experimental loading conditions, and the reliability and validity of the simulation results."*

Remark 2.19 Constituent structures are assigned relative densities, whereas transition structures are "generated automatically" without explicit target density, followed by unspecified "compensatory adjustments," with varying transition lengths. The manuscript also claims that fabricated structures match designs without presenting evidence. Please formally define how transition voxels are assigned and how relative density is controlled across the interface; report as-designed and as-built densities; and clarify how consistency is enforced so that mechanical property comparisons and scaling (e.g., modulus vs. density) are meaningful.

We agree that a clearer description of the transition voxel assignment and density control procedure is necessary to ensure reproducibility and meaningful mechanical comparisons.

In the revised manuscript, we have expanded the methodological description in Section 4.1 to formally define how transition voxels are generated and how the relative density

of the transition region is controlled. Specifically, transition unit cells are constructed based on the geometric parameters of the adjacent constituent structures, where the strut diameter or wall thickness of the transition cells is matched to those of the neighboring architectures to ensure geometric continuity across the interface.

Because this geometric matching can introduce small deviations in the relative density of transition cells, we implemented a relative-density compensation procedure within the voxel-based modeling framework. The detailed compensation methodology and the corresponding compensation factors used for the different lattice topologies are now explicitly described in Section 4.1 of the revised manuscript.

To further clarify the density distribution across the hybrid structures, we have also included additional analysis comparing the as-designed relative densities and the experimentally measured densities obtained from μCT reconstruction, as presented in Figure 2c and discussed in the Results section. These results demonstrate that the fabricated structures closely match the intended design and that the density variations across the transition regions remain controlled.

These revisions provide a clearer definition of the transition voxel assignment, the density control strategy, and the compensation procedure used to ensure consistency between the design and fabricated structures.

Point-by-point response to the reviewers' comments

Reviewer #1:

Remark. The authors have addressed all issues raised, thoroughly revising the manuscript and enhancing its quality. The work advances the state of the art in hybrid connections of porous lattice structures.

We thank Reviewer #1 for the positive assessment and for acknowledging the improvements made to our manuscript. We are grateful for the reviewer's time and constructive feedback throughout the review process.

Reviewer #2:

Remark. The reviewer appreciates the extensive effort that went into revising this manuscript, which has improved the work. However, because some of the original comments were fundamental in nature and inherently difficult to resolve within the current framework, several of the responses feel superficial or hypothetical, leaving key issues unaddressed.

For example, while the authors acknowledge that their initial claims regarding novelty and breadth were overly broad, they now repeatedly point to "biological attachment interfaces (e.g., tendon-bone connections)" as a primary use case "for scenarios that require abrupt functional transitions within limited spatial regions." While this is a valid and useful application, narrowing the scope to this specific domain significantly limits the breadth and general readership of the work.

Ultimately, this limitation stems from the underlying nature of the framework; not from a lack of effort by the authors. The reviewer believes the authors have addressed the comments as much as practically possible given these constraints.

We sincerely thank Reviewer #2 for recognizing the extensive effort we have put into the revision and for the thoughtful and fair assessment. We acknowledge the inherent limitations of the current framework, as the reviewer points out, and we appreciate the understanding that these constraints are structural rather than due to a lack of effort. We believe that the application examples presented demonstrate the value and utility of our approach within its current scope. We are grateful for the reviewer's constructive criticism, which has helped us refine our manuscript and better position our work.
