# Supplementary material for: Strategy for hybrid connection of porous strut and triply periodic minimal surface lattice structures
Source: Nat Commun. 2026 Oct 5;17:9699. doi: 10.1038/s41467-026-77560-7 (PMC13639051; doi:10.1038/s41467-026-77560-7)
Supplement: Supplementary file 2 [file 41467_2026_77560_MOESM1_ESM.pdf]

Supplementary Information for

Strategy for hybrid connection of porous strut and triply periodic minimal surface lattice structures

Jianxing Yang[1,2,3,*,#], Vahid Moosabeiki[2,#], Long Bai[3], Mauricio Cruz Saldívar[2], Marius A. Leeflang[2], Xiaohong Chen[3], Amir A. Zadpoor[2], Mohammad J. Mirzaali[2,*]

[1] College of Mechanical Engineering and Automation, Huaqiao University, Xiamen 361021, China

[2] Department of Biomechanical Engineering, Faculty of Mechanical Engineering, Delft University of Technology (TU Delft), Mekelweg 2, 2628 CD, Delft, The Netherlands

[3] College of Mechanical and Vehicle Engineering, Chongqing University, Chongqing 400044, China

[#] These authors contributed equally to this work.

[*]Corresponding authors: jianxingyang@hqu.edu.cn and M.J.Mirzaali@tudelft.nl

Supplementary Note 1. Geometrical accuracy and fabrication reliability of 3d-printed structures.

The geometrical characteristics of the 3D printed structures, as depicted in Figure 2 of main text and Supplementary Figure 1, closely align with the original design models, indicating high print quality. The relative densities of compression samples, as recorded in Supplementary Table 1, indicating high print accuracy and fabrication reliability. The relative density measurement of the compression specimens (Supplementary Table 1), show minor deviation from the design values. Specifically, the D structure exhibited higher actual relative densities than originally designed. The G structures closely approximated the intended design values. Other structures displayed slightly lower relative densities, consistent with the layer-by-layer relative densities trends observed in the μCT reconstruction models of the hybrid structure (Figure 2 in main text). These results confirm that the stability and reproducibility of the fabrication process across both hybrid and individual structures.

Supplementary Note 2. Analysis of strain distributions in individual structures.

The strain distributions measured by DIC during compression of each individual structure (Supplementary Figure 3) are presented both before and after significant damage (local or global). For the BCC and FCC lattice structures large strain concentrations were observed at the "×" node intersections. As compression increased, these structures suddenly failed, leading to catastrophic rupture that occurred too rapidly to be captured by the camera. Regarding the SC structures, initial high-strain regions appeared at the intersections of the cross-shaped nodes. As strain increased, vertical struts became the primary load-bearing elements, experiencing significant deformation. At 12.5% strain, localized buckling failure was evident, leading to stochastic layer-by-layer collapse. The G structures showed good toughness, enduring compression up to 22.6% strain without catastrophic failure. Instead of fracturing, the structure underwent large-scale deformation, primarily extruding at the bottom. High strains remained concentrated in the thinner sheet regions throughout the compression process. The D structures initially displayed high-strain zones with an inclined orientation, particularly in the thinner sections of the unit cells. As strain increased, it developed inclined shear cracks, indicating a progressive failure mechanism. Lastly, the P structures exhibited a layer-by-layer damage, similar to the G structures, indicating a gradual failure progression rather than sudden catastrophic collapse.

These results highlight distinct deformation mechanisms across different lattice structures, with strut-based architectures experiencing localized failures at key junctions, while TPMS sheet structures demonstrate enhanced toughness and gradual failure progression.

Supplementary Note 3. Analysis of strain distributions in five hybrid structures.

Supplementary Figure 4a depicts the deformation process and strain distributions of the five hybrid structures, during compression tests. In the low-strain region, the damage modes and strain distributions of the hybrid structures closely resembled those of their individual substructures under single loading conditions. This indicates that the hybrid structure connections did not interfere with the intrinsic mechanical behavior of the constituent architectures. All three hybrid structures incorporating the BCC lattice (i.e., G_B, D_B and P_B) experienced catastrophic failure, similar to the BCC structure alone, with sudden destruction occurring too rapidly to be captured by the camera. Post-failure observations of G_B and D_B structures (Supplementary Figure 4b) showed that the BCC section primarily failed through tilted shear collapse, which subsequently propagated through the entire structure. This failure pattern may be attributed to the limited number of unit cells within each section of the hybrid structure.

The hybrid structures incorporating FCC and SC lattices (*i.e.*, G_F, G_S) exhibited damage modes that were intermediate between those of their individual substructures. Despite structural failure, no fracture initiated at the connection transition regions, confirming that the proposed connection strategy effectively prevents stress concentrations at the interface.

These results validate the robustness of the hybrid structure design, ensuring smooth mechanical integration between different lattice types while preserving their intrinsic deformation characteristics.

Supplementary Note 4. Failure behavior and mechanical performance under different loading conditions.

Under bending, tension, and combined tension–torsion and compression–torsion loading conditions, failure of the representative hybrid structure G_S consistently occurred within the constituent substructural regions (indicated by red ellipses in Supplementary Figure 5a, c, e, and evidenced by the fracture streak in Supplementary Figure 5f due to rapid fracture), with no failure observed at the

connecting interfaces. This demonstrates the excellent failure resistance of the connection interfaces. Their load–deformation curves (Supplementary Figure 5b, d, and g) also reveal a favorable trade-off in the mechanical performance of the hybrid structures, such as achieving an optimal balance between load-bearing stiffness and fracture strain, thereby mitigating the traditional conflict between load-bearing capacity and fracture toughness in conventional structures.

Supplementary Note 5. FEM simulation results.

To evaluate the performance of additional hybrid structures with varying topology and relative densities, we conducted a series of finite element analyses (FEA). The strain distributions at 2% strain in both the FEA and digital image correlation (DIC) results exhibited similar trends, confirming their reliability (Supplementary Figure 7a). The FE results indicated that strain magnitudes in the transition zones were generally intermediate between those of the adjacent structures, except for the G_F hybrid structure, which exhibited higher strain concentrations.

Furthermore, a comparison between the normalized modulus obtained from FEA and experimental results revealed that although FEA predictions tend to be slightly higher, they follow a consistent trend (Supplementary Figure 7b). This demonstrates the validity of using FEA simulations to approximate structural stiffness modulation, reducing the need for extensive experimental testing while enabling efficient design exploration.

Supplementary Note 6. Normalized modulus stiffness tensor of transition unit cells.

In order to investigate the stiffness modulation capability of the transition unit cells, we analyzed the overall stiffness tensor distribution of hybrid structures (Supplementary Figure 8). This comparison shows that the stiffness distribution of transition unit cells is generally a fusion of the stiffness characteristics of the two constituent substructures.

When focusing on the two main loading directions of the hybrid structures, distinct trends emerge. In the x3-direction, the normalized modulus of the B_D and B_G transition unit cells was 0.0694 and 0.0600, respectively. In the x_2-direction, the normalized modulus was 0.0434 and 0.0478, respectively. These

values fall between the corresponding elastic modulus values of the BCC structure (0.0327) and the D structure (0.1368) or G structure (0.1154), indicating a gradual transition in elastic moduli, effectively bridging the mechanical properties of the two constituent substructures.

For the B_P hybrid structure, the stiffness of the transition cells was significantly lower than that of both constituent substructures, with normalized modulus values of 0.0201 and 0.0003. This suggests a weakened zone, which could potentially lead to stress concentrations or premature failure under loading.

The F_G hybrid structure exhibits the opposite behavior, with transition unit cells possessing higher elastic modulus than both of their constituent structures. The normalized modulus in the x_3- and x_2-directions was 0.3363 and 0.1779, respectively, which are higher than those of the FCC (0.0951) and G (0.1154) structures.

The transition unit cell of the S_G hybrid structure follow a similar trend to the F_G hybrid structure but with a mixed behavior. While the x_3-direction modulus remained higher, the x_2-direction modulus (0.0946) was slightly lower than one of the constituent structures (S = 0.1516, G = 0.1154), though still within a comparable range.

Hybrid structures where the stiffness of the transition unit cells is intermediate to that of the constituent substructures are often preferred, as they facilitate efficient force transfer while minimizing stress concentrations. In this regard, transition unit cells with higher elastic modulus, prevent premature damage at the connection points. They might also hinder force transmission, potentially leading to localized stress buildup in other regions. The transition unit cells with a lower elastic modulus, can increase the risk of failure at the transition interface if they are too weak. However, the failure behavior can also be influenced by additional mechanical factors, including load distribution, geometric variations, and materials properties.

These results emphasize the importance of careful stiffness modulation in hybrid lattice structures, ensuring that transition regions effectively balance mechanical compatibility, load transfer efficiency, and structural integrity.

Supplementary Note 7. Relative density regulation of transitional unit cells.

Supplementary Figure 9 demonstrates the regulation of relative density in transitional unit cells. When reducing relative density, the erosion method is employed, as illustrated in (a) for the G_F transitional cell. Here, the notation "XV" (where X is a numerical value) indicates erosion to a wall thickness of X voxels. Conversely, when increasing relative density, the dilation method is used, as shown in (b) for the P_B transitional cell, where "XV" signifies dilation by X voxels in wall thickness. All operations are performed while preserving the connectivity of the boundary interfaces. Figures (c) and (d) present the relative density curves obtained under different wall-thickness adjustments, demonstrating effective and precise control over the relative density.

Supplementary Note 8. The stiffness regulation results for hybrid structures.

To facilitate an efficient prediction of hybrid structural stiffness, we established a fitting model for the normalized modulus based on different voxel erosion and voxel dilation operations. The relationship between the relative density (ρ) of the transition unit cell and the number of retained (n_{s}) or dilated (n_{a}) was first determined as follows:

$$\rho = A_{\mathrm{s}} n_{\mathrm{s}} + A_{\mathrm{a}} n_{\mathrm{a}} \tag{1}$$

, where A_{s} and A_{a} were linear constants related to the topology of the structure. When the erosion operation is performed, A_{a} equals 0; when the dilation operation is carried out, A_{s} equals 0.

For voxel erosion, the number of retained wall-thickness voxels served as a controlling variable. Since structural topology remained unchanged, a single voxel corresponded to a “layer thickness”, meaning that a adding or removing voxels resulted in a fixed-volume change. A similar principle applied to voxel dilation, where increasing the number of voxels corresponded to the addition of a new topological layers, thereby modifying the transition unit cells’ stiffness.

As per the Gibson-Ashby [1] model, the structural stiffness (E) follows a power-law relationship with the relative density (ρ). Substituting this into Equation 1, the normalized modulus (E/E^*) of the transition unit cell structure as a function of voxels is given by:

$$\begin{cases} \frac{E}{E^*} = C_\mathrm{s}(n_\mathrm{s})^{m_\mathrm{s}} \\ \frac{E}{E^*} = C_\mathrm{a}(n_\mathrm{a})^{m_\mathrm{a}} \end{cases} \quad (2)$$

, where C_s, C_a, m_s, and m_a are coefficients dependent on the structural topology and material properties. The normalized modulus in the x_3-direction for the G_F and P_B transition unit cells was fitted using Equation 2, with the results depicted in Supplementary Figures 10a and 10b.

To further validate the proposed stiffness modulation strategy, we constructed G_F and P_B hybrid structures with varied transition unit cell voxel configurations, and performed finite element simulations to computed their normalized compressive modulus. These results were then compared with theoretical predictions derived from the rule of mixtures. In the G_F hybrid structure, as the number of retained voxels decreased, the overall stiffness of the structure progressively declined. Comparing the original structure to a 6-voxel-thick retained wall, both simulation and theoretical predictions showed a normalized modulus adjustment of 0.0287 and 0.0281, respectively. The simulation and theoretical results showed close agreement, confirming the validity of the model (Supplementary Figure 10c). In the P_B hybrid structure, as the number of dilated voxels increased, the overall normalized modulus of the structure increased correspondingly. When the transition unit cell dilated to a 6-voxel wall thickness, the simulation and theoretical calculations indicated stiffness adjustment of 0.0276 and 0.0115, respectively (Supplementary Figure 10d). However, at higher dilation levels, a significant deviation emerged between simulation and theoretical results. This discrepancy is likely due to the progressive reduction in wall spacing, which enhanced structural stiffness as neighboring walls begin to interact and make contact under increasing strain. In contrast, theoretical calculation assume small-stain behavior, failing to account for this effect.

Supplementary Note 9. Principle of voxel analysis for generating 3D-printed hybrid structures.

Hybrid structure generation process. To generate the hybrid structure, we integrated TPMS structure functions with a weight transition function, replacing strut-based substructure through the following steps: 1. Define the target region using a TPMS structure function. 2. Replace the strut-based structure with a corresponding TPMS skeletal structure, following the methodology detailed below. 3. Generate a pure

strut-based structure in the designated region, following [2]. 4. Determine which structure should be replaced. In this case not all substructure regions were fully substituted, as the transition structure from TPMS sheet to the skeletal region must be retained. For this purpose, a logical matrix selection process was used to identify unit cells that fully belong to the strut-based domain. If a unit cell was entirely within the strut-based domain, it was replaced by a strut-based structure. If a unit cell was in an intersection region, the transition structure was retained.

Generating hybrid structures from TPMS functions. Since TPMS structures can be defined by continuous mathematical functions, the entire hybrid structure can be generated using a single equation, integrating the regional structure functions through a weight transition function, as formulated in Equations 4-6 of the main text.

A key aspect of this approach is computing the weight function, which was achieved using control point coordinates: 1. Transform the spatial voxel grid into scatter points. 2. Perform an image erosion operation to refine the control points. If the design domain includes a global boundary, the erosion moves toward the boundary (Supplementary Figure 12a). If the design domain is enclosed, erosion progresses toward the geometric center (Supplementary Figure 12b). 3. Maintain consistent erosion depths across different domains to avoid boundary misalignment. 4. Substitute the control point coordinates into Equation 6 of the main text to compute the weight function.

This method ensures that TPMS hybrid structures are generated with seamless connections, eliminating discontinuities in one processing step.

Voxel-based design domain definition. For a given i-th design domain (numbered 1–i): If the domain contains a TPMS structure, it is expressed by the function $\Phi_i(x,y,z)$, where voxels satisfying $\Phi_i > t_i$ constitute the TPMS structure. The relative density (ρ) is controlled by t_i. In which, a constant t_i yields a homogeneous TPMS structure, and a variable $t_i(x,y,z)$ enables a gradient-density transition.

Voxel matrix representation and indexing. The voxelization process is illustrated in Supplementary Figures 12c-e with the following steps: 1. Define voxel size and subdivide the design domain into a finite number of voxels. 2. Assign voxel coordinates in 3D space, using a rectangular Cartesian grid. The first voxel center in each direction is positioned at half a voxel's side length. The indexing system follows a

layered pattern (Supplementary Figure 12d) which means starting from the origin, voxel indices are assigned along the x-axis, then incremented along the y-axis, and finally along the z-axis. 3. Store voxel data. Each voxel's index and center coordinates are computed and saved. Voxels satisfying $\Phi_i(x, y, z) > t_i$ are assigned 1 (white in Supplementary Figure 12d), while others are assigned 0 (black in Supplementary Figure 12d). 4. Logical matrix representation (Supplementary Figure 12e). For example, a P-surface sheet unit cell is represented with a 40-voxel resolution, which can be adjusted for precision control. For strut-based structures, models were defined using a wireframe representation, following the method outlined in literature [2], since continuous mathematical expressions are not practical for these geometries.

Finalizing the hybrid structure for fabrication. After generating the voxel-based hybrid structure, the voxel matrices must be converted into .STL files for direct 3D printing. This was accomplished through: 1. Marching cubes algorithm [3, 4] which converts the voxel matrix into a polyhedral surface representation composed of triangular facets. 2. Surface smoothing function [5] which optimizes the triangulated surface, reducing artifacts and ensuring a smooth final model.

This process ensures that the hybrid structure is both computationally efficient and fabrication-ready, allowing for seamless integration into additive manufacturing workflows.

Supplementary Note 10. The effect of the relevant parameters on the geometrical properties of the generated hybrid structures.

To investigate the effect of resolution on structure generation, the G_B hybrid structure was used as a case study. Structures with a fixed relative density of 25% were generated at resolutions of 40, 60, 80, and 100, and the resulting .STL files were imported into Materialise Magics 22.0 (Materialise NV, Leuven, Belgium) for analysis. The software was used to evaluate connectivity, dimensional accuracy, and volume of the generated models.

As shown in Supplementary Figure 13a, increasing the resolution led to a progressive reduction in both relative density deviations and dimensional errors. At resolution = 100, the relative density deviation was

only 0.33%, and the maximum dimensional deviation was as low as 0.0099 mm, demonstrating excellent accuracy. However, topology continuity issues were evident at lower resolutions. At resolution = 40, a disconnection was observed at the heterogeneous connection region. Increasing the resolution to 60 resulted in a thin-wall connection, though some discontinuities remained. With further increases in resolution, gradual improvements in connectivity were observed. While higher resolutions enhance geometrical accuracy, they also significantly increase computational costs. Considering model generation efficiency, fabrication constraints, and printer accuracy, a resolution of 80 was selected for physical sample manufacturing in this study.

In addition to resolution, the transition parameter k in Equation 5 of main text plays a critical role in determining the transition interval length and connection smoothness. To determine an optimal k value, G_B hybrid structures were generated at $k = 10^{-2}, 10^{-1}, 10^{0}, and\ 10^{1}$, while keeping all other parameters constant, including resolution ($n = 60$). As depicted in Supplementary Figure 13b, increasing k resulted in a shorter transition interval. At $k = 10^{-2}$, the transition interval spanned nearly the entire unit cell, deviating from the intended design. In contrast, at $k = 10^{-1}$, the transition interval closely matched the length of a unit cell, aligning well with the design expectations and ensuring a smooth transition between lattice structures.

Further analysis revealed that k did not significantly affect size deviations, but it did influence relative density deviations. As k increased, the relative density deviation gradually increased. However, this trend was not absolute, as it also depended on factors such as resolution and the percentage of unit cells within the transition region. The observed pattern is attributed to the fact that, at the selected resolution, G structures exhibited a higher-than-expected relative density. As k increased, the transition interval length decreased, meaning that a larger portion of the structure was composed of G regions with inherent density deviations, leading to an increase in the overall relative density deviation.

Based on our results in Supplementary Figure 13b and the relative density deviation analysis, a transition interval of one unit cell length was considered optimal, providing an effective balance between connection smoothness and dimensional accuracy. Since the relative density deviations remained within an acceptable range, $k = 10^{-1}$ was selected as the most suitable value for this study. This ensures that hybrid structures maintain high geometrical accuracy, while minimizing fabrication inconsistencies and

transition-related discontinuities.

Since this study utilized control points to delineate different structural topological zones, their distribution and quantity directly influenced the calculation of the weight function. These control points were generated based on predefined boundary rules, meaning that variations in their number and spatial distribution affected the overall transition structure formation. The primary factors influencing control point generation were the proportion of the transition structure within the total design domain and the size of the individual unit cells. As illustrated in Supplementary Figures 13c-f, these two parameters played a crucial role in determining the transition interval and overall structural continuity.

Supplementary Figure 13c compares the generation of transition regions at varying transition structure proportions while keeping the transition parameter constant ($k = 0.1$). The notation A_B_C represents the number of unit cells in the horizontal direction of the first single structure (G structure, A), the transition structure (B), and the second single structure (BCC structure, C), respectively. For example, in the 3_1_3 structure (first row of Supplementary Figure 13c), the transition region occupies 1/7 of the total structure. Here, all transition regions were assumed to be one unit cell in length, and the final selection of k was aimed at ensuring that the transition structure remained within this length. However, as the number of single-structure unit cells increased, the transition region length decreased accordingly. Moving from top to bottom in the figure, as the number of single-structure unit cells increases from 3 to 10, the length of the transition region (transparent red area) progressively shortens. This implies that if the transition interval is intended to match the length of a single unit cell, a lower k value should be selected as the number of unit cells increases.

To determine an appropriate k value, Supplementary Figure 13d shows how k values ranging from 0.01 to 0.1 influence the transition interval when the number of single-structure unit cells is 10. It was observed that a transition interval approximately equal to the length of a single unit cell was obtained at $k = 0.05$. The unit cells in these comparisons had side lengths of 2 mm, but Supplementary Figure 13e extends this analysis by scaling the unit cell size by a factor of three (to 6 mm) and examining k values between 0.005 and 0.1. The results show that a suitable transition interval matching one unit cell length was achieved at $k = 0.01$.

In this study, relative density control in hybrid structure generation was applied only to each individual

structural region, while the transition region was automatically generated based on the substructures of the neighboring regions. As a result, the relative density of the transition region was not explicitly controlled. To demonstrate that this lack of direct control has only a minor effect on the overall structure's relative density, a statistical analysis was conducted on the relative density deviation for structures with different transition region proportions (Supplementary Figure 13f- left).

As previously mentioned, the relative density deviation of the G structure at a target relative density of 25% consistently exhibited an upward trend across the resolution range ($n = 40 - 100$). Since the relative density of the transition region was lower than that of the G structure, the overall deviation of the G_B hybrid structure increased as the proportion of the G structure increased. However, this deviation never exceeded the error observed in the single G structure itself. Conversely, in the D_B hybrid structure, the transition region typically had a higher relative density deviation than the single-structure regions. Therefore, as the transition region's proportion decreased, the overall relative density deviation also decreased. This trend suggests that for large-scale structures, the transition region occupies a smaller proportion of the total volume, making its impact on overall relative density control less significant. Consequently, focusing relative density control solely on the individual structural regions in the design process remains an effective approach.

The observed relative density deviations come from two primary sources. The first source of error occurred during the conversion of the logical matrix into an .STL file, which is a limitation of algorithmic accuracy in the equivalent surface model. This error originates from the MATLAB algorithm used to generate printable files and cannot currently be improved. However, since this error remains consistent and predictable, it can be mitigated through error compensation. Specifically, by analyzing the variation in deviations at different relative densities, targeted adjustments can be made. In this study, printed specimens were compensated accordingly, and the relative density deviations for three example structures with varying transition regions are presented on the right side of Supplementary Figure 13f. After compensation, the maximum relative density deviation was reduced to 0.03418, a significant improvement from the pre-compensation maximum deviation of 0.07815. The remaining error was primarily attributed to the second source of deviation.

The second source of error arose from the transition region's structural formation, which was generated

based on the wall thickness or strut diameter of the adjacent structures to ensure seamless connectivity. Since the relative density of the transition region was influenced by the geometric constraints of its neighboring structures, it did not always match their target densities. However, this deviation decreased as the proportion of the transition region decreased. For example, in the 10_1_10 structure, the highest relative density deviation was only 0.00754, a significant reduction compared to the 3_1_3 structure. Moreover, in cases where transition unit cell occupancy was low, such as in the 3_1_3 and 4_1_4 G_B structures, the relative density deviation was negative. This occurred because the transition region had a lower density than the target density, ensuring compatibility with the rod diameter or wall thickness of neighboring structures. As the occupancy of the transition unit cells decreased, the deviation gradually shifted to a positive value, which remained lower than the relative density deviation of the single structures.

These results confirm that although the relative density of the transition region is not explicitly controlled, its impact on the overall structure remains minimal, particularly in large-scale designs. By implementing error compensation and optimizing transition region proportions, high accuracy in relative density control can still be achieved within hybrid lattice structures.

As shown in Supplementary Figure 13a, disconnections and minimum dimensions were observed in the transition unit cells at heterogeneous joints, which can lead to localized stress concentrations and serve as fracture initiation points under loading. Although increasing the resolution partially mitigates these issues, it does not provide a fundamental solution. To address this, an optimized structure generation algorithm was developed by adapting methods from literature [6], incorporating object- and code-level improvements tailored to the framework used in this study.

A key enhancement in this optimization was the introduction of a transition unit cell control factor (β), modifying Equation 4 in main test to the following Equation 3:

$$\phi = \alpha_1\big(\phi_1 + \beta(1-\alpha_1)\big) + \alpha_2\big(\phi_2 + \beta(1-\alpha_2)\big) + \ldots + \alpha_n\big(\phi_n + \beta(1-\alpha_n)\big) \tag{3}$$

The introduction of β allows for precise control over transition unit cell thickness, with increasing β values leading to thicker transition regions, thereby reducing discontinuities and minimum feature sizes. However, this modification also introduces trade-offs. As β increases, the relative density deviation of the structure grows, and in extreme cases, the transition structure may lose its porous characteristics due

to excessive material accumulation. Since the method functions by adding material within the transition region based on the modified function, selecting an appropriate β value is crucial to achieving an optimal balance between structural connectivity, density accuracy, and mechanical performance. Based on iterative debugging results, an optimal value of $\beta = 0.5$ was chosen for this study.

The effectiveness of this optimization is demonstrated in Supplementary Figure 13g, which compares the transition zone details of the G_B hybrid structure before and after optimization. At resolution $n = 40$, the optimized structure successfully eliminated previously unconnected regions, ensuring continuous load transfer across the interface. At $n = 80$, the optimization led to thicker and more robust connections, particularly in the dashed box region of the figure. These enhancements significantly improved the mechanical integrity of the transition zone, mitigating stress concentration issues and ultimately enhancing the structural performance of the hybrid lattice.

Supplementary Note 11. The analysis of FEM setting.

Supplementary Figure 15 presents the analysis results of the History Output from the simulation of a BCC structure with a relative density of 20%. The ratio of the kinetic energy to internal energy is 0.68%, which is below the 5% threshold, confirming that the loading rate in the simulation is appropriately set and meets the requirements for quasi-static loading. Additionally, the ratio of artificial strain energy to internal energy is 2.08%, also below 5%, indicating that hourglassing effects have a negligible impact on the computational results and can be disregarded. In summary, this analysis demonstrates the feasibility of the simulation setup, its alignment with real-world experimental loading conditions, and the reliability and validity of the simulation results.

Supplementary Table 1. The comparison of relative densities (*i.e.*, RD) of the CAD models and 3D printed lattice structures of different topologies measured using the dry weight method.

Structure	CAD RD	Actual mean RD	Relative standard deviation of actual RD
B	0.2986	0.2621	0.00125
S	0.2985	0.2662	0.00053
F	0.3028	0.2644	0.00157
G	0.3019	0.2965	0.00768
D	0.3012	0.3403	0.00365
P	0.3019	0.2673	0.00374
G_B	0.3053	0.2777	0.00135
G_S	0.3211	0.3049	0.00478
G_F	0.3331	0.3122	0.00289
D_B	0.3059	0.3135	0.01026
P_B	0.2941	0.2529	0.00105

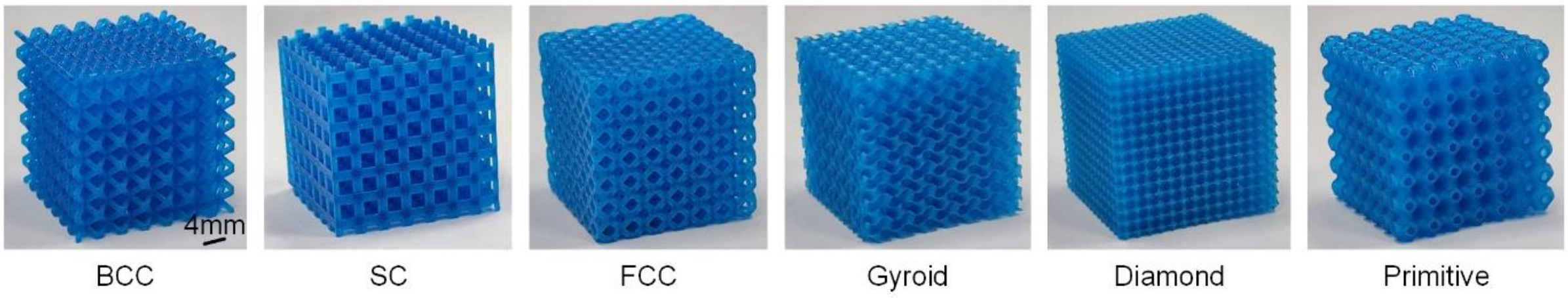

Supplementary Figure 1. Various single lattice structures made from strut- and sheet-based topology fabricated using polyjet 3D printing process.

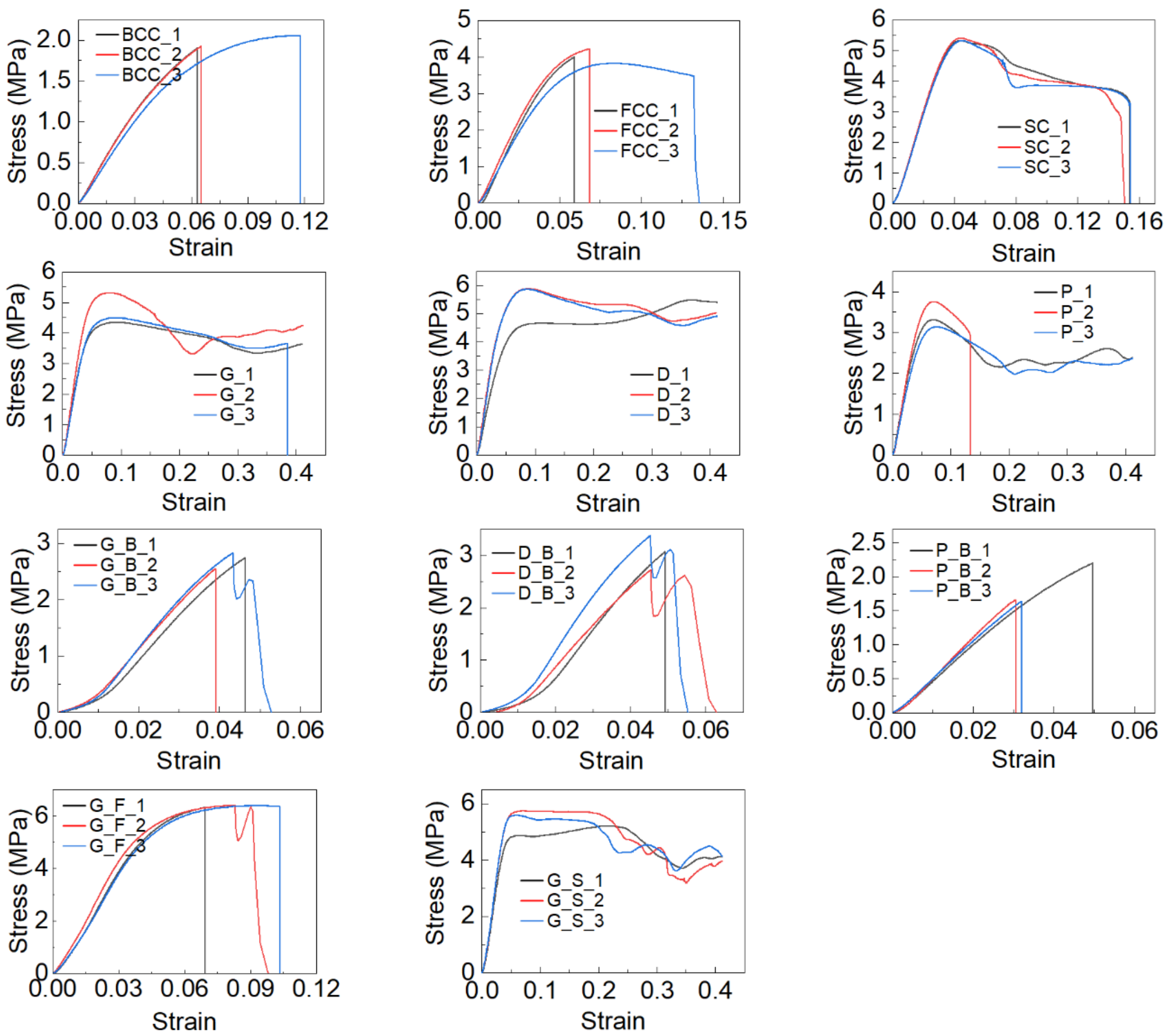

Supplementary Figure 2. The stress-strain curves obtained from repeated quasi-static uniaxial compression tests of different single and hybrid lattice structures. For curves where post-failure data were not recorded by the testing machine, the final data point at fracture was manually set to zero. This addition is made for visual clarity and does not represent measured data beyond the point of structural collapse. Source data are provided as a Source Data file.

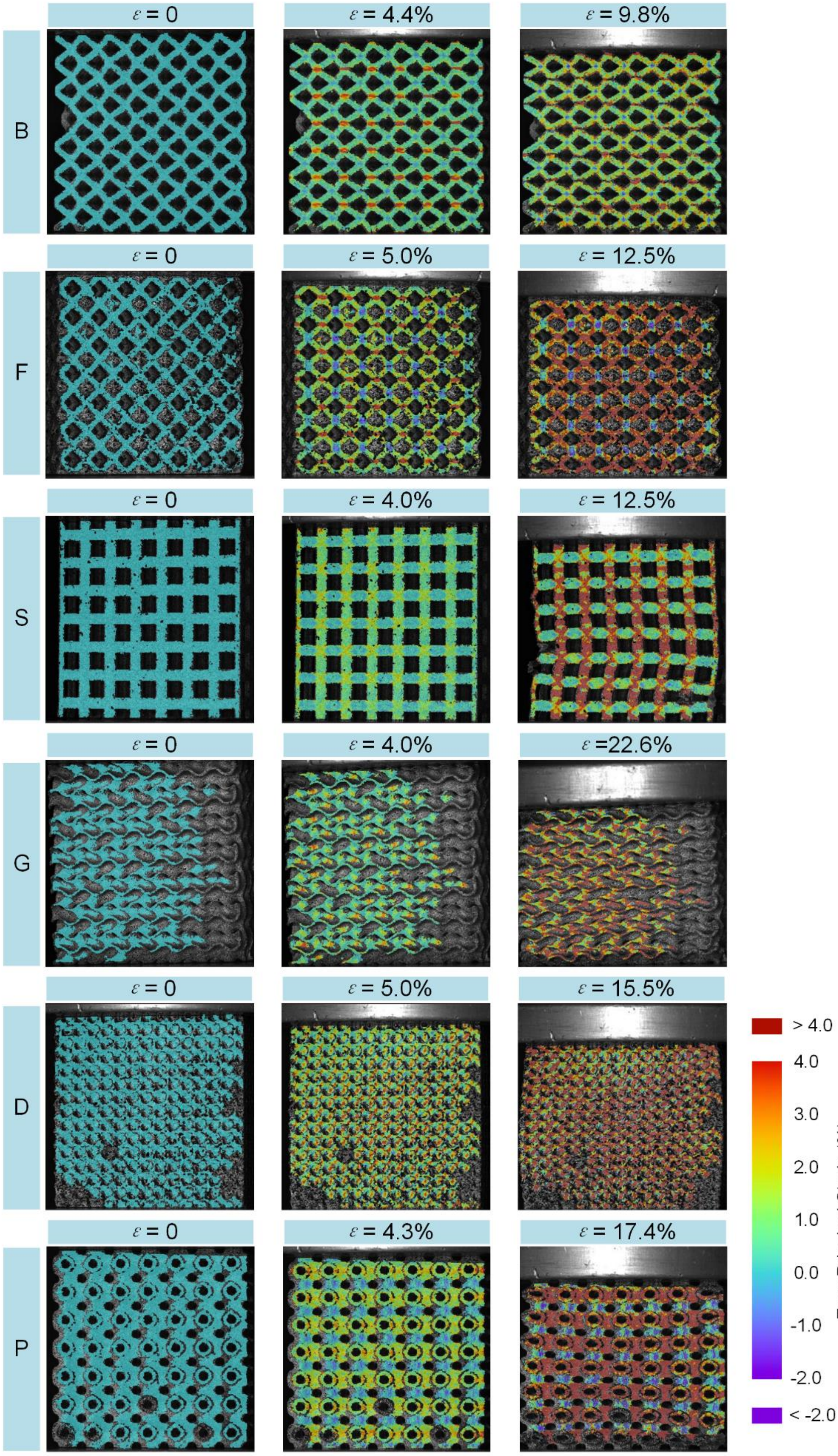

Supplementary Figure 3. The full-field strain (true principal strain) distribution of different strut-based and sheet-based lattice structures under different level of applied compression deformation.

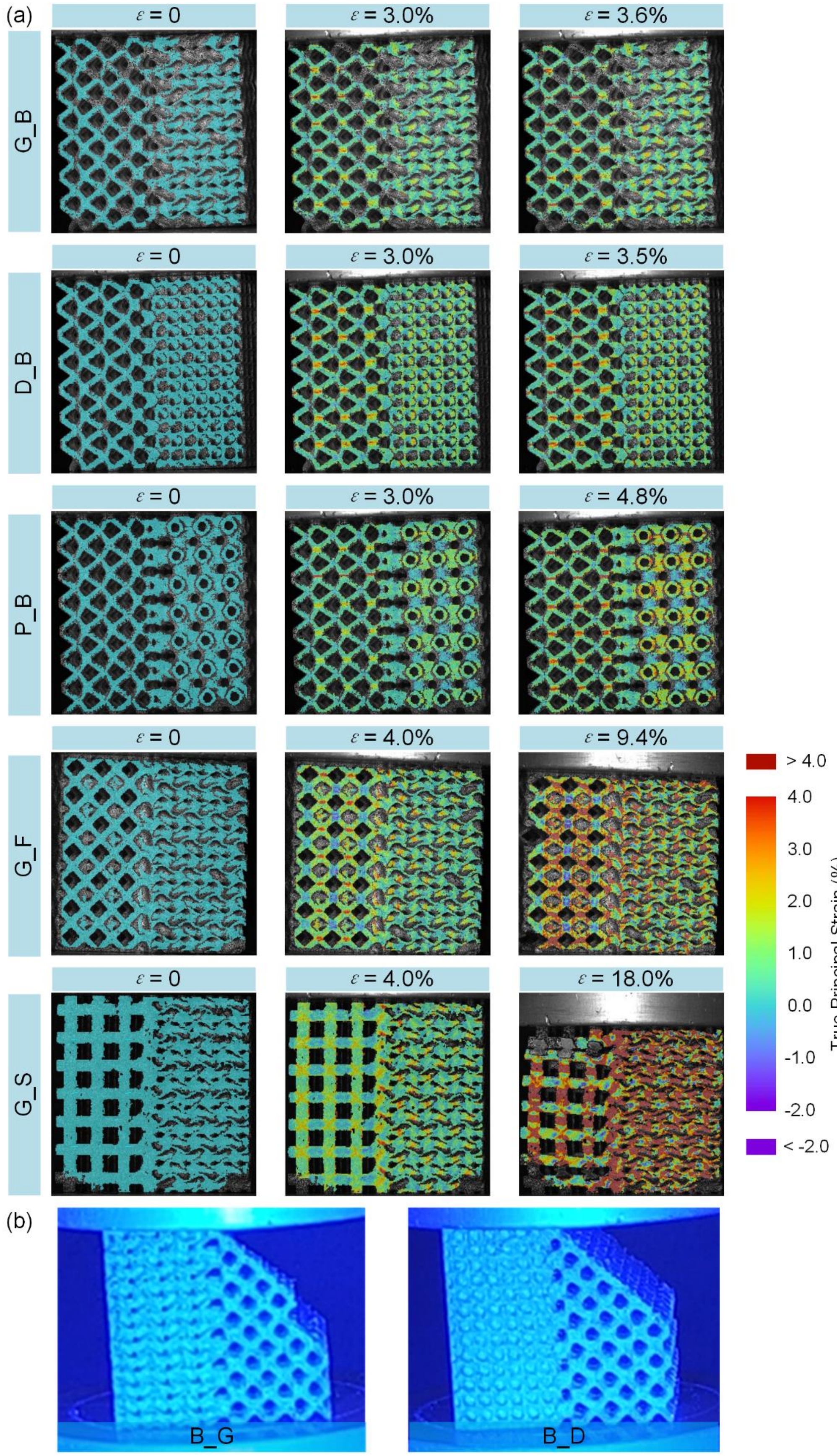

Supplementary Figure 4. (a) The full-field strain (true principal strain) distribution of different hybrid lattice structures. (b) The snapshots of the G_B and D_B hybrid structures after failure.

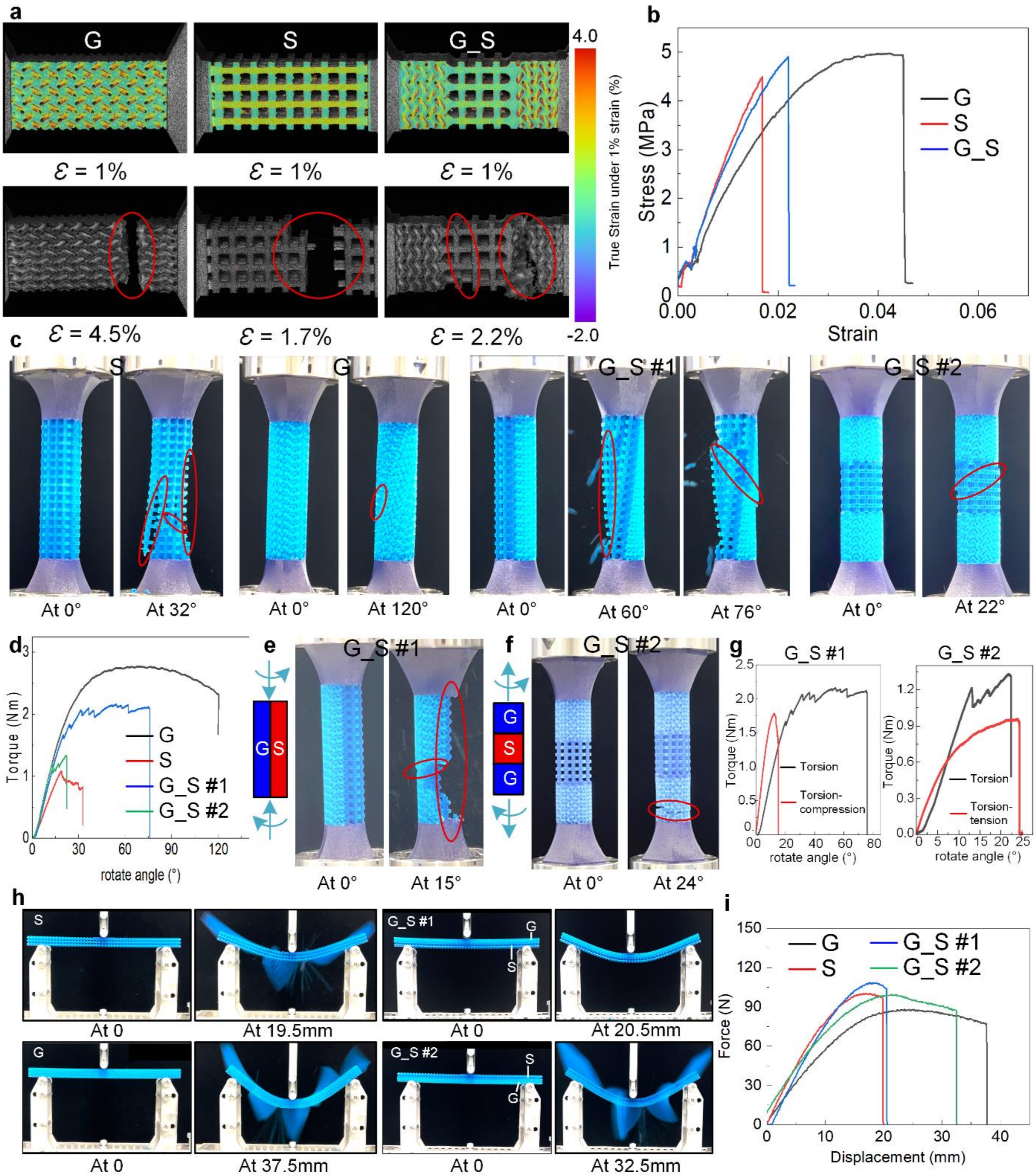

Supplementary Figure 5. Deformation response snapshots of the G, S, and their constituent hybrid structures under various loading conditions: (a) tensile condition, (c) torsional condition, (e) biaxial tension–torsion condition, and (f) biaxial compression–torsion condition. Their corresponding torque/stress/force *vs.* angle/strain/displacement curves: (b) tensile condition, (d) torsional condition, (g) torsional condition and biaxial conditions, and (i) bending condition. The red circles mark the fracture locations. Source data are provided as a Source Data file.

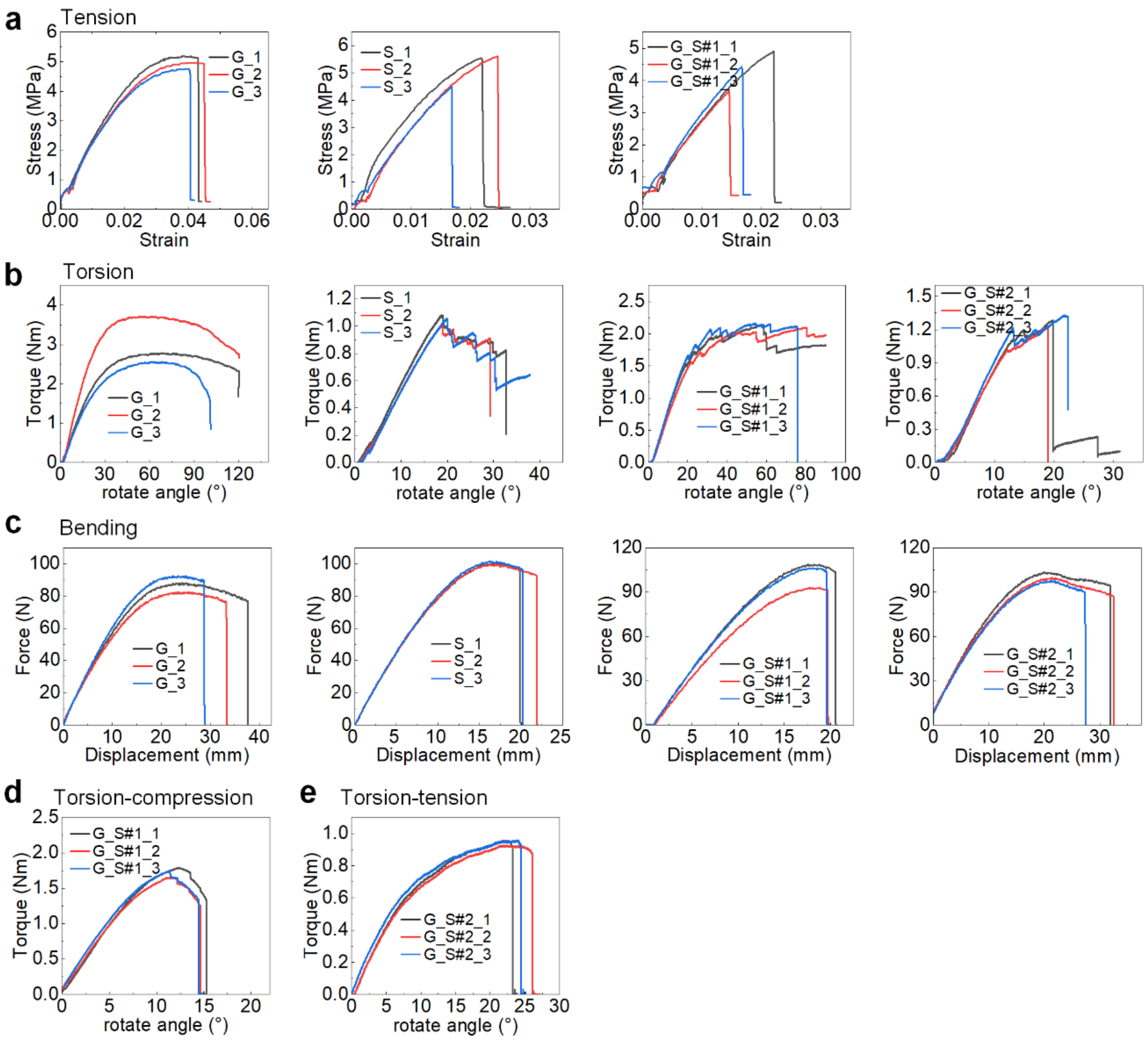

Supplementary Figure 6. The mechanical response curves obtained from repeated tests under various loading conditions: (a) tensile condition, (b) torsional condition, (c) bending condition, (d) biaxial compression–torsion condition, and (e) biaxial tension–torsion condition. Source data are provided as a Source Data file.

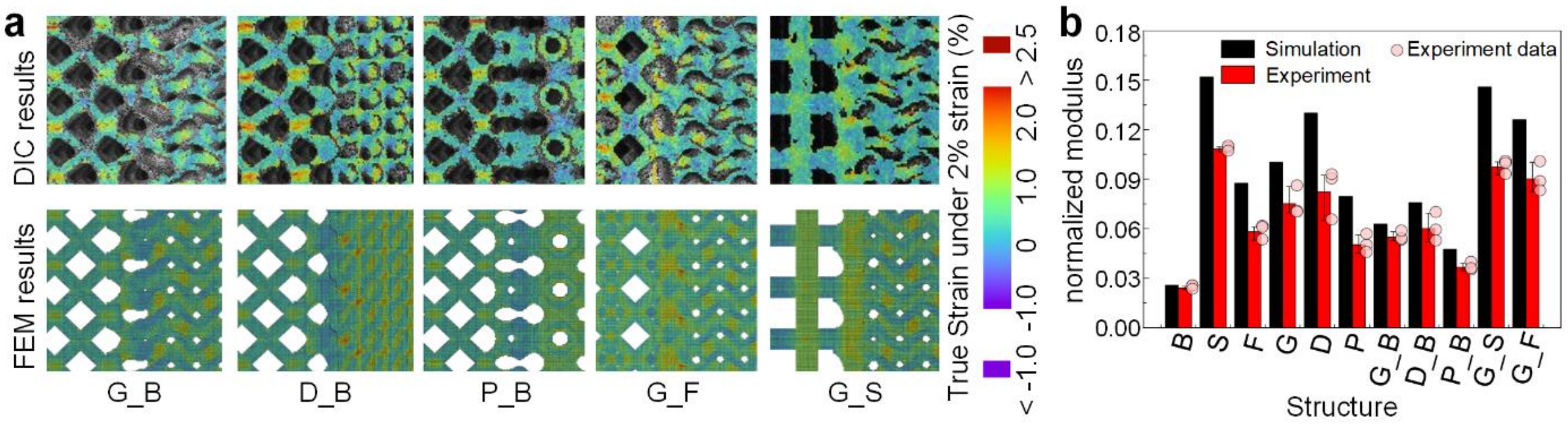

Supplementary Figure 7. FEM simulation results: (a) hybrid structures strain distributions under 2% strain of FEM simulation and DIC analysis; (b) Simulation and experimental comparison of normalized modulus for each structure. Source data are provided as a Source Data file.

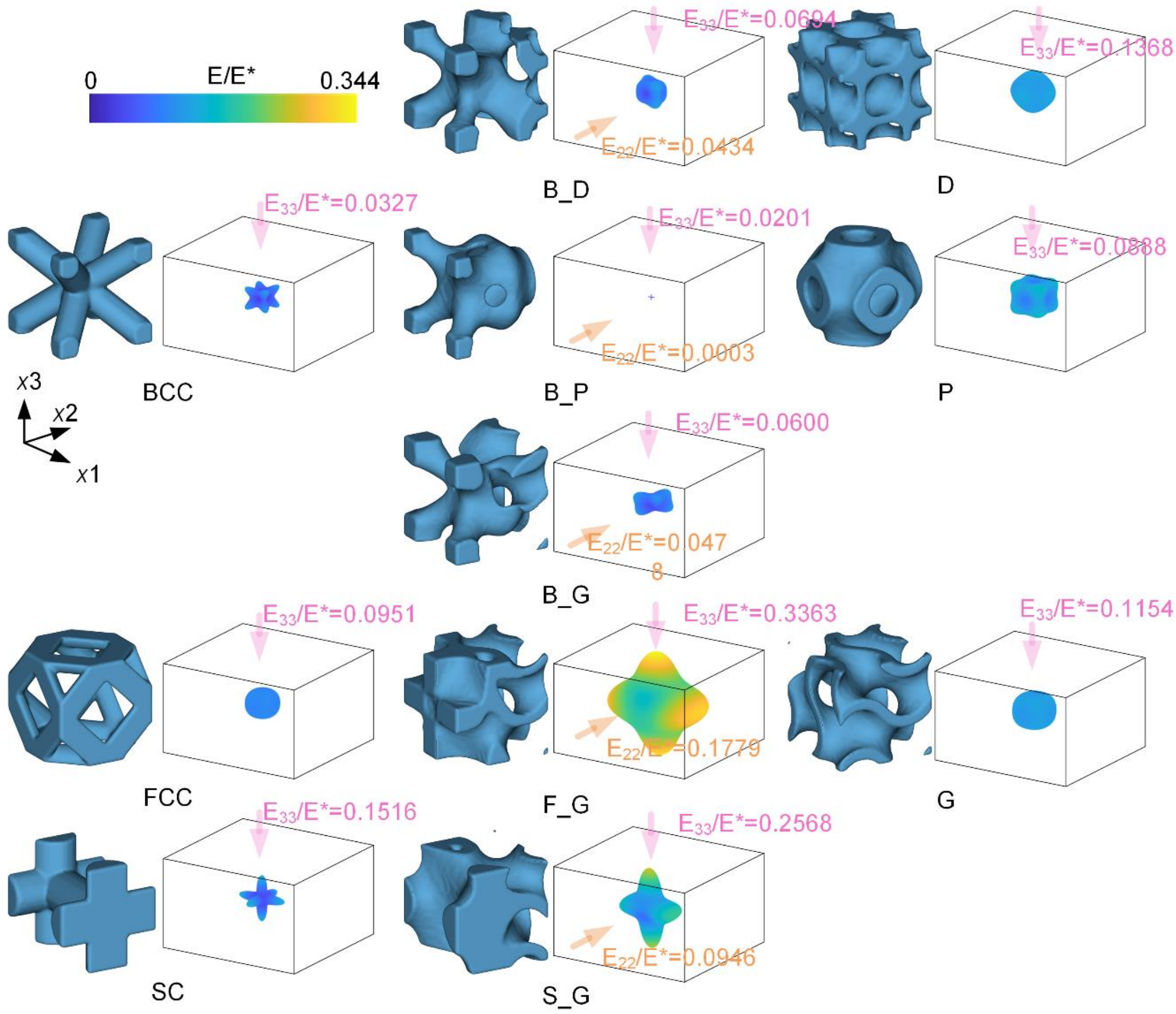

Supplementary Figure 8. Normalized modulus stiffness tensor of strut-based and sheet-based lattices and their corresponding transition unit cells.

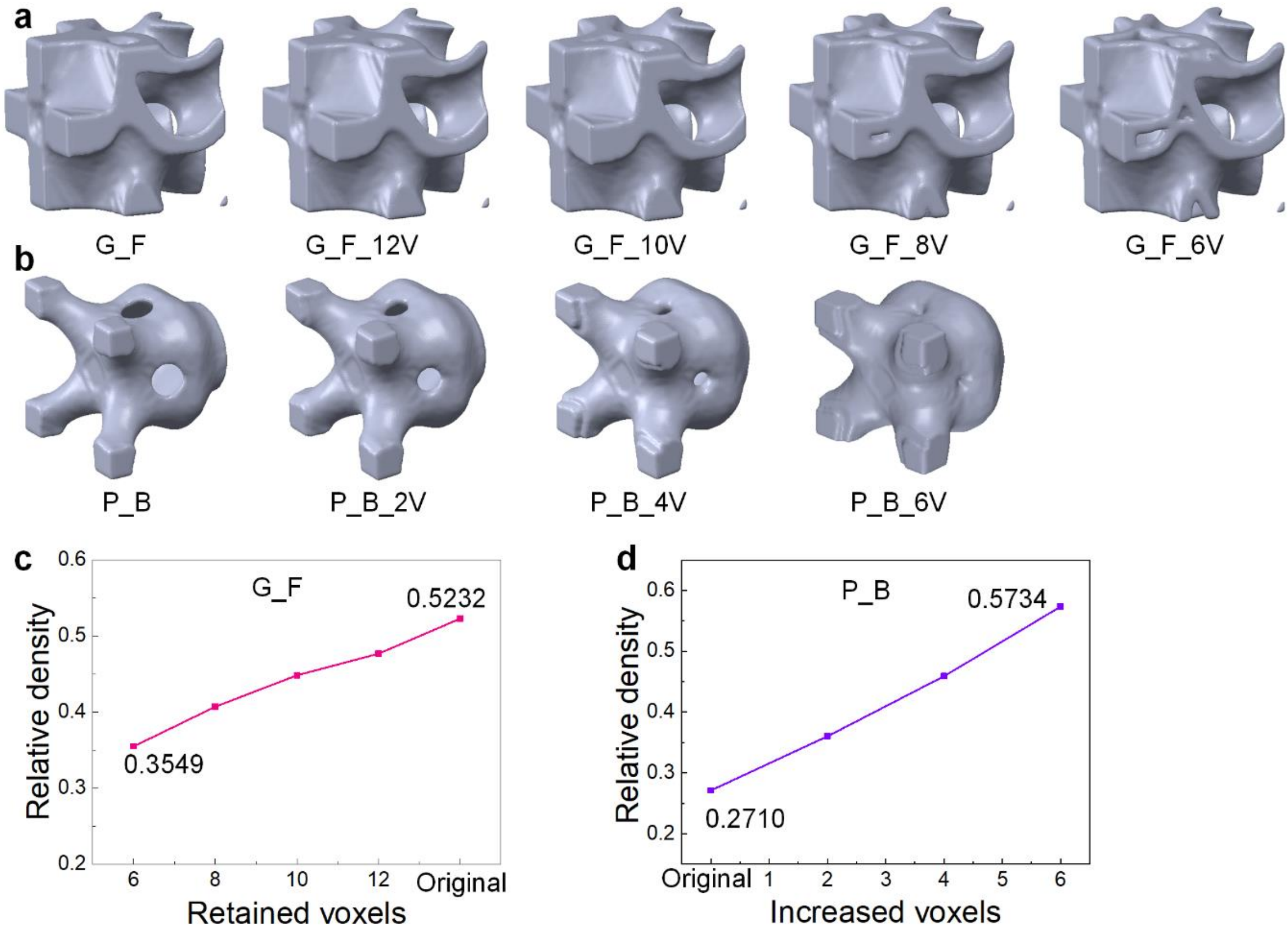

Supplementary Figure 9. Relative density regulation of transitional unit cells. (a) G_F transitional cell, (b) P_B transitional cell, (c) Relationship between the relative density of the G_F transitional cell and the adjusted voxel size, (d) Relationship between the relative density of the P_B transitional cell and the adjusted voxel size. Source data are provided as a Source Data file.

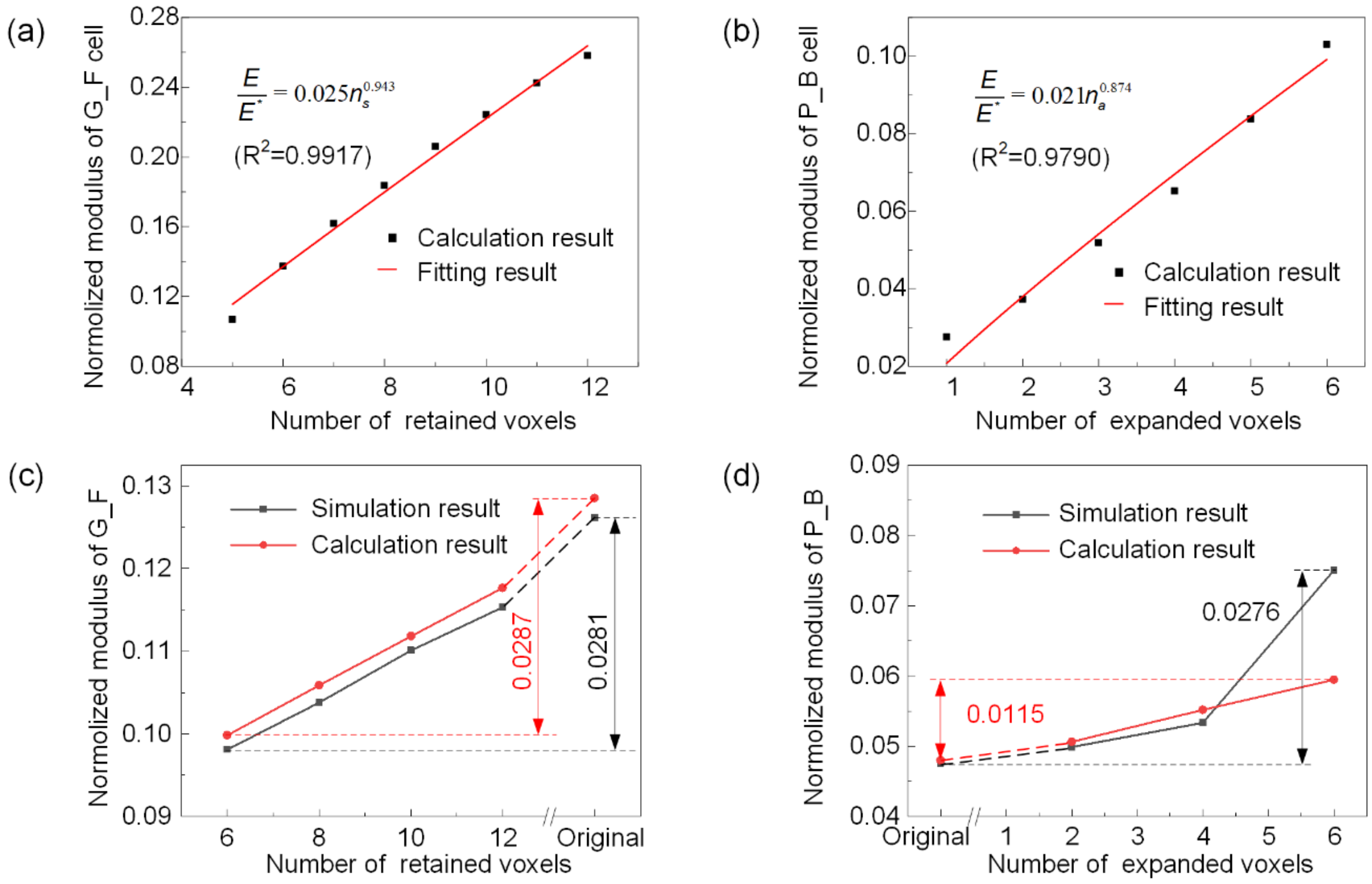

Supplementary Figure 10. The stiffness regulation results for hybrid structures. (a) and (b) fitting results of the normalized modulus for the G_F and P_B transition unit cells at varying levels of voxel retention and dilation, respectively. (c) and (d) The comparison between theoretically calculated and FEM-simulated values for the overall compressive modulus of the hybrid structures under voxel erosion and voxel dilation, respectively. Source data are provided as a Source Data file.

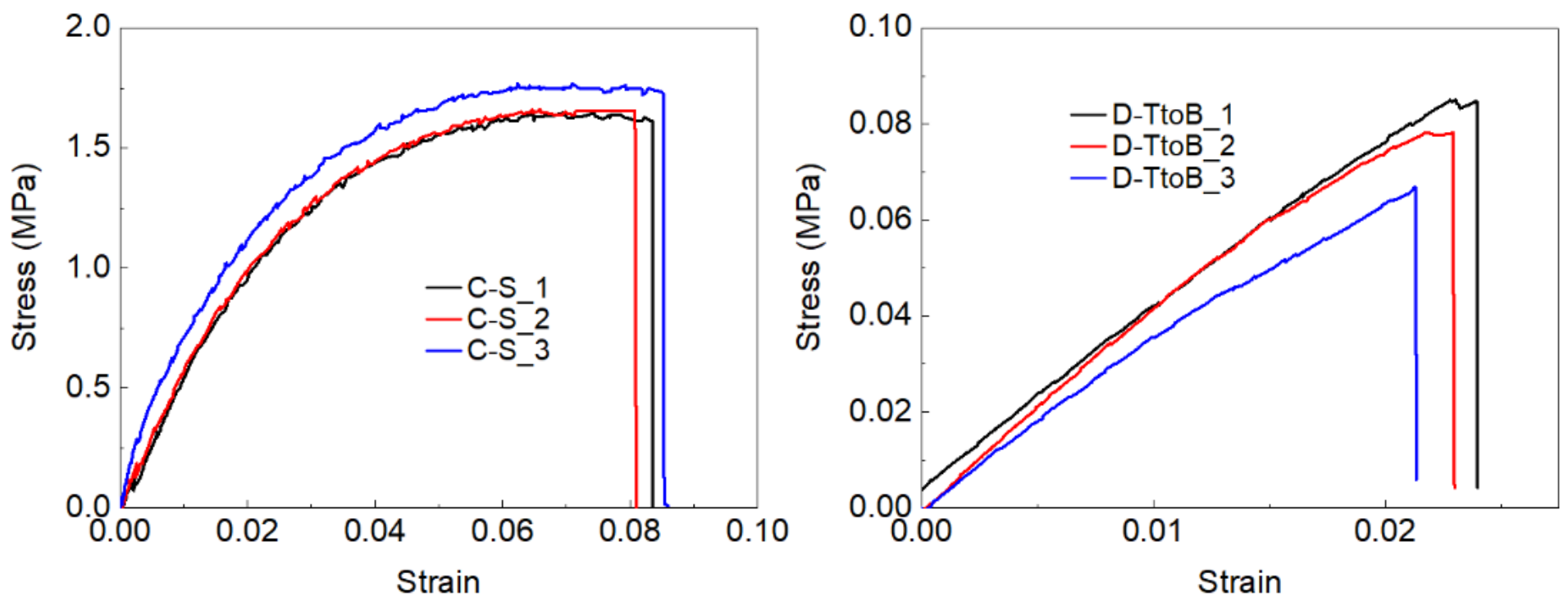

Supplementary Figure 11. The stress-strain curves obtained from repeated uniaxial tensile tests of tendon-to-bone connection structure. Source data are provided as a Source Data file.

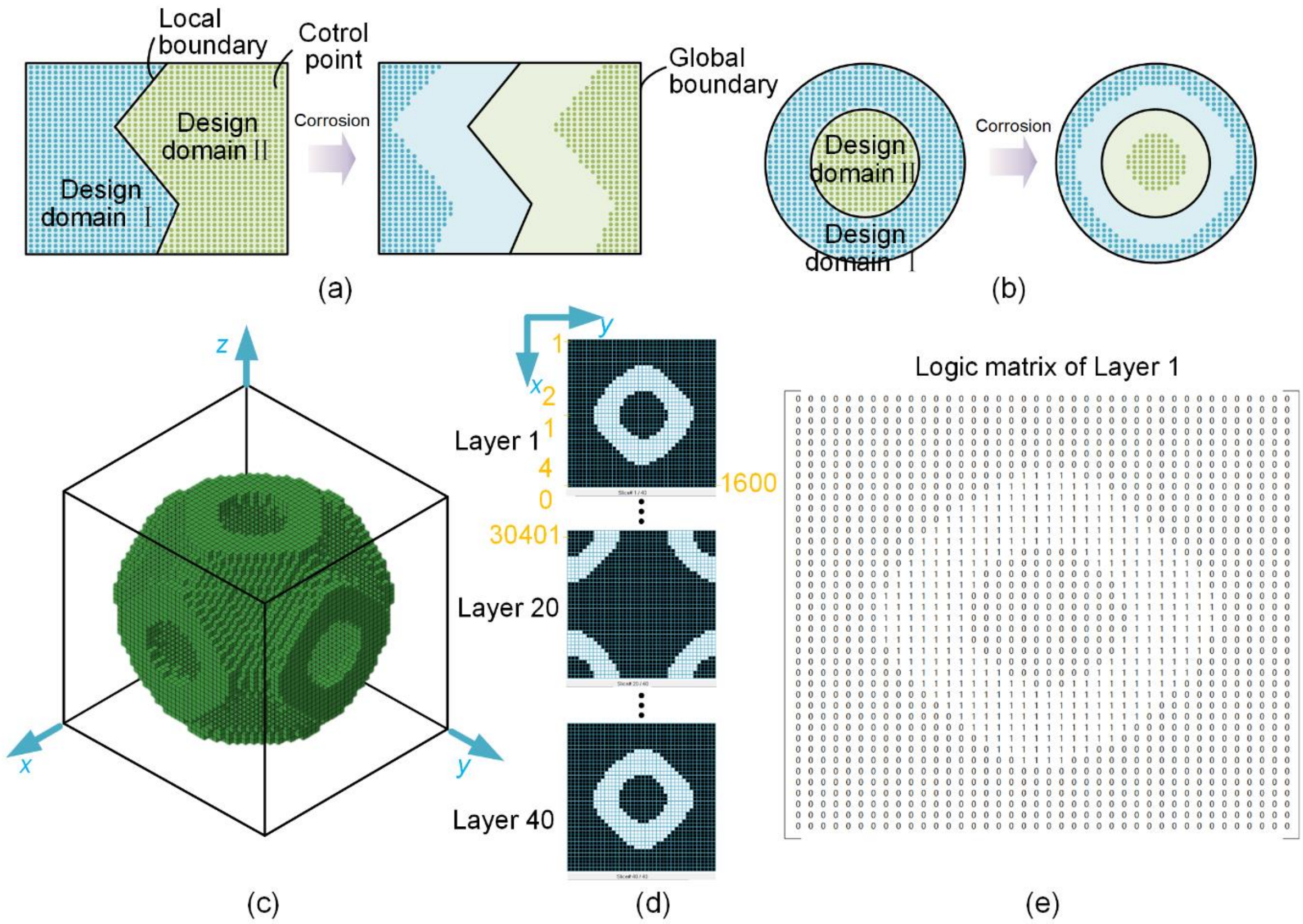

Supplementary Figure 12. Principle of voxel analysis for generating 3D-printed hybrid structures, including: (a, b) Schematic representations of a rectangular and circular design domain, processed using control points. (c, d) Voxel representation and layer-wise distribution within a TPMS structure. (e) Logical matrix representation of voxel distribution.

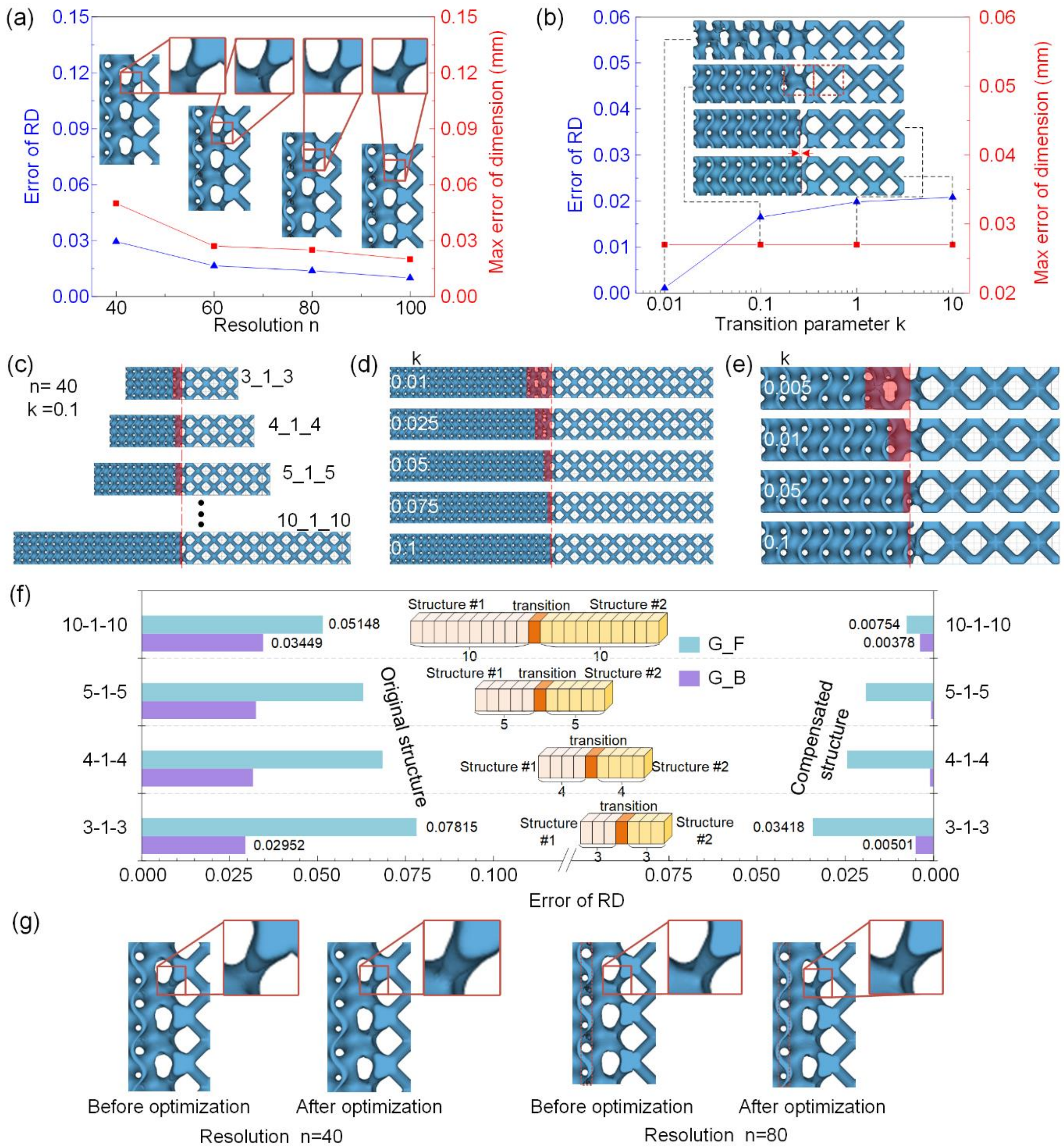

Supplementary Figure 13. The effect of the relevant parameters on the geometrical properties of the generated hybrid structures, including: (a, b) Relative density error and maximum size error as a function of resolution n and transition parameter k, respectively. (c) Effect of the number of unit cells on the transition region length. (d, e) the effect of transition parameter k on transition region length when the number of unit cells is 10 and 3, respectively. (f) Relative density errors in G_F and G_B hybrid structures before and after compensation, for different numbers of single cells. (g) Comparison of structural details in the transition regions of the G_B hybrid structure, before and after optimization. Source data are provided as a Source Data file.

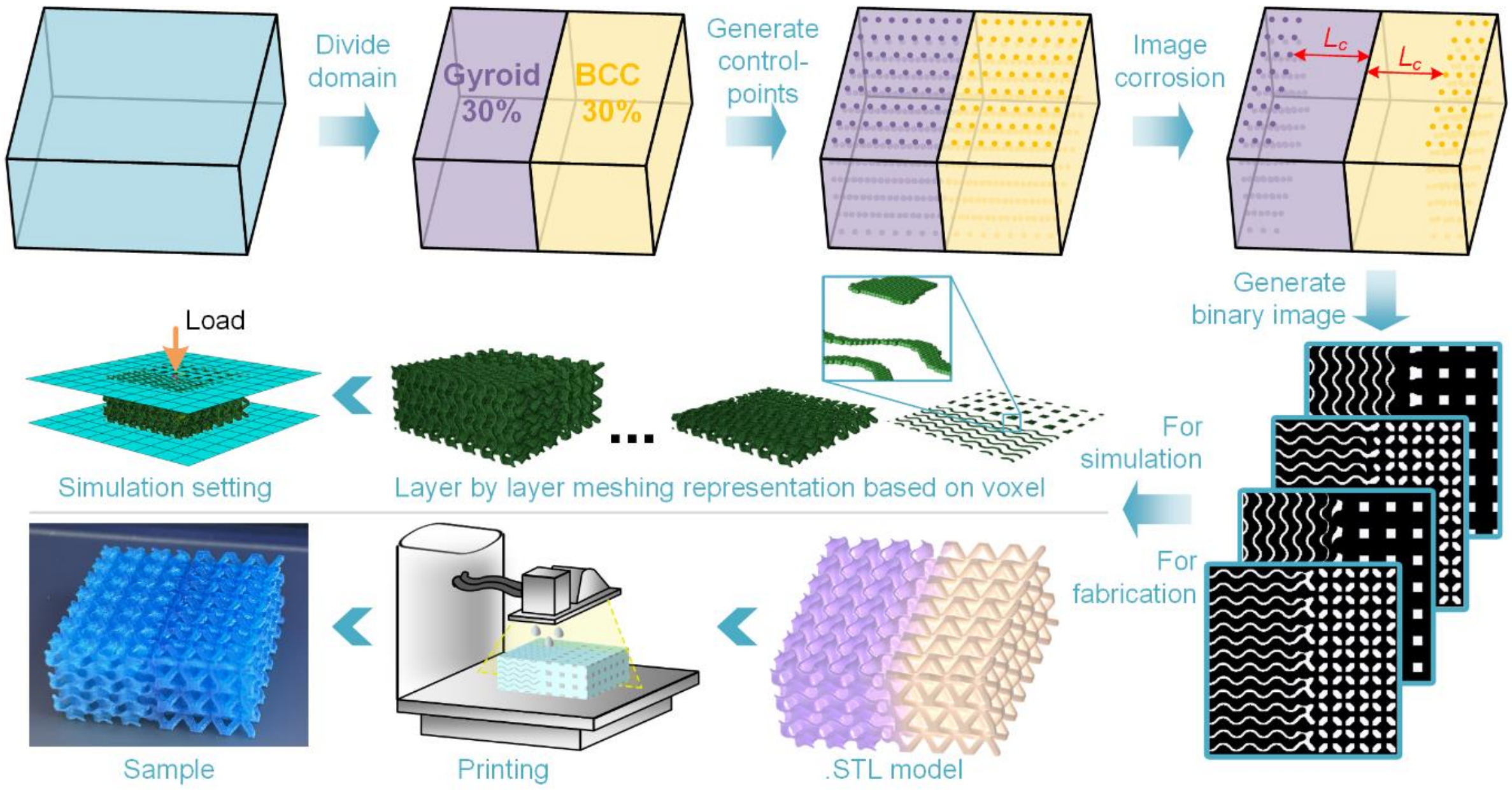

Supplementary Figure 14. A schematic illustration of the generation of direct printable and simulation-ready files for hybrid structures: the first row shows control point-based design domain partitioning and simplification of control points to improve arithmetic power. After generating the layer-by-layer material logic matrix representation of the structure in the lower left corner, it is converted into ".INP" and ".STL" files for computational simulation and fabrication, respectively.

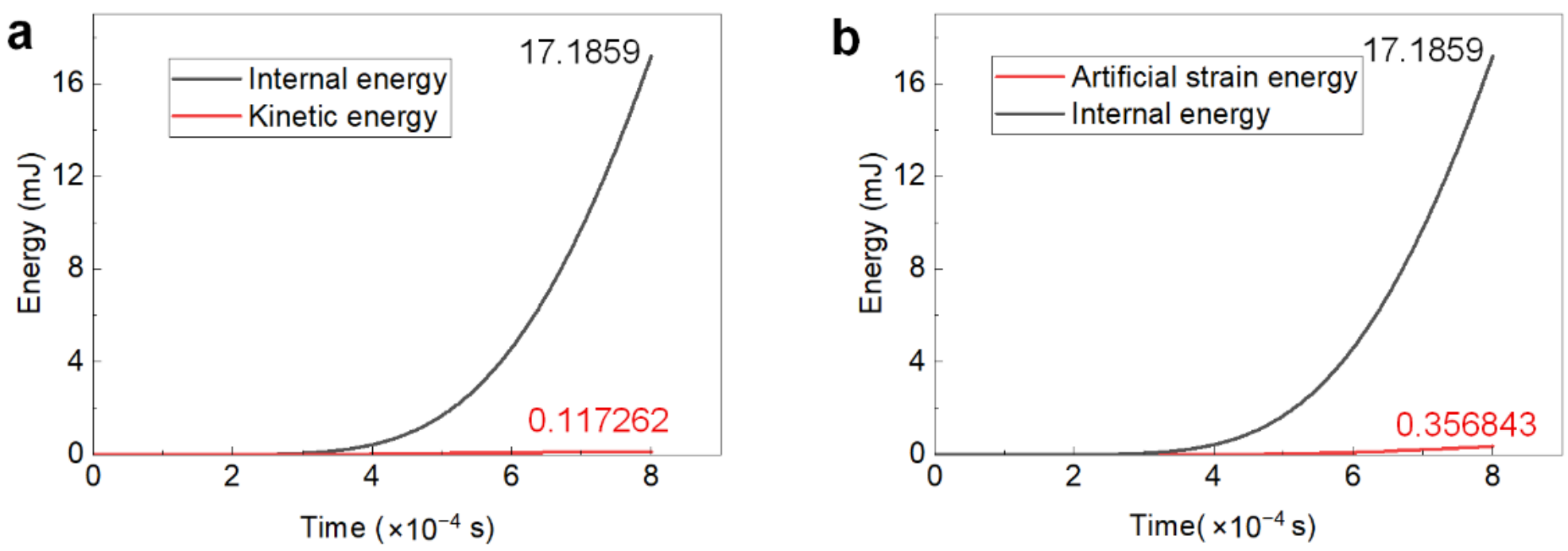

Supplementary Figure 15. The analysis of FEM setting: (a) Internal energy and kinetic energy for quasi-static analysis. (b) Internal energy and Artificial strain energy for hourglassing analysis. Source data are provided as a Source Data file.

Supplementary References

[1] L.J. Gibson, Cellular solids, Mrs Bulletin 28(4) (2003) 270-274.

[2] G. Dong, Y. Tang, Y.F. Zhao, A 149 line homogenization code for three-dimensional cellular materials written in matlab, Journal of Engineering Materials and Technology 141(1) (2019).

[3] W.E. Lorensen, H.E. Cline, Marching cubes: A high resolution 3D surface construction algorithm, Seminal graphics: pioneering efforts that shaped the field1998, pp. 347-353.

[4] T.S. Newman, H. Yi, A survey of the marching cubes algorithm, Computers & Graphics 30(5) (2006) 854-879.

[5] D.-J. Kroon, Smooth triangulated mesh (https://www. mathworks. com/matlabcentral/fileexchange/26710-smooth-triangulated-mesh), MATLAB Central File Exchange, Retrieved June 15 (2020).

[6] F.X. Ren, C.D. Zhang, W.H. Liao, T.T. Liu, D.W. Li, X. Shi, W.M. Jiang, C. Wang, J.F. Qi, Y. Chen, Z. Wang, Transition boundaries and stiffness optimal design for multi-TPMS lattices, Materials & Design 210 (2021) 110062.
