# Supplementary material for: Strategy for hybrid connection of porous strut and triply periodic minimal surface lattice structures
Source: Nat Commun. 2026 Oct 5;17:9699. doi: 10.1038/s41467-026-77560-7 (PMC13639051; doi:10.1038/s41467-026-77560-7)
Supplement: Supplementary file 3 [file 41467_2026_77560_MOESM2_ESM.pdf]

Description of Additional Supplementary Files

File Name: Supplementary Movie 1
Description: Deformation of Uniform Structures under Compression Tests.

File Name: Supplementary Movie 2
Description: Deformation of Hybrid Structures under Compression Tests.

File Name: Supplementary Movie 3
Description: Assembly Test of Load-bearing integrated conformal structure.

File Name: Supplementary Movie 4
Description: Tensile Test for Hybrid Tendon-to-Bone Structure.

File Name: Supplementary Movie 5
Description: Tensile Test for G-Sheet, S-Strut and Their Hybrid Structure.

File Name: Supplementary Movie 6
Description: Torsion and Biaxial Test for G-Sheet, S-Strut and Their Hybrid Structure.

File Name: Supplementary Movie 7
Description: Bending Test for G-Sheet, S-Strut and Their Hybrid Structure.

File Name: Supplementary Code 1
Description: The code for generating continuous heterogeneous hybrid lattice structures.
